# Multi-State Redox and Light-Driven Switching of Pseudorotaxanation and Cation Shuttling

**DOI:** 10.1021/jacs.5c00997

**Published:** 2025-04-11

**Authors:** Robert Hein, Yohan Gisbert, Ben L. Feringa

**Affiliations:** † Stratingh Institute for Chemistry, University of Groningen, Nijenborgh 3, Groningen 9747 AG, the Netherlands; ‡ Organic Chemistry Institute, University of Münster, Corrensstraße 40, Münster 48149, Germany

## Abstract

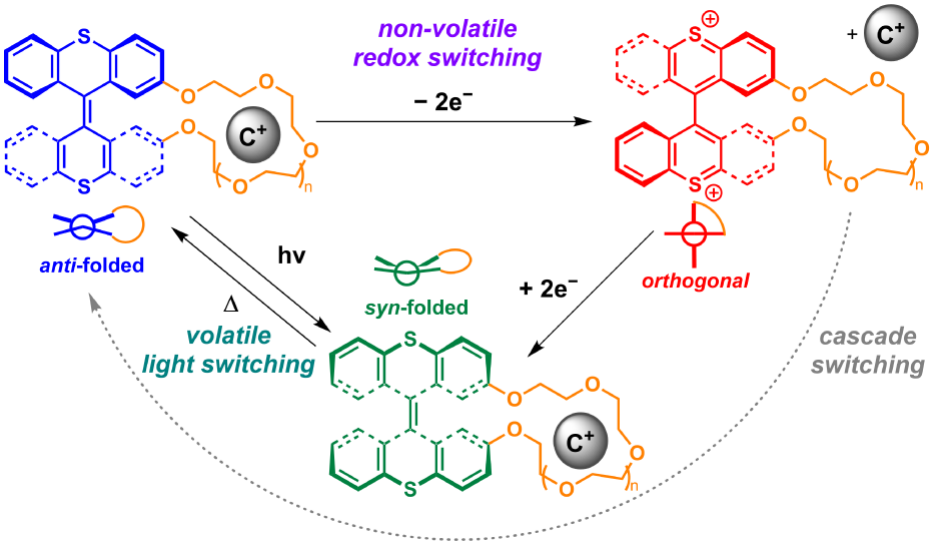

The modulation of molecular recognition underpins numerous wide-ranging applications and has inspired the development of a myriad of switchable receptors, in particular photo- or redox-responsive hosts. Herein, we report a highly versatile three-state cation receptor family and switch system based on an overcrowded alkene strapped with crown ethers, which can be switched by both redox and light stimuli, thereby combining the advantages of both approaches. Specifically, the neutral switches can be quantitatively converted between *anti-* and *syn-*folded receptor geometries by irradiation, leading to the discovery of a significant increase or decrease in cation binding affinity, which was exploited to shuttle the pseudorotaxane-forming dibenzylammonium guest between the switchable crown ethers of slightly different sizes. Alternatively, two-electron oxidation to the orthogonal, dicationic, nonvolatile state completely turns off cation binding to the host, thereby ejecting the guest. Upon reduction, the metastable *syn-*folded state is first formed, which then thermally relaxes, resulting in a unique, autonomous, and cation-dependent multistate switching cascade.

## Introduction

From the early days of supramolecular chemistry,^1−3^ the reversible modulation of various molecular properties has received significant attention in distinct fields, including molecular switches and machines,^4−6^ stimuli-responsive materials,^7,8^ molecular logic,^9,10^ and sensing.^11,12^ The ability to judiciously switch ON or OFF noncovalent intermolecular interactions is a particularly important goal in this context, as it enables control over the formation or breakup of supramolecular structures at various length scales ranging from small molecule host–guest complexes^13,14^ to larger self-assemblies such as supramolecular polymers or gels.^15−17^ This, in turn, enables numerous applications ranging from the construction of molecular machines^18−21^ to transmembrane transporters,^22−24^ extractants, and sensors^25,26^ to smart materials and interfaces.^27,28^

Such control over intermolecular interactions can be achieved by various external means, including physical, chemical, electrochemical, or optical stimuli.^4,6,24,29^ Light- and redox-driven switching of noncovalent interactions has individually received particular attention as these constitute convenient means to control supramolecular interactions with high spatiotemporal precision. Following pioneering work by Shinkai and coworkers,^2,30^ light-induced conformational changes have been exploited in the development of numerous photoresponsive molecular receptors, whereby reversible back-switching to the initial conformational state can be achieved either by light of a different wavelength or thermally.^31−35^ If the latter is comparably fast, the bistability of the system is compromised; however, this can enable dissipative (or volatile, i.e., metastable) switching, which represents an increasingly relevant approach to generate more complex, time-dependent switching, assembly, and out-of-equilibrium behavior.^36−41^

In contrast, the vast majority of redox-driven switches do not affect intermolecular interactions through inherent conformational changes but rather by switching charge, which strongly influences the strength of numerous noncovalent interactions. This has been famously exploited in numerous redox-driven (interlocked) molecular shuttles and pumps^14,19,42−46^ as well as a wide range of redox-switchable receptors and electrochemical sensors.^11,26,29,47−49^ Advantageously, unlike photoswitching, redox switching between different charge states occurs quantitatively and is nonvolatile (i.e., stable), as long as the chemical integrity of all states is maintained.

Conceivably, the combination of both photo- and redox-switching approaches in a single receptor would enable versatile, multistate control over guest binding with both volatile and nonvolatile control, respectively, but it remains notably underdeveloped.^50−54^ This, in turn, is expected to be highly significant for the construction of multistimuli-responsive molecular machines, sensing, and systems chemistry.^37,39,55,56^

**Figure 1:**
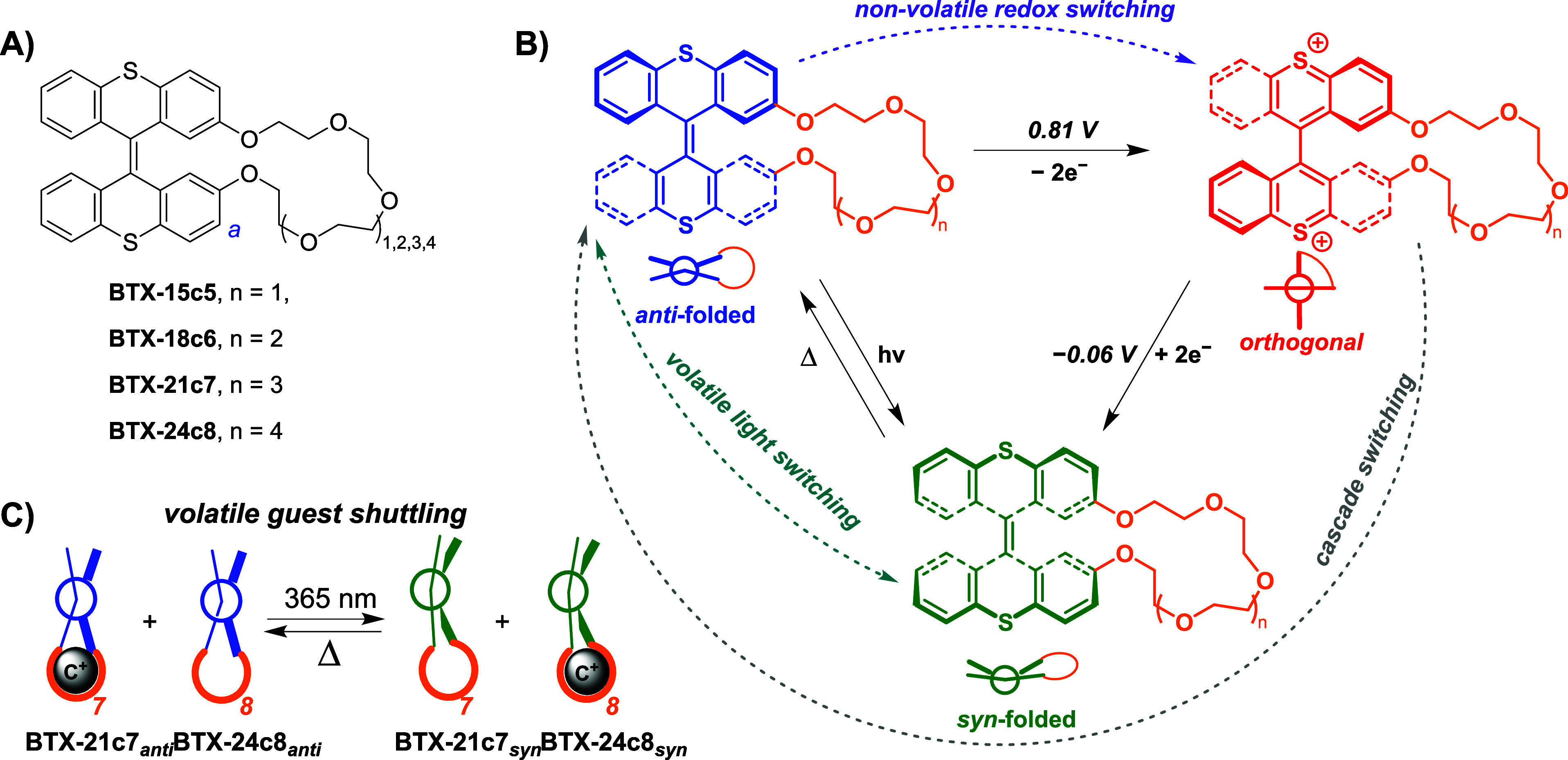
(A) Structure of **BTX-crown ethers**. (B) Multistate redox- and light-driven switching of **BTX-crown ethers** between three quantitatively accessible switching states that each possess different geometries and cation affinities. (C) Schematic depiction of volatile light-driven guest shuttling between **BTX-crown ethers** of different sizes. In the absence of light, fast thermal reversion is observed, as the *syn-*folded states are metastable.

Here, we address this challenge in the proof-of-principle design of a family of multistate redox- and light-responsive crown ether cation receptors using the bisthioxanthylidene (BTX) overcrowded alkene switch scaffold (Figure 1A).^57−61^ Building on our earlier studies, and as shown in Figure 1B, the parent BTX unit can act as a highly versatile multistate conformational switch. Specifically, due to steric crowding in the fjord region of the alkene, the most stable conformation of the native, neutral switch is characterized by two oppositely folded switch halves (Figure 1B, top left).^62,63^ This neutral *anti*-folded state can be converted to a stable dicationic state via simultaneous two-electron oxidation,^64^ wherein the planar, aromatic thioxanthylium rotors are now linked via a single bond and adopt an orthogonal conformation (Figure 1B, top right), as previously confirmed by single-crystal XRD.^57^ Reduction of this dication first quantitatively forms the neutral, metastable *syn-*folded state, wherein both halves are folded toward each other (Figure 1B, bottom),^63^ which thermally relaxes back to the *anti-*folded state, thus resulting in a predefined switching cascade. In turn, this neutral, higher-energy *syn-*folded conformer can also be generated by irradiation of the *anti-*folded state with UV light.^57,65^ Importantly, all these interconversions proceed quantitatively and with a very high degree of reversibility, whereby not only the geometry of each switching state is changed significantly but also their charge/polarity as well as color and fluorescence.^57^

We envisioned that by appending a crown ether strap to the BTX scaffold, its unique multistate switching properties could be leveraged to construct versatile receptors in which cation binding affinity can be fine-tuned. Specifically, cation binding to the neutral *anti-*folded state is typically strong but is expected to completely switch off upon oxidation to the dication state, inducing guest expulsion. Similarly, light-driven population of the *syn*-folded state also induces significant changes in cation binding affinity, which depending on the size of the crown ether strap and specific cation complexation is either stronger or weaker than that of the *anti*-folded conformer. In contrast to the redox switching process, which is nonvolatile, this syn-folded state can only be maintained under continuous light irradiation and is thus a volatile state. We discovered that this enables reversible shuttling of an ammonium guest between BTX-crown ether receptors of different sizes (Figure 1C).

## Results and Discussion

### Design and Synthesis of BTX-Crown Ether Switches

The design of the cation-receptive switches features a BTX core functionalized at its 2- and 2’- positions with ethylene glycol chains of different lengths, containing between 5 and 8 oxygen atoms in the crown ether motif (Figure 1A). This strapping of both switch halves via the ethylene glycol chain not only affords symmetric receptors but also prevents switching to the *E*-isomer, simplifying the operation and investigation of these switches. We showed that **BTX-15c5** and **BTX-18c6** bind to various alkali metal salts;^66^ however, thus far, neither their redox nor optical (switching) properties have been explored. The synthesis of these receptors, including the novel, extended **BTX-21c7** and **BTX-24c8**, was carried out by an improved procedure, as shown in Scheme S1 and detailed in the Supporting Information. While the systems studied herein are nondirectional switches, various ethylene glycol-containing (unidirectional) motors are also known in the literature.^34,67−70^

### Light-Driven Switching of Cation Binding

**Figure 2:**
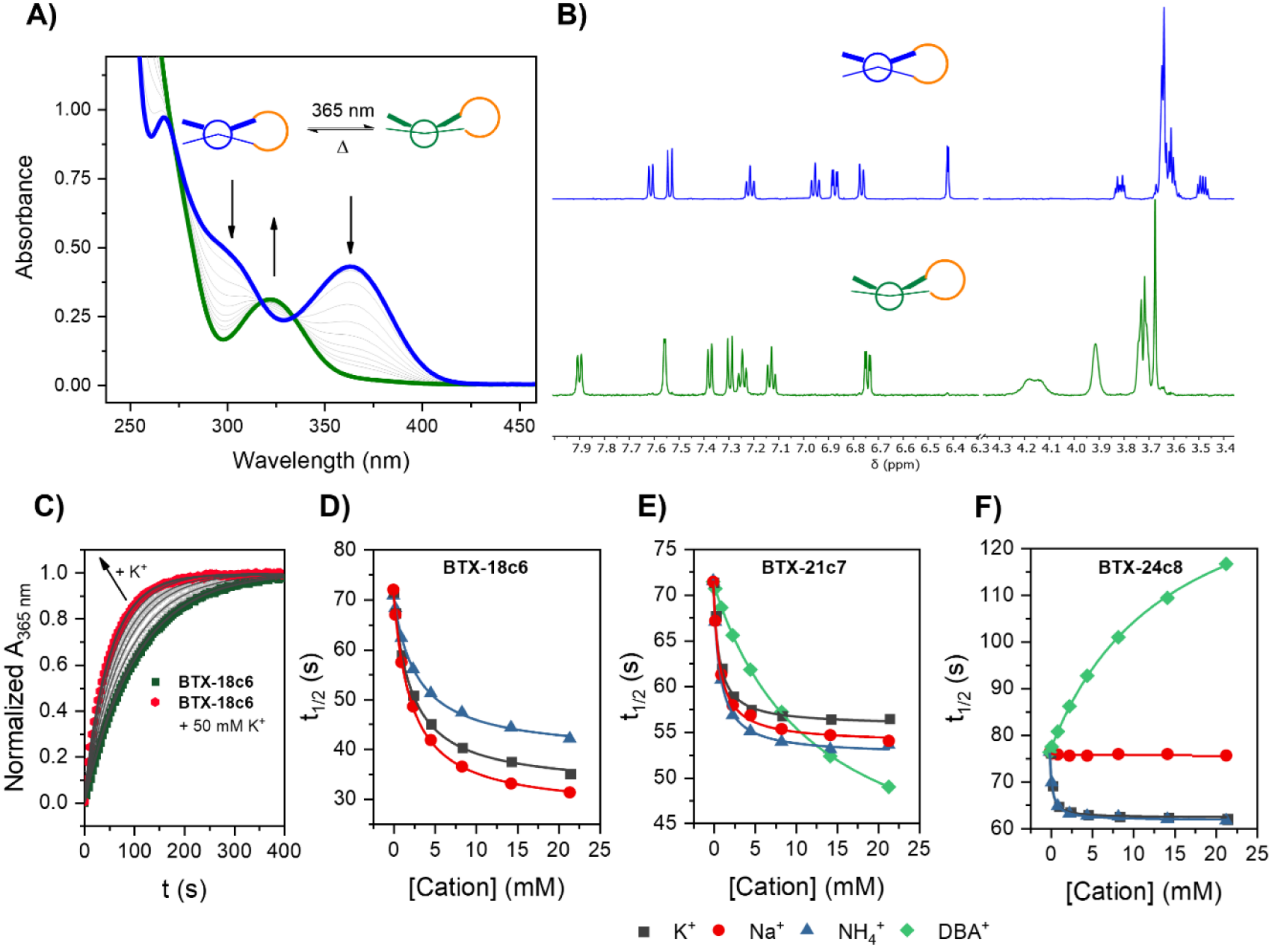
(A) UV–vis spectra of 50 μM **BTX-18c6** upon irradiation with 365 nm light. (B) The corresponding ^1^H NMR spectra of **BTX-18c6** in the *anti-*folded state (top) and in the *syn-*folded state (bottom), obtained via *in situ* irradiation with 365 nm light, show quantitative conversion between the two folded states. (C) Normalized absorbance change at 365 nm upon thermal relaxation of **BTX-18c6** from the *syn-*folded to the *anti-*folded state in the presence of increasing concentrations of K^+^. (D–F) Thermal half-lives of the syn-folded states of **BTX-18c6**, **BTX-21c7**, and **BTX-24c8** as a function of cation concentration. All experiments were carried out using 50 μM host at 25 °C in CH_3_CN/CH_2_Cl_2_ 7:3 (or the corresponding deuterated solvents). See Table S1 for *t*_1/2_ values of all receptors, including **BTX-15c5**.

Gratifyingly, the strapping of the BTX core with the crown ether has no strong influence on its photoswitching properties. Specifically, all **BTX-crown ether** receptors displayed similar UV–vis spectra (λ_max_ = 364 nm) that are characteristic of BTX in the *anti-*folded conformation (Figure 2A).^57,59^ Upon irradiation with UV light, these lowest-energy bands shift hypsochromically to λ_max_ = 322 nm, which is in excellent agreement with prior reports on BTX switches and confirms unimpeded formation of the metastable *syn*-folded state.^57^ This process is highly reversible, whereby the relaxation occurs quickly at room temperature (*vide infra*); i.e., the *syn*-folded state is highly volatile. Importantly, this light-driven switching process is also fully quantitative, as elucidated by ^1^H NMR (Figure 2B). Complete photochemical conversion to the *syn-*folded state is in good agreement with the literature^57^ and is a result of the one-way photoswitching process; irradiation with light only promotes the *anti → syn* conversion, while the reverse reaction is a purely thermal process.^65^ As a result, full conversion to the *syn-*folded state is, in principle, always possible, as long as the light intensity is high enough (see, e.g., Figures S18 or S20).

As shown in Table S1, the thermal half-life times *t*_1/2_ for the thermal backward isomerization from *syn-* to *anti-*folded states were virtually identical for **BTX-18c6**, **BTX-21c7,** and **BTX-24c8** with *t*_1/2_ ≈ 72 s, which is very similar to that of **BTX-OMe** (*t*_1/2_ = 87 s, as ≈1:1 *E*/*Z* mixture). Only **BTX-15c5** displayed noticeably slower thermal relaxation, with *t*_1/2_ = 180 s, indicating that the shorter ethylene glycol strap influences the activation barrier for structural interconversions to some degree,^71^ an unexplored but useful approach in tuning the relative stability/half-life time of such bistricyclic aromatic alkenes.

To investigate the influence of cation binding on the optical (switching) properties of the receptors, detailed titration studies were carried out for all receptors with Na^+^, K^+^, NH_4_^+^, and dibenzylammonium (DBA^+^) (all as PF_6_^–^ salts) in CH_3_CN/CH_2_Cl_2_ 7:3 (v/v) (Figure S11). This solvent system was chosen to ensure good solubility of the receptors, as well as the cation salts, in all switching states. The DBA^+^ cation forms, as expected, pseudorotaxanes with the larger **BTX-21c7** and **BTX-24c8**, as evidenced by ^1^H NMR studies, while no significant interactions were observed with **BTX-18c6**, indicating that its cavity is too small to accommodate DBA^+^ (see Figures S14–S16).

In the presence of these cations, notable changes in the speed of thermal relaxation were observed in all cases (Figure 2C). For example, the addition of all cations to **BTX-18c6** or **BTX-21c7** induced marked increases in the backward relaxation speed (*syn → anti*), as reflected by up to 62% lower *t*_1/2_ values (Figure 2D–F and Table S1).

Unexpectedly, the smaller **BTX-15c5** displayed the opposite effect for all tested cations, that is, a very significant decrease in the speed of thermal relaxation, with *t*_1/2_ > 3000 s in the presence of excess K^+^, corresponding to an almost 17-fold slowdown of the *syn* → *anti* conversion. A remarkable discovery is therefore that the extension of **BTX-15c5** by a single ethylene glycol unit, to give the very similar **BTX-18c6,** induces a complete reversal in how cation binding affects the energetic landscape of the structural interconversion of the folded states. This behavior is even more complex in **BTX-24c8**; the addition of Na^+^ induces no notable changes, while K^+^ and NH_4_^+^ induce moderate decreases of *t*_1/2_ (Figure 2F). Strikingly, in the presence of DBA^+^, a 2-fold increase of *t*_1/2_ was observed, corresponding to a significant slowdown of relaxation.

**Figure 3:**
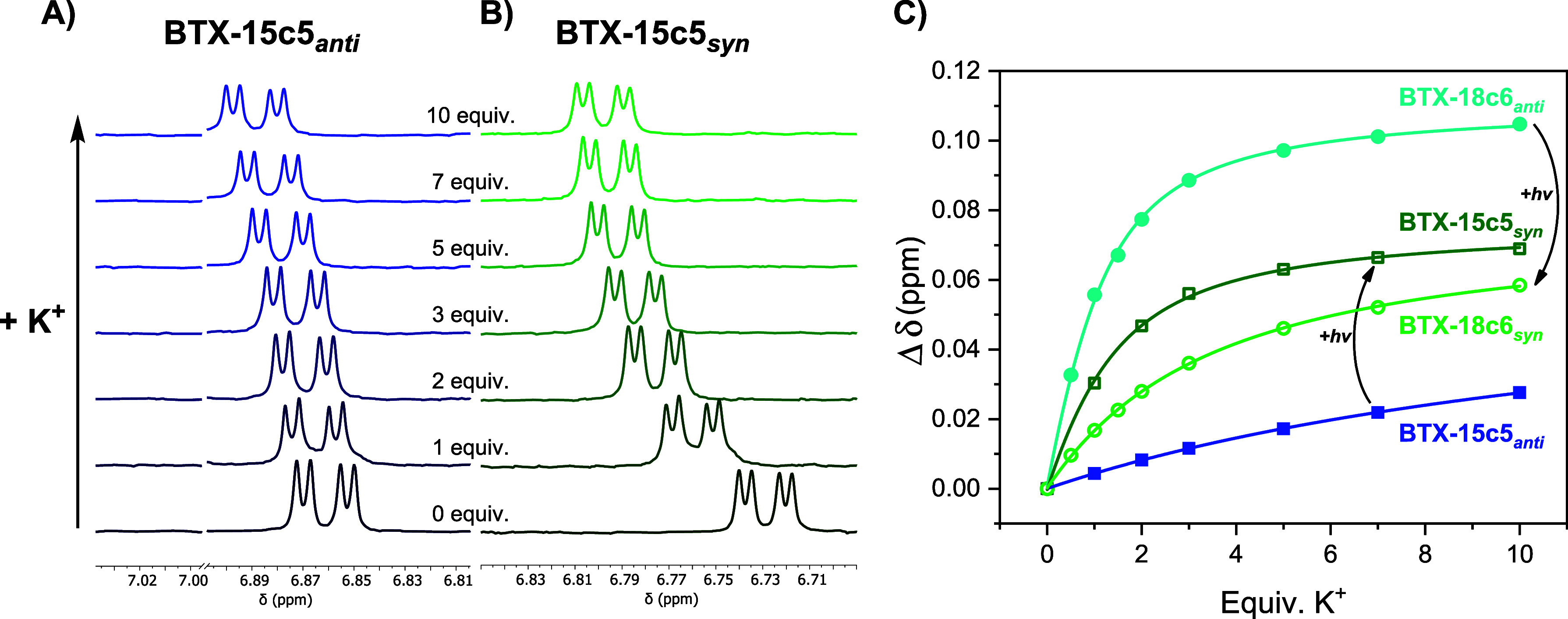
(A,B) Partial, stacked ^1^H NMR spectra of proton *H*_*a*_ of 1 mM **BTX-15c5** in CD_3_CN/CD_2_Cl_2_ 7:3 upon addition of K^+^ in the *anti*-folded or *syn*-folded states, respectively. See Figure 1A for the proton assignment. (C) Changes in chemical shift of *H*_*a*_ as a function of K^+^ concentration of **BTX-15c5** or **BTX-18c6** (1 mM in CD_3_CN/CD_2_Cl_2_ 7:3) in both *anti*-folded or *syn*-folded states. The solid lines represent fits to a 1:1 host–guest stoichiometric binding model.

We hypothesized that this intriguing behavior arises as a result of significantly different cation binding affinities to the two distinct folded states. This was ascertained by ^1^H NMR *in situ* irradiation titration experiments, which enabled quantification of the cation binding constants to both the stable *anti-*folded state and the unstable *syn*-folded state for selected receptor/cation pairs (Figures S17–S32). Indeed, notable differences in cation binding affinities between the two folded states were observed. For example, **BTX-15c5***_anti_* displayed only very weak 1:1 host–guest stoichiometric binding to K^+^ (*K_anti_* = 50 M^–1^), while **BTX-15c5***_syn_* bound the same cation much more strongly (*K_syn_* = 1220 M^–1^), corresponding to a 24-fold increase in binding upon irradiation (Figure 3 and Table 1).

**Table 1:** K^+^ Binding Constants *K* (M^–1^) of **BTX-Crowns** in *Anti-*Folded and *Syn-*Folded States Obtained by ^1^H NMR Titrations in CD_3_CN/CD_2_Cl_2_ 7:3 (with *In Situ* Irradiation with 365 nm Light)

	**BTX-15c5**	**BTX-18c6**	**BTX-21c7**	**BTX-24c8**
*Anti-*folded	50	1920	1310	6730
*Syn-*folded	1220	310	1450	6020

Interestingly, this trend was completely reversed for **BTX-18c6**, in which binding was 6-fold stronger to the *anti-*folded state (*K_anti_* = 1920 M^–1^, *K_syn_* = 310 M^–1^). In the even larger **BTX-21c7** and **BTX-24c8,** the difference in binding affinity for the two folded states was notably attenuated (at least for the small, inorganic K^+^, *vide infra*), most likely reflecting the much higher degree of flexibility of the longer crown ether straps. At the same time, increasing the length of the crown ether from **BTX-21c7** to **BTX-24c8** induced a significant increase in the K^+^ binding affinity, which is also most likely related to the relatively large degree of flexibility of the strap, enabling more efficient multidentate binding and “wrapping” of the crown ether around this alkali metal cation. Of note is that this behavior is somewhat unexpected, as the parent (dibenzo)-crown ethers typically display a decreased K^+^ affinity for the larger (DB)-24-crown-8 receptor.^72,73^ This indicates that the receptors reported herein differ somewhat in terms of structure and flexibility from their parent analogues.

Importantly, the difference in cation binding constants of K^+^ (and also DBA^+^, *vide infra*) to the two folded states is in excellent correlation with the change in speed of the thermal *syn → anti* relaxation. In all cases where the *anti-*folded state binds the cationic guest more strongly than the *syn-*folded state, significant speed-up of the backward relaxation is observed (e.g., for K^+^ with **BTX-18c6**), while the opposite holds if binding to the *syn-*folded state is preferred (e.g., for K^+^ with **BTX-15c5**). As such, the stronger binding state is not only thermodynamically favored but also kinetically stabilized/preferred. In the case of an *anti-*folded binding preference, this leads to a “self-resetting” of the system upon addition of cations, while *syn-*folded binding preference enhances the lifetime of the metastable state.

These observations can be rationalized by the Bell–Evans–Polanyi principle, as detailed in Figure S12, and enable a qualitative assessment of the relative differences in cation binding to the folded states by consideration of the relaxation kinetics; a speed-up of relaxation is indicative of stronger binding to the *anti-*folded state, while a binding preference for the *syn-*folded state induces slower back-switching.

**Figure 4:**
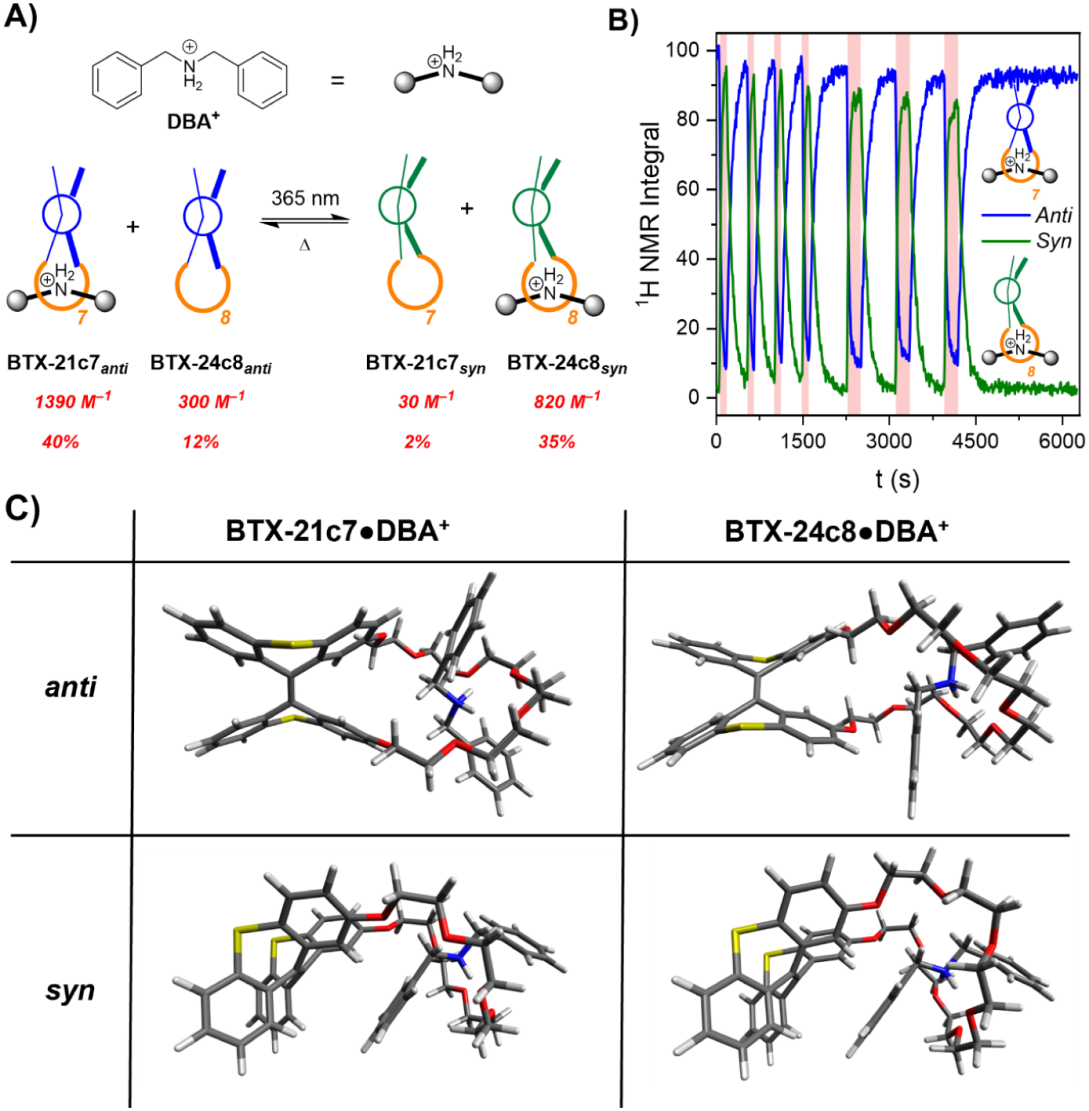
(A) Schematic representation of the shuttling of DBA^+^ in a mixture of **BTX-21c7** and **BTX-24c8**, where, in the absence of light, the DBA^+^ preferentially forms a pseudorotaxane with **BTX-21c7** in the *anti*-folded state. Upon light-driven switching of both crown ether hosts to the *syn*-folded state, DBA^+^ now preferentially binds to **BTX-24c8*_syn_*** and hence shuttles. The red values show the binding constants and the calculated degree of complexation of DBA^+^ by the respective crown ether in a mixture of 1 mM **BTX-21c7** + 1 mM **BTX-24c8** + 1 mM DBA^+^.^76^ (B) Change in ^1^H NMR integrals of a mixture of 1 mM **BTX-21c7** and 1 mM **BTX-24c8** and of 1 mM DBA^+^ in CD_3_CN/CD_2_Cl_2_ 7:3 upon repeated *in situ* irradiation with 365 nm light (red shaded areas) followed by thermal relaxation. The blue line corresponds to the combined integral for both crown ethers in the *anti*-folded states, while the green line corresponds to the *syn*-folded states. Shuttling is confirmed by detailed comparison of the ^1^H NMR spectra; see Section 4.3. (C) Calculated lowest energy conformers of the complexes of DBA^+^ with **BTX-21c7** and **BTX-24c8** in both the *anti-* and *syn-*folded states, showing formation of pseudorotaxanes in all cases. In spite of the good agreement between experimental and calculated binding energies, we were not able to identify clear structural differences/parameters that can be used to rationalize the relative difference in binding energy. For further discussions, see Section S6 as well as the specific calculation method on Pages S3– and S4.

This is perhaps most interesting for the pseudorotaxane-forming DBA^+^, which significantly speeds up relaxation of the **BTX-21c7***_syn_* switch but slows down **BTX-24c8***_syn_* relaxation, indicating opposite binding alteration upon light switching. Indeed, ^1^H NMR *in situ* irradiation titration experiments confirmed this behavior: irradiation of the DBA^+^ complex of **BTX-21c7**_***anti***_ (*K*_*anti*_ = 1390 M^–1^) to **BTX-21c7**_***syn***_ (*K*_*syn*_ = 30 M^–1^, Figure 4A) results in virtually complete ejection of the cationic guest. In contrast, the exact opposite trend, albeit with a somewhat smaller difference, was observed for **BTX-24c8** (*K_anti_* = 300 M^–1^, *K_syn_* = 820 M^–1^), again highlighting the enormous influence of simple crown ether extension on modulating the binding behavior of these switch receptors.

### Light-Driven Volatile Shuttling and Pseudorotaxanation

As a result of this unexpected observation, light-switching induces the simultaneous ejection of DBA^+^ from **BTX-21c7***and* uptake by **BTX-24c8**, thereby enabling “double” shuttling and pseudorotaxanation of this guest in a system containing all three components, which represents a rare example of a multicomponent stimuli-driven molecular translocation system.^20,74,75^ Specifically, calculations^76^ predict that in a mixture containing 1 mM of each **BTX-21c7** and **BTX-24c8** and DBA^+^, 40% of the DBA^+^ forms a complex with the former host, while only 12% is bound by the larger **BTX-24c8** (Figure 4A). However, upon irradiation, the calculated occupancy of the crown ether hosts changes dramatically; only 2% of DBA^+^ is bound by **BTX-21c7***_syn_*, while a much larger proportion of 35% resides at **BTX-21c7***_anti_*, which corresponds to a significant degree of shuttling of the guest between the two hosts upon switching.

This was experimentally supported by ^1^H NMR *in situ* irradiation studies, which confirmed that in the absence of light, DBA^+^ preferentially binds to **BTX-21c7***_anti_*, while irradiation induces shuttling to **BTX-24c8***_syn_* (see Figures S36–S41 and associated discussions for more details). Importantly, this process is not only volatile—the population of **BTX-24c8***_syn_*•DBA^+^ as long as light is present and quickly reverts in the dark—but is also highly fatigue-resistant over many switching cycles (Figure 4B). While the overall degree of complexation of the DBA^+^ guest is only moderate in the case described above, it should be noted that both the overall fraction of bound guest and the relative shuttling performance can be judiciously tuned by adjusting the concentrations and ratios of all three components (see Figure S42 and Table S2 and associated discussions for further information). Additionally, a similar shuttling system for K^+^ could be designed (Table S3).

To further rationalize these rather unintuitive switching trends, DFT calculations and geometry optimizations were representatively carried out on populations of conformers for the host–guest complexes of DBA^+^ with **BTX-21c7** and **BTX-24c8** in both folded states, using an implicit solvent model of acetonitrile as detailed on Pages S3 and S4. As shown in Figure 4C, these calculated structures confirm the formation of a pseudorotaxane structure in all cases and further showcase hydrogen bonding interactions between the ammonium motif and the crown ether oxygen atoms. Additionally, the calculated energy differences gratifyingly match very well with experimental observations: DBA^+^ binds most strongly to **BTX-21c7***_anti_*, slightly weaker to **BTX-24c8***_syn_* (ΔΔ*G*_*Bind*_ = 1.2 kJ/mol), weaker yet to **BTX-24c8***_anti_* (ΔΔ*G*_*Bind*_ = 5.2 kJ/mol), and only very weakly to **BTX-21c7***_syn_* (ΔΔ*G*_*Bind*_ = 12.6 kJ/mol). See Tables S6 and S7 and associated discussions Pages S49 and S50 for more details. This further confirms that DBA^+^ indeed shuttles from **BTX-21c7***_anti_* to **BTX-24c8***_syn_* upon irradiation. Despite the very good agreement of the DFT-calculated values with the experimental ones, we could not identify any obvious structural parameters on the lowest-energy conformers of both isomers of the **BTX-21c7** and **BTX-24c8** complexes which would allow a rationalization of the observed trends.

Lastly, it should be noted that while the light-driven switching/shuttling system presented herein is dissipative in the sense that the switch itself dissipates light energy through its switching (and subsequent relaxation), the complexation itself is not dissipative and does not induce a special out-of-equilibrium state/distribution. Rather, the concentration of all host–guest complexes (in either of the folded states) is determined only by the individual equilibrium constants and is not inherently tied to the presence of light. As such, the host–guest complexes represent thermodynamic assemblies under dissipative conditions.^77^

### Redox Switching of Cation Binding

**Figure 5:**
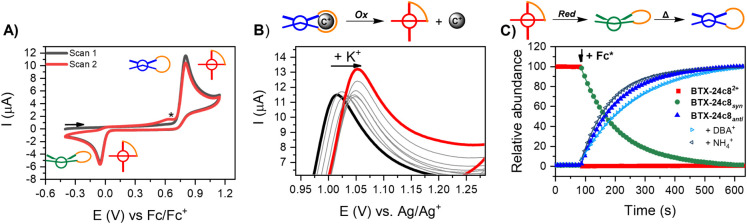
(A) CVs of 0.5 mM **BTX-15c5** in CH_2_Cl_2_, 100 mM TBAPF_6_ at ν = 100 mV/s. The small shoulder peak marked with an asterisk can be ascribed to oxidation of the *syn*-folded state of the receptor. (B) CVs of 0.25 mM **BTX-18c6** in CH_3_CN/CH_2_Cl_2_ 7:3, 100 mM TBAPF_6_ upon addition of increasing concentrations of KPF_6_ at ν = 100 mV/s (oxidation peak only). (C) Relative abundance of all three switching states of **BTX-24c8** in CH_3_CN/CH_2_Cl_2_ 7:3 upon chemical reduction of **BTX-24c8^2+^** with 3 equiv. Fc* as determined by UV–vis spectroscopy (Figure S13), showing a specific, autonomous switching sequence from the dicationic state to first the *syn-*folded state, which then thermally relaxes to the *anti-*folded state. This relaxation is slowed in the presence of DBA^+^ and sped up in the presence of NH_4_^+^ (21.4 mM in each case).

Voltammetric studies of the **BTX-crown ethers** in CH_2_Cl_2_ revealed redox signatures that are not only qualitatively but also quantitatively analogous to the parent **BTX-OMe**,^59^ as shown in Figure 5A and Table S4, crucially confirming that the crown ether strap, regardless of its length, has no significant effect on the redox-switching properties of the BTX core. Specifically, in all cases, a single redox wave in the anodic scan direction was observed at ∼ +0.81 V (vs Fc/Fc^+^), corresponding to simultaneous two-electron oxidation. This is associated with significant geometric rearrangements from the initially neutral, *anti-*folded BTX to the orthogonal BTX^2+^ motif.^57^ As a result of these large conformational changes, the redox processes display a very large degree of hysteresis;^78^ two-electron reduction of the dicationic **BTX-crown**^**2+**^ occurs at much more cathodic potentials (∼−0.06 V vs Fc/Fc^+^), corresponding to a hysteresis of ∼ 0.87 V (Figures S43–S49). In this reductive step, the neutral **BTX-crown** in the metastable *syn-*folded state is initially formed (Figure 1), which quickly thermally relaxes back to the more stable *anti-*folded state.^57^

Upon cation addition, notable, continuous anodic voltammetric shifts of the oxidation peak potential were observed for most **BTX-crown ethers**, as representatively shown for the titration of **BTX-18c6**_*anti*_ with K^+^ in Figure 5B (see also Section 5.2). This is in good agreement with the behavior of other redox-active crown ethers^48,49,79,80^ and can be rationalized by (electrostatic) destabilization of the higher (i.e., dicationic) oxidation state or by the lower electron density at the BTX core upon binding of the cation. In turn, this is also indicative of significantly weaker binding of the cation to the oxidized receptor state.^11,49^

Depending on the specific cation and **BTX-crown ether**, the maximum magnitude of the voltammetric shift Δ*E*_max_ upon cation binding differed significantly, as shown in Figure S51 and Table S5. For example, the smallest **BTX-15c5***_anti_* displayed only very small voltammetric responses to Na^+^, K^+^, and NH_4_^+^ (Δ*E*_max_ ≈ 10 mV) with very shallow response isotherms, indicative of weak cation binding of **BTX-15c5***_anti_* in the neutral, *anti-*folded state, which is also in good agreement with ^1^H NMR titrations (see Table 1 and Section 5.2). The larger crown ethers displayed much larger voltammetric shifts in almost all cases, the magnitude of which is strongly dependent on the specific cation and host combination. The largest response was obtained for **BTX-21c7***_anti_* in the presence of DBA^+^, with a Δ*E*_max_ of up to 54 mV, while **BTX-24c8***_anti_* responded less significantly to this guest (Δ*E*_max_ = 25 mV, Figure S51B).

The significant change in both the conformation and charge of the receptor upon oxidation is expected to completely turn off cation binding to the **BTX-crown ether**^**2+**^ state, thereby ejecting the initially bound guest. This occurs via both strong Coulombic repulsion and the unfavorable elongation, and thus “unwrapping”, of the crown ether strap in the orthogonal state. Such a conformational switching of guest recognition induced by a redox process is very rare.^81,82^ Indeed, NMR titrations of the isolated **BTX-24c8**^**2+**^**(ClO**_**4**_^**–**^**)**_**2**_ with K^+^ and DBA^+^ confirmed this behavior; in both cases, negligible binding was observed (K < 1 M^–1^), see Figures S33–S35.

Lastly, it should be noted that reduction of the dicationic state does not directly generate the neutral *anti-*folded state but first generates the *syn-*folded state, as discussed in more detail elsewhere.^62,63^ As such, this metastable state can be populated by light irradiation as well as by redox. This was ascertained by UV–vis experiments as shown in Figures 5C and S13. The addition of decamethylferrocene (Fc*) as a reducing agent to **BTX-24c8**^**2+**^ induced immediate, quantitative conversion to **BTX-24c8***_syn_* which thermally relaxed back to **BTX-24c8***_anti_* with a *t*_1/2_ of 85 s, very similar to the relaxation observed following light-induced population of the *syn-*folded state (*vide supra*). Furthermore, the speed of the backward isomerization was again modulated in the same manner by cation binding; the addition of 21.4 mM of either NH_4_^+^ or DBA^+^ led to a speed-up or slowdown of relaxation with *t*_1/2_ of 71 and 127 s, respectively. As discussed above, this is directly reflective of weaker or stronger binding in the *syn-*folded states, respectively. As such, the reduction of the dicationic state is associated with a specific, autonomous sequence of cation binding strength: from no binding → weak binding → strong binding (for NH_4_^+^) or from no binding → strong binding → weak binding (for DBA^+^). This represents, to the best of our knowledge, the first example of such a predefined multistate sequence of guest binding induced by a single redox stimulus.

## Conclusions

Three-state redox- and light-induced conformational switching of an overcrowded alkene receptor is demonstrated for the first time, showing that the inherent geometric and charge modulation of the versatile BTX scaffold can be efficiently transduced to a bound guest in a noncovalent manner. In turn, cation binding modulates both the oxidation potential of the receptor and the rates of thermal relaxation from the *syn-* to the *anti-*folded state. Importantly, all three switching states can be quantitatively and highly reversibly addressed, whereby each state displays notably different cation binding affinities: the nonvolatile, orthogonal dicationic state displays no cation affinity, while the two neutral *anti-* and *syn-*folded states display significant cation affinities. Depending on both the specific crown ether length and the specific cationic guest, either of these neutral folded states displays significantly stronger binding than the other, enabling light-driven switching of cation binding, whereby the metastable, volatile *syn*-folded state is maintained only under constant irradiation. This enables, for example, highly reversible shuttling of the pseudorotaxane-forming DBA^+^ guest between the smaller **BTX-21c7**, whose DBA^+^ affinity strongly diminishes upon irradiation, and the larger **BTX-24c8**, which displays enhanced DBA^+^ binding in the metastable *syn-*folded state. In addition, cation binding can also be switched in a predefined, autonomous three-state sequence (OFF – ON1 – ON2) upon reduction of the dicationic state. As a result, these highly versatile receptive switches are ideal candidates for the construction of multistate, multistimuli-responsive molecular machines, supramolecular host–guest systems, and sensors. In this context, it should also be noted that, while demonstrated here for the switchable recognition of cations, these systems are expected to be easily adaptable for recognition of neutral or anionic guests by incorporation of other receptive motifs.

## Supporting Information Available

Further details on experimental procedures, synthetic procedures, NMR spectra, optical and electrochemical characterization, shuttling performance, and DFT calculations (PDF)
XYZ coordinates of calculated structures (ZIP)
