# Supplementary material for: Multi-State Redox and Light-Driven Switching of Pseudorotaxanation and Cation Shuttling
Source: J Am Chem Soc. 2025 Apr 11;147(16):13649–57. doi: 10.1021/jacs.5c00997 (PMC12023027; doi:10.1021/jacs.5c00997)
Supplement: Supplementary file 1 — ja5c00997_si_001.pdf [file ja5c00997_si_001.pdf]

Supporting Information

Multi-State Redox and Light-driven Switching of Pseudorotaxanation and Cation Shuttling

Robert Hein,[1,2,] Yohan Gisbert[1] and Ben L. Feringa[1,]**

[1]Stratingh Institute for Chemistry, University of Groningen, Nijenborgh 3, 9747AG Groningen, The Netherlands

[2]Present address: Organic Chemistry Institute, University of Münster, Corrensstraße 40, 48149 Münster, Germany

*Corresponding authors: robert.hein@uni-muenster.de, b.l.feringa@rug.nl

1. Experimental 2
2. Synthesis 5
3. UV-Vis Studies 15
4. NMR Studies 18
   4.1 DBA^+ Pseudorotaxanation Studies 18
   4.2 ^1H NMR Titrations 20
   4.3 ^1H NMR Shuttling Experiments 31
   4.4 Improving Shuttling Performance 36
5. Electrochemical Studies 40
   5.1 Voltammetric Characterisation 40
   5.2 Voltammetric Cation Binding Studies 45
6. Computational Studies 49
7. References 50

1. Experimental

General

All chemicals and solvents were obtained from commercial suppliers and used as received. $TBAPF_6$ was of electrochemical grade and obtained from Sigma Aldrich. Unless otherwise noted, HPLC grade solvents were used. In all cases hexafluorophosphate salts of the cations were used. All data analysis and isotherm fitting was carried out with Origin 2018. In all cases 1:1 host-guest stoichiometric binding models were employed.[1] Calculation of the relative host-guest ratio in the ternary ("shuttling") systems was carried out via https://protsim.github.io/protsim as described elsewhere.[2] All photoswitching studies were carried out at 25 °C.

NMR experiments were conducted using a Varian Mercury Plus (400 MHz), an Agilent MR 400 (400 MHz), a Varian Inova 500 (500 MHz) or a Bruker Avance Neo 600 (600 MHz) spectrometer and spectra were referenced to the residual solvent signal. High-resolution mass spectra were recorded on a Thermofisher LTQ Orbitrap XL.

Electrochemical Measurements

All electrochemical experiments were carried out using a three-electrode setup using a PalmSens4 potentiostat. Pt wire was used as a counter electrode and a glassy carbon disk as a working electrode (3 mm diameter) throughout. As reference electrode a non-aqueous $Ag/AgNO_3$ (10 mM $AgNO_3$ in CH_3CN, 100 mM $TBAPF_6$) electrode was used. Prior to each experiment the working electrode surface was polished using a 0.05 µm alumina slurry.

Unless otherwise stated, a scan rate of 100 mV/s was used for all CV experiments and experiments were carried out under ambient conditions. $TBAPF_6$ was used as supporting electrolyte in all cases.

Voltammetric titration experiments were carried out in CH_3CN/DCM 7:3 at a constant BTX-crown ether concentration of 250 µM. This solvent system was chosen to ensure good solubility of the receptors in all switching states as well as the cation salts. The titrant solution contained 100 mM MPF_6 salt (M = K^+, Na^+, NH_4^+ or DBA^+) such that the overall ionic strength was always kept constant at 100 mM. The first CV scan was used to determine the shift in oxidation potential upon cation addition.

UV-Vis Measurements

UV-Vis spectra were recorded on an Agilent Cary 8454 spectrophotometer using 1 x 1 cm quartz cuvettes. All experiments were carried out in CH_3CN/DCM 7:3 at a constant BTX-crown ether concentration of 50 µM in a rapidly stirred solution at 25 °C (initially 2 mL solution). The cuvette was irradiated with a Thorlabs LED (M365, controlled with a Thorlabs LEDD1B driver

at highest intensity) at a 90° angle which enabled quantitative switching to the *syn*-folded state in a few seconds. After turning off the light, relaxation was followed by recording spectra with interval times between 4 and 20 s, depending on the relaxation speed. This was repeated at various cation concentrations, which was achieved by addition of aliquots of the cation salt (50 mM in CH_3CN/DCM 7:3, containing 50 µM of the BTX-crown ether).

^{1}H NMR Irradiation (Titration) Studies

NMR irradiation experiments were carried out using a Varian Inova 500 spectrometer. The sample was irradiated *in situ* with 365 nm light using a Thorlabs LED (M365, controlled with a Thorlabs LEDD1B driver, at half intensity, this corresponds to a power of ~50 mW) with a 1500 µm optical fiber (FT1500UMT) to guide the light directly into the NMR tube. All NMR titrations were carried out in CD_3CN/CD_2Cl_2 7:3 at an initial BTX-crown ether concentration of 1 mM at 25 °C. For titrations without any *in situ* irradiation (*i.e.* on the dicationic **BTX-24c8^{2+}**) an initial host volume of 500 µL was used, into which a 50 mM solution of the cation was titrated.

For titrations with *in situ* irradiation (*i.e.* to determine binding constants to both the *anti*- and *syn*-folded states) an initial host volume of 400 µL was used, into which a 40 mM solution of the cation was titrated (in CD_3CN/CD_2Cl_2 7:3). First, the ^{1}H NMR spectrum of the native, *anti*-folded receptor was recorded. The sample was then irradiated *via* the optical fiber until quantitative switching to the syn-folded state was achieved (typically ~1 min) after which its ^{1}H NMR spectrum was recorded under continuous irradiation. This procedure was repeated at various cation concentrations by letting the sample relax to the *anti*-folded state, removing the fiber, adding aliquots of the cation solution and re-inserting the fiber into the NMR tube. In this manner, the binding constants to both folded states were determined from one experiment on the same sample. Under fully equilibrated conditions (*i.e.* for the pure *anti/syn*-folded states) 16 scans were recorded. For all titrations with **BTX-18c6**, **BTX-21c7** and **BTX-24c8** the following host/cation stoichiometries were measured: 0, 0.5, 1, 1.5, 2, 3, 5, 7 and 10 equivalents. For the titration of **BTX-15c5**, which relaxes much slower, especially in the presence of cations, the following host/cation stoichiometries were measured: 0, 1, 2, 3, 5, 7 and 10 equivalents.

For relaxation/shuttling studies (Figure 4B), ^{1}H NMR spectra were continually recorded, whereby each data point contains 2 scans, corresponding to a temporal resolution of ~8 s.

Computational methods

For every studied structure (*syn*- and *anti*-folded isomers of **BTX-21c7** and **BTX-24c8**, with and without a dibenzylammonium (DBA^+) guest), ensembles of conformers were generated using the CREST[3-5] (Conformer–Rotamer Ensemble Sampling Tool) software at the GFN2-xTB(GBSA)[6]

level of theory using acetonitrile as the solvent. An energy threshold of 15-20 kcal/mol was used, leading to ensembles composed of 4057 to 14222 conformers. For the cationic host-guest complexes, the counter anion was not considered. For the host-guest complexes, the NCI (non-covalent interaction) mode of CREST was used. For the *syn* isomers, a constrain was applied on the S-S distance (distance = 4.3 Å, force constant = 0.001).

These ensembles of conformers were further refined using the CENSO[7] software (Commandline Energetic Sorting) following a three-step workflow involving a cheap pre-screening with an energy threshold of 4 kcal/mol at the B97-D3/def2-SV(P) level of theory, using the Orca 5.0.4[8] software. Then, a more accurate energy calculation was performed at the r^2SCAN-3c/SMD[acetonitrile]/GmRRHO(GFN2[alpb]-bhess) level of theory, using Orca for calculating the single point energy while the thermostatistical contributions (G_mRRHO) were calculated using the XTB software. Following this step, conformers within a 3.5 kcal/mol energy difference from the lowest lying one were conserved for the next step. Finally, the geometry of all remaining isomers (ensembles of 21 to 115 conformers) was optimized with the composite method r^2SCAN-3c,[9] using the Solvation Model Density SMD(Acetonitrile) solvent model[10] with Orca. The thermochemical data was calculated at 25 °C. Single-point energy was calculated at this level of theory, while G_mRRHO was calculated at the GFN2[alpb]-bhess level of theory.

2. Synthesis

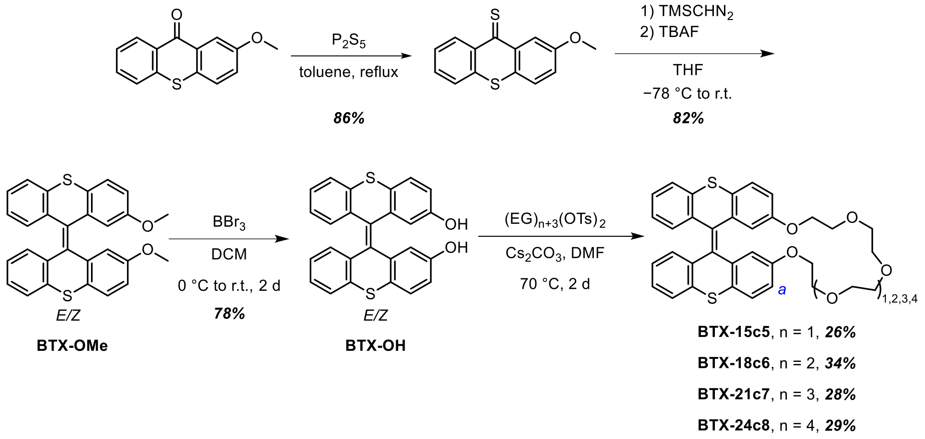

Scheme S1. Synthesis of BTX crown ether receptors.

Note that the naming system of the crown ether derivatives described herein deviates somewhat from the established way where all atoms that are part of the overall macrocyclic crown ether scaffold are counted. Specifically, we disregard the atoms of the BTX core in the fjord region, as this area is so sterically crowded that it does not contribute to the overall size of the macrocycle. These crown ethers are thus technically 6 atoms larger but more closely resemble their parent, non-extended analogues.

BTX-OMe was synthesized according to literature procedures.[11]

BTX-OH

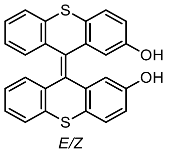

BTX-OMe (2.3 g, 5.08 mmol, 1 equiv.) was dissolved in 200 mL anhydrous DCM under N_2. The solution was cooled to 0 °C and 40 mL BBr_3 (1 M in DCM, 40.7 mmol, 8 equiv.) was slowly added. The solution was allowed to warm to room temperature under stirring and reacted for 2 d, after which the reaction was carefully quenched by addition of ~10 mL saturated aqueous NH_4Cl. The majority of the DCM was then removed in vacuo. 250 mL water were added and the product extracted with EtOAc (3 x 250 mL). The combined organic phases were then washed with water (3 x 150 mL), dried over $MgSO_4$ and concentrated in vacuo. Further purification by silica gel chromatography (DCM to DCM/MeOH 9:1 gradient) then afforded 1.671 g (78%) of BTX-OH as a light-brown solid as a mixture of *E/Z* isomers (60:40 ratio major/minor isomer), which was stored in the freezer.

^{1}H NMR (400 MHz, DMSO) δ 9.33 (s, 2H, *major*), 9.26 (s, 2H, *minor*), 7.67 – 7.53 (t, 2H *major* + 2H *minor*), 7.41 (t, 2H *major* + 2H *minor*), 7.20 (m, 2H *major* + 2H *minor*), 7.02 (td, *J* = 7.5, 1.3 Hz, 2H, *minor*), 6.93 (td, *J* = 7.6, 1.3 Hz, 2H, *major*), 6.76 (dd, *J* = 7.8, 1.4 Hz, 2H, *minor*), 6.71 – 6.56 (m, 4H *major* + 2H *minor*), 6.27 (d, *J* = 2.6 Hz, 2H, *major*), 6.12 (d, *J* = 2.6 Hz, 2H, *minor*).

As a result of its low solubility no ^{13}C NMR spectra of this compound were recorded.

HR-MS (ESI-): Calculated for $C_{26}H_{15}O_2S_2$ $[M-H]^-$: 423.0519. Found: 423.0518

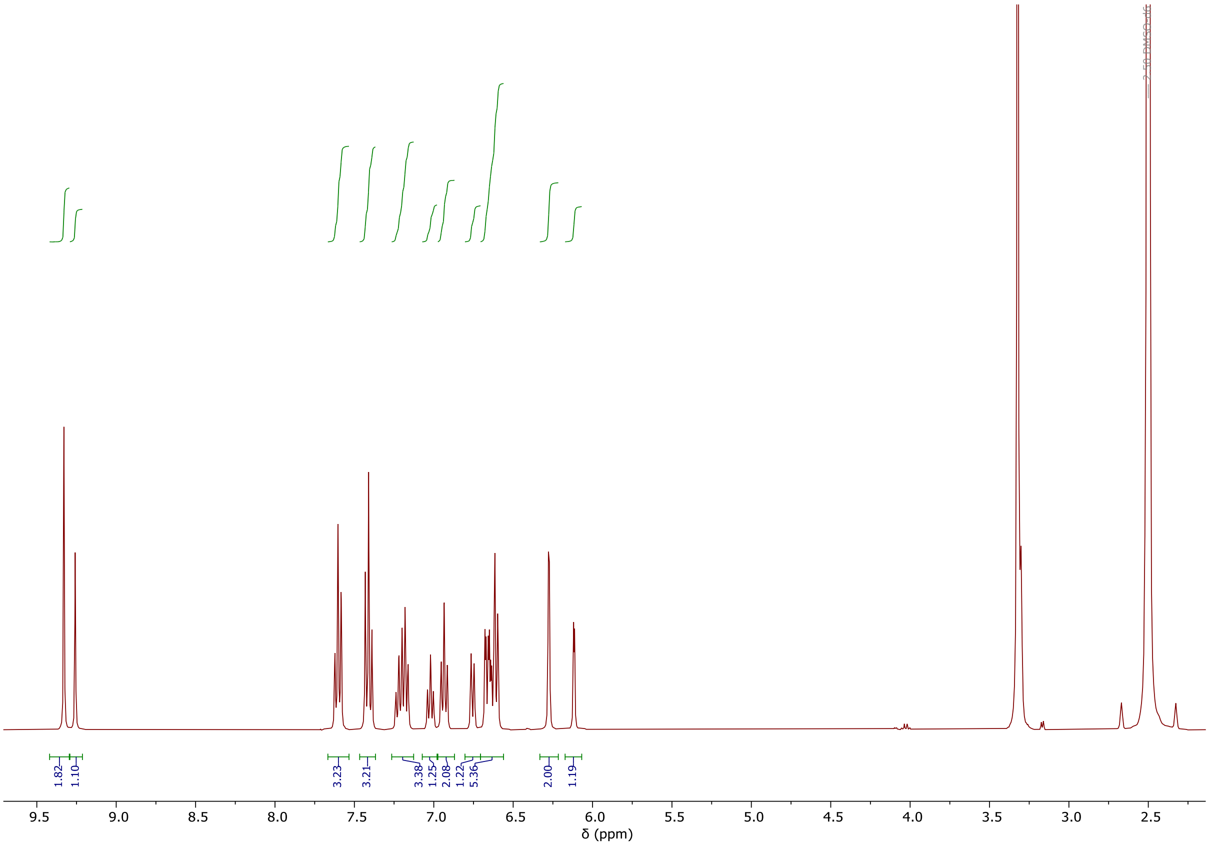

Figure S1. ^{1}H NMR (400 MHz, DMSO) spectrum of BTX-OH as a E/Z mixture (60:40 ratio major/minor isomer).

General Procedure for Synthesis of BTX-crown ethers

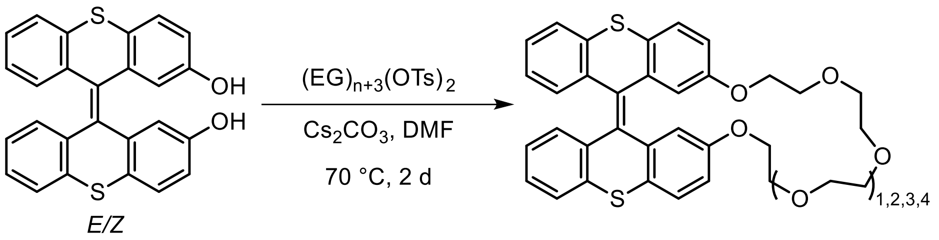

BTX-OH (1 equiv., as *E/Z* isomer mixture), Cs_2CO_3 (3 equiv.) and the appropriate ethyleneglycol ditosylate (1.05 or 1.1 equiv.) were weighed into a thoroughly dried Schlenk flask. After three

vacuum/N_2 cycles, anhydrous DMF was adder under N_2 to give a 4 mM solution (wrt. BTX-OH), which was then heated to 70 °C for 2 d. The solvent was then removed in vacuo and the crude re-dissolved in DCM (~300 – 500 mL, depending on scale). The organic phase was then washed with water (3x 300 – 500 mL), dried over $MgSO_4$ and concentrated in vacuo. Purification was carried out *via* (multiple) silica and/or neutral alumina column chromatography using DCM/MeOH (0-5% gradients). All BTX-crown ethers were obtained as off-white solids.

BTX-15c5

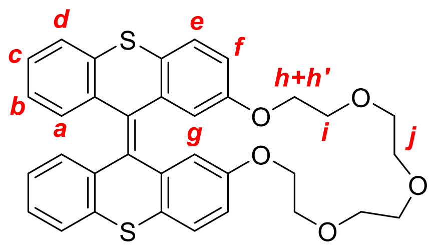

Obtained *via* general procedure for synthesis of BTX-crown ethers from 80 mg BTX-OH and 104 mg tetraethylene glycol di(*p*-toluenesulfonate)[12] (1.1 equiv.) affording 28 mg BTX-15c5 (26%) after purification.

^{1}H NMR (600 MHz, $CDCl_3$) δ 7.53 (d, *J* = 7.8 Hz, 2H, H_d), 7.43 (d, *J* = 8.5 Hz, 2H, H_e), 7.16 – 7.08 (m, 2H, H_c), 6.96 – 6.85 (m, 2H, H_b), 6.85 – 6.76 (m, 4H, $H_f + H_a$), 6.42 (d, *J* = 2.7 Hz, 2H, H_g), 3.81 (ddd, *J* = 10.4, 5.1, 2.9 Hz, 2H, $H_{h/h'}$), 3.78 – 3.61 (m, 12H, H_{i-j}), 3.54 (ddd, *J* = 10.0, 6.6, 3.1 Hz, 2H, $H_{h/h'}$).

^{13}C NMR (151 MHz, $CDCl_3$) δ 157.4, 137.0, 136.1, 135.9, 133.8, 130.0, 128.3, 127.2, 127.2, 126.9, 125.8, 116.1, 115.0, 71.1, 71.0, 69.4, 68.3.

HR-MS (ESI+): Calculated for $C_{34}H_{30}O_5S_2Na$ $[M + Na]^+$: 605.14269. Found: 605.14262

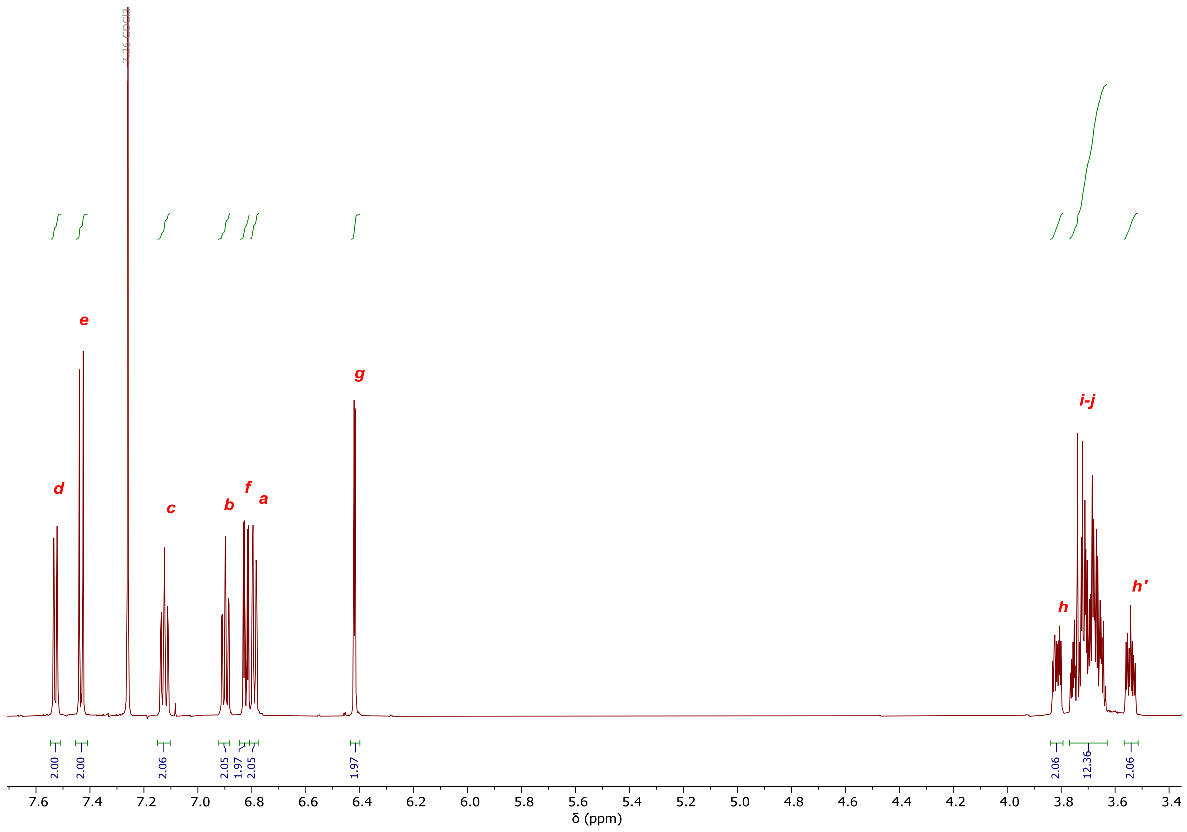

Figure S2. ^{1}H NMR (600 MHz, $CDCl_3$) of **BTX-15c5**.

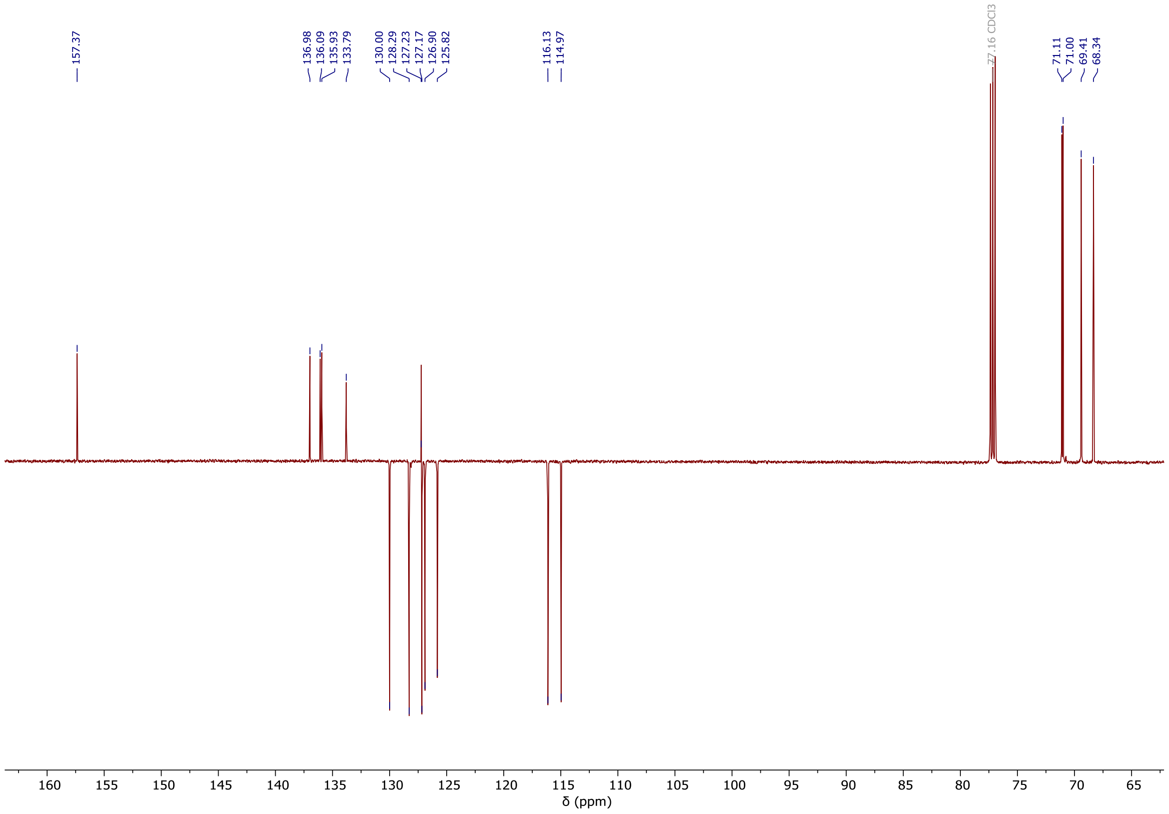

Figure S3. ^{13}C NMR (151 MHz, APT, $CDCl_3$) of **BTX-15c5**.

BTX-18c6

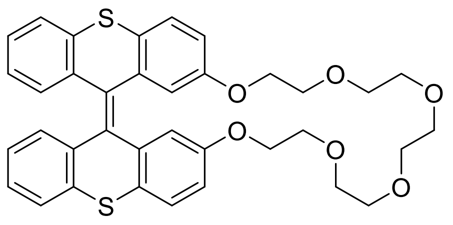

Obtained *via* general procedure for synthesis of BTX-crown ethers from 100 mg BTX-OH and 142 mg pentaethylene glycol di(*p*-toluenesulfonate) (1.1 equiv.) affording 50 mg BTX-18c6 (34%) after purification.

^{1}H NMR (600 MHz, $CDCl_3$) δ 7.53 (dd, *J* = 7.8, 1.2 Hz, 2H), 7.42 (d, *J* = 8.5 Hz, 2H), 7.12 (td, *J* = 7.5, 1.4 Hz, 2H), 6.90 (td, *J* = 7.5, 1.2 Hz, 2H), 6.80 (td, *J* = 8.6, 2.0 Hz, 4H), 6.40 (d, *J* = 2.7 Hz, 2H), 3.78 (ddd, *J* = 10.1, 5.6, 2.9 Hz, 2H), 3.75 – 3.62 (m, 16H), 3.51 (ddd, *J* = 9.6, 6.4, 2.8 Hz, 2H).

^{13}C NMR (151 MHz, $CDCl_3$) δ 157.4, 136.9, 136.1, 135.9, 133.8, 130.0, 128.3, 127.2, 127.2, 126.9, 125.8, 116.3, 114.8, 71.1, 70.8, 70.7, 69.5, 68.0.

HR-MS (ESI+): Calculated for $C_{36}H_{35}O_6S_2$ $[M + H]^+$: 627.18696. Found: 627.18620

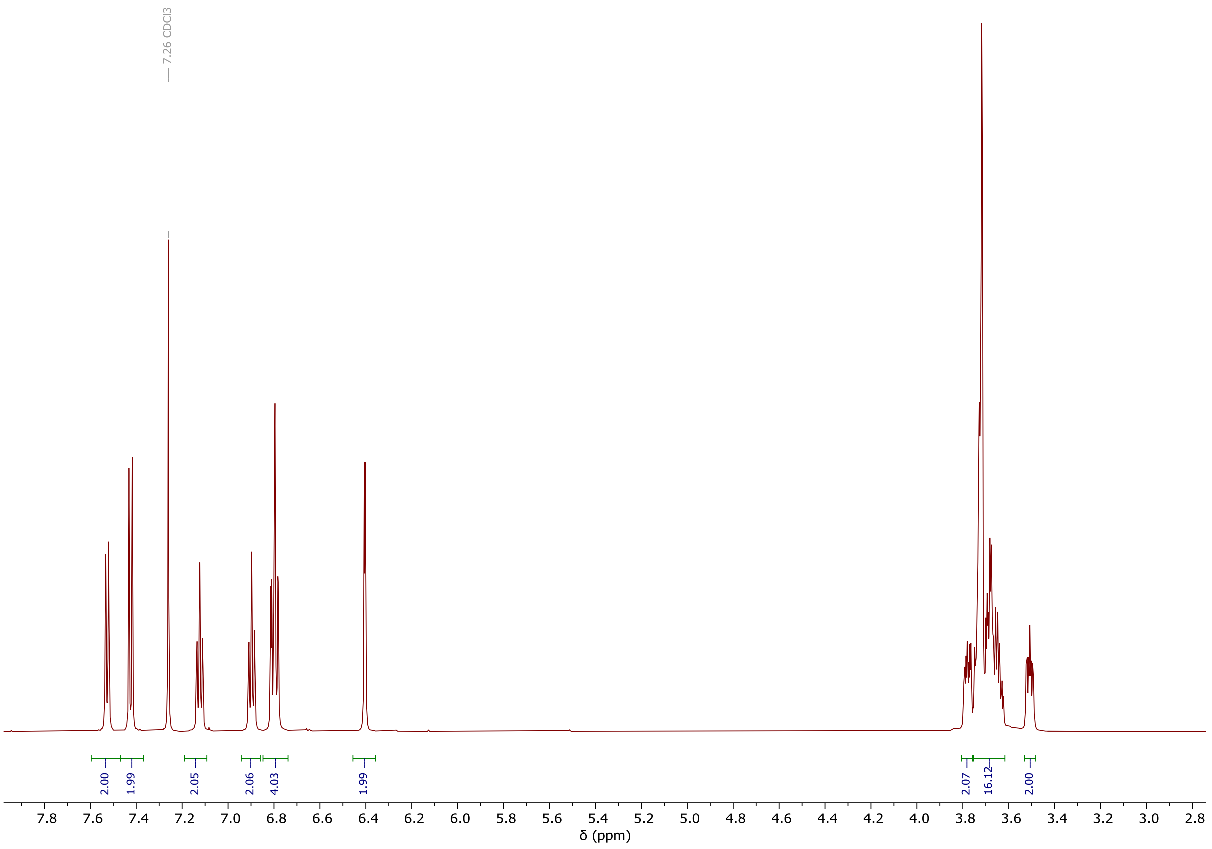

Figure S4. ^{1}H NMR (600 MHz, $CDCl_3$) of **BTX-18c6**. The proton assignments are identical as for BTX-15c5, see Figure S2.

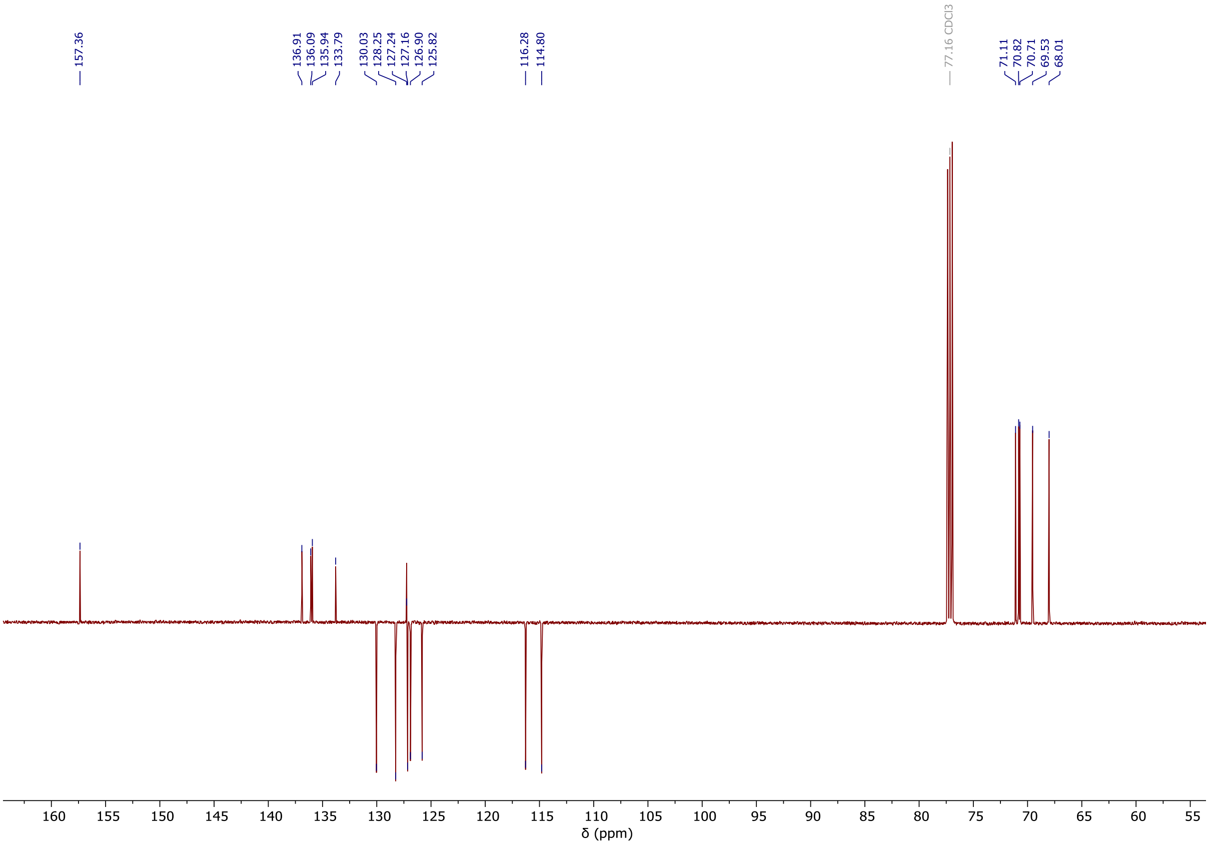

Figure S5. ^{13}C NMR (151 MHz, APT, $CDCl_3$) of **BTX-18c6**.

BTX-21c7

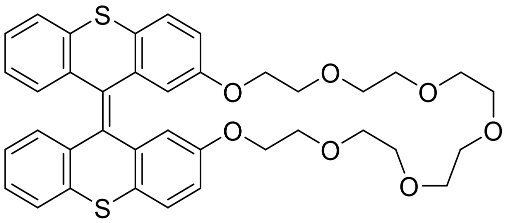

Obtained *via* general procedure for synthesis of BTX-crown ethers from 185 mg BTX-OH and 270 mg hexaethylene glycol di(*p*-toluenesulfonate) (1.05 equiv.) affording 81 mg BTX-21c7 (28%) after purification.

^1H NMR (600 MHz, $CDCl_3$) δ 7.53 (dd, *J* = 7.8, 1.2 Hz, 2H), 7.42 (d, *J* = 8.6 Hz, 2H), 7.12 (td, *J* = 7.5, 1.4 Hz, 2H), 6.90 (td, *J* = 7.5, 1.2 Hz, 2H), 6.82 – 6.76 (m, 4H), 6.40 (d, *J* = 2.7 Hz, 2H), 3.80 (ddd, *J* = 10.3, 5.7, 3.3 Hz, 2H), 3.76 – 3.61 (m, 20H), 3.57 (ddd, *J* = 9.8, 6.2, 3.3 Hz, 2H).

^{13}C NMR (151 MHz, $CDCl_3$) δ 157.3, 137.0, 136.1, 135.9, 133.8, 123.0, 128.2, 127.3, 127.2, 126.9, 125.8, 116.0, 115.2, 71.1 (2C), 70.9 (2C), 69.5, 68.0.

HR-MS (ESI+): Calculated for $C_{38}H_{38}O_7S_2Na$ $[M + Na]^+$: 693.19512. Found: 693.19504

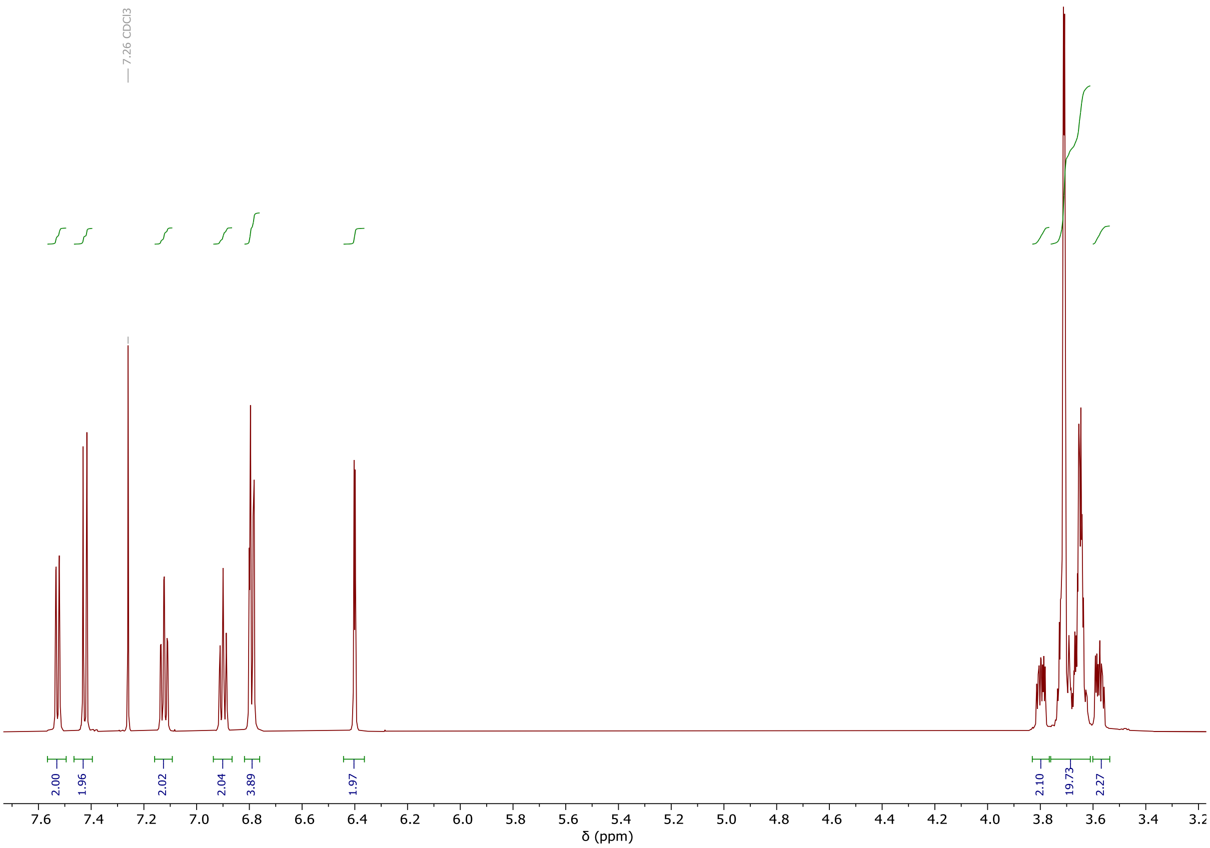

Figure S6. ^{1}H NMR (600 MHz, $CDCl_3$) of **BTX-21c7**. The proton assignments are identical as for BTX-15c5, see Figure S2.

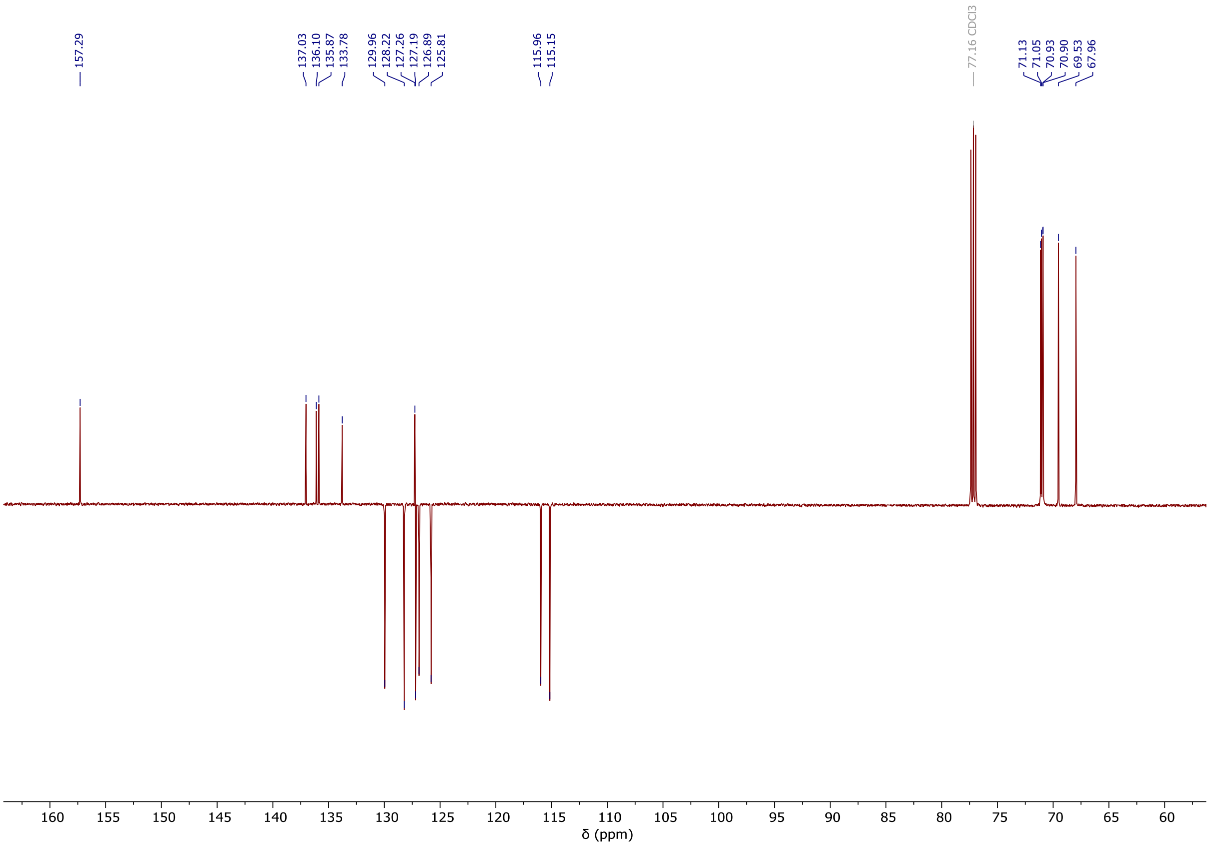

Figure S7. ^{13}C NMR (151 MHz, APT, $CDCl_3$) of **BTX-21c7**.

BTX-24c8

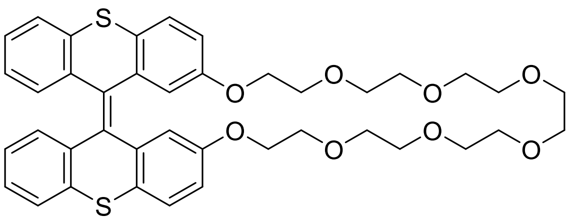

Obtained *via* general procedure for synthesis of BTX-crown ethers from 177 mg BTX-OH and 277 mg heptaethylene glycol di(*p*-toluenesulfonate)[12] (1.05 equiv.) affording 85 mg BTX-24c8 (29%) after purification.

^{1}H NMR (600 MHz, $CDCl_3$) δ 7.53 (dd, *J* = 7.8, 1.2 Hz, 2H), 7.42 (d, *J* = 8.5 Hz, 2H), 7.12 (td, *J* = 7.5, 1.4 Hz, 2H), 6.90 (td, *J* = 7.5, 1.2 Hz, 2H), 6.82 – 6.76 (m, 4H), 6.40 (d, *J* = 2.7 Hz, 2H), 3.85 – 3.78 (m, 2H), 3.73 – 3.57 (m, 26H).

^{13}C NMR (151 MHz, $CDCl_3$) δ 157.3, 137.1, 136.1, 135.9, 133.8, 123.0, 128.2, 127.2, 127.2, 126.9, 125.8, 115.8, 115.2, 71.0 (3C), 70.9 (2C), 69.5, 67.8.

HR-MS (ESI+): Calculated for $C_{40}H_{42}O_8S_2Na$ $[M + Na]^+$: 737.22134. Found: 737.21944

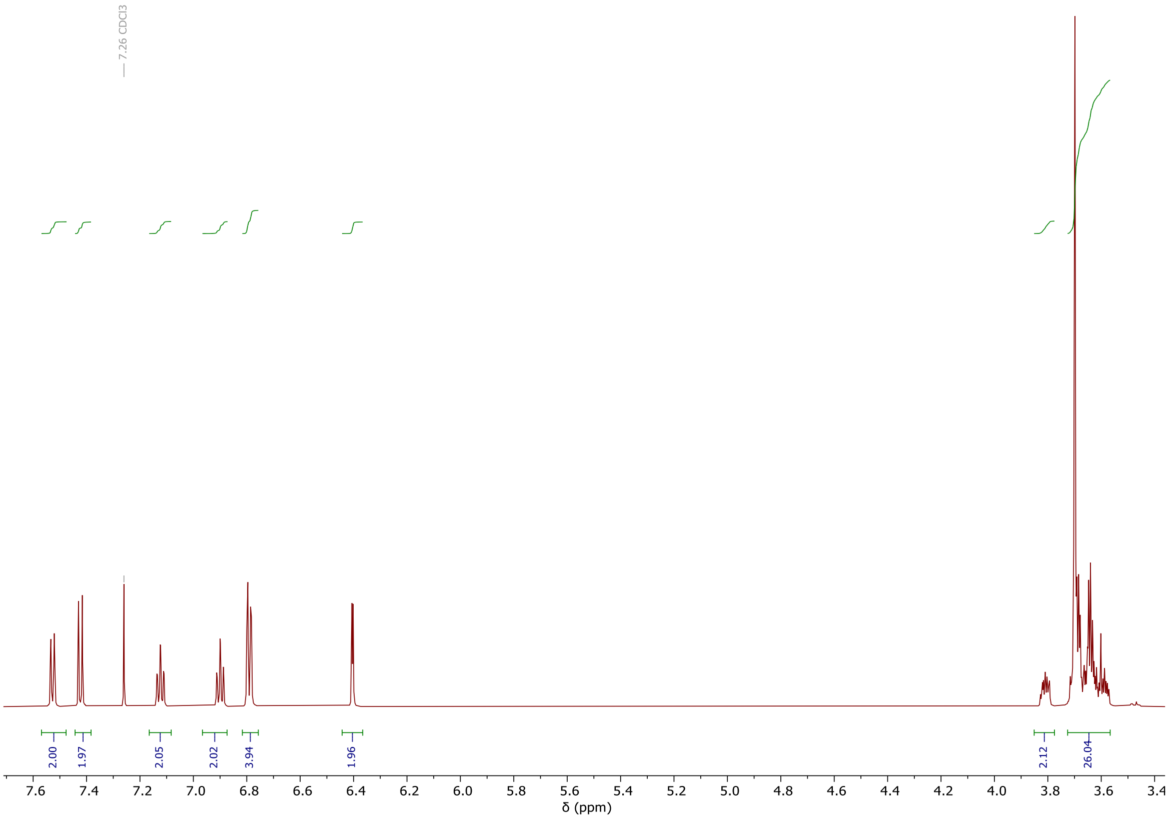

Figure S8. ^{1}H NMR (600 MHz, $CDCl_3$) of **BTX-24c8**. The proton assignments are identical as for BTX-15c5, see Figure S2.

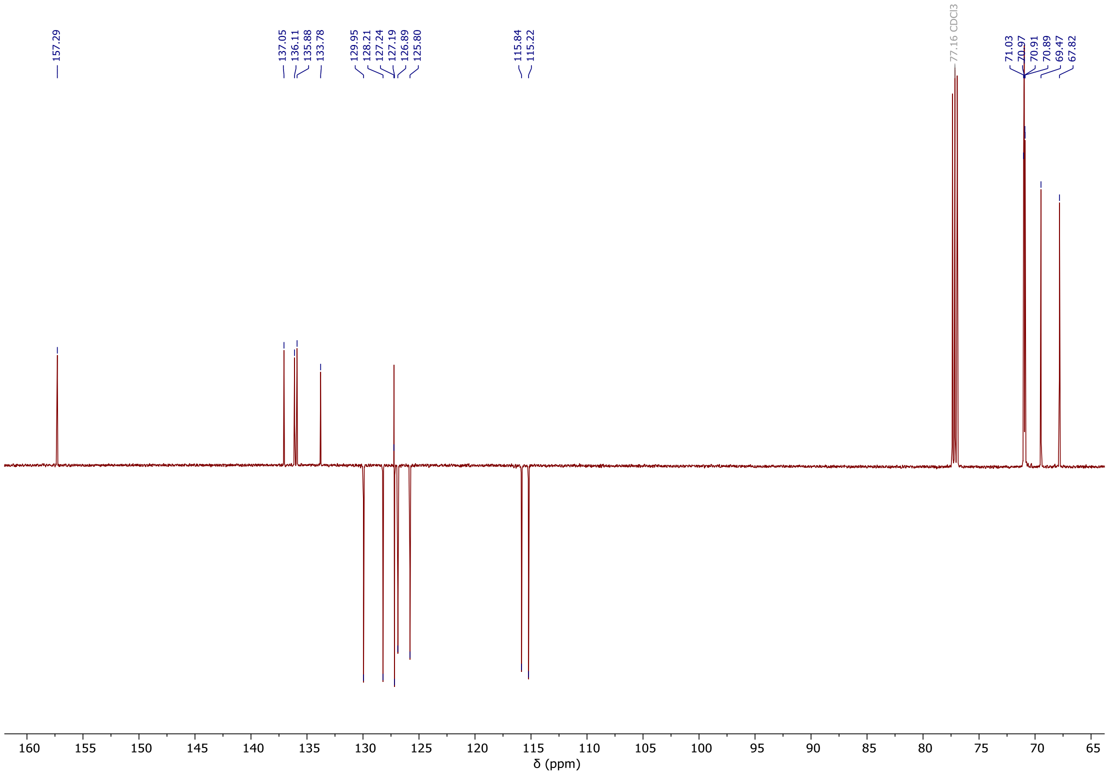

Figure S9. ^{13}C NMR (151 MHz, APT, $CDCl_3$) of **BTX-24c8**.

BTX-24c8^{2+}(ClO_4^-)$_2$

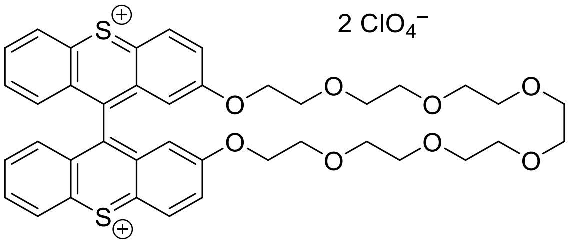

2.8 mg BTX-24c8 (0.004 mmol, 1 equiv.) was dissolved in 1.5 mL DCM and 0.5 mL CH_3CN. To this was added a spatula of $Fe(ClO_4)_3$•xH_2O (excess) and the mixture was sonicated for 5 min. The organic phase was then washed with 2 x 1 mL water, dried over $MgSO_4$ and concentrated in vacuo, affording 4.1 mg (115%) of BTX-24c8^{2+}(ClO_4^-)$_2$ as a dark red/purple solid.

^{1}H NMR (400 MHz, CD_3CN/CD_2Cl_2 7:3) δ 9.00 (d, *J* = 9.4 Hz, 2H), 8.96 (d, *J* = 8.6 Hz, 2H), 8.33 (ddd, *J* = 8.4, 6.9, 1.1 Hz, 2H), 8.18 (dd, *J* = 9.4, 2.5 Hz, 2H), 7.80 (ddd, *J* = 9.0, 6.9, 1.1 Hz, 2H), 7.52 (d, *J* = 8.8 Hz, 2H), 6.79 (d, *J* = 2.5 Hz, 2H), 3.90 (ddd, *J* = 10.5, 7.4, 2.7 Hz, 2H), 3.82 (ddd, *J* = 11.1, 4.7, 2.7 Hz, 2H), 3.65 (ddd, *J* = 11.6, 7.4, 2.6 Hz, 2H), 3.57 – 3.38 (m, 22H).

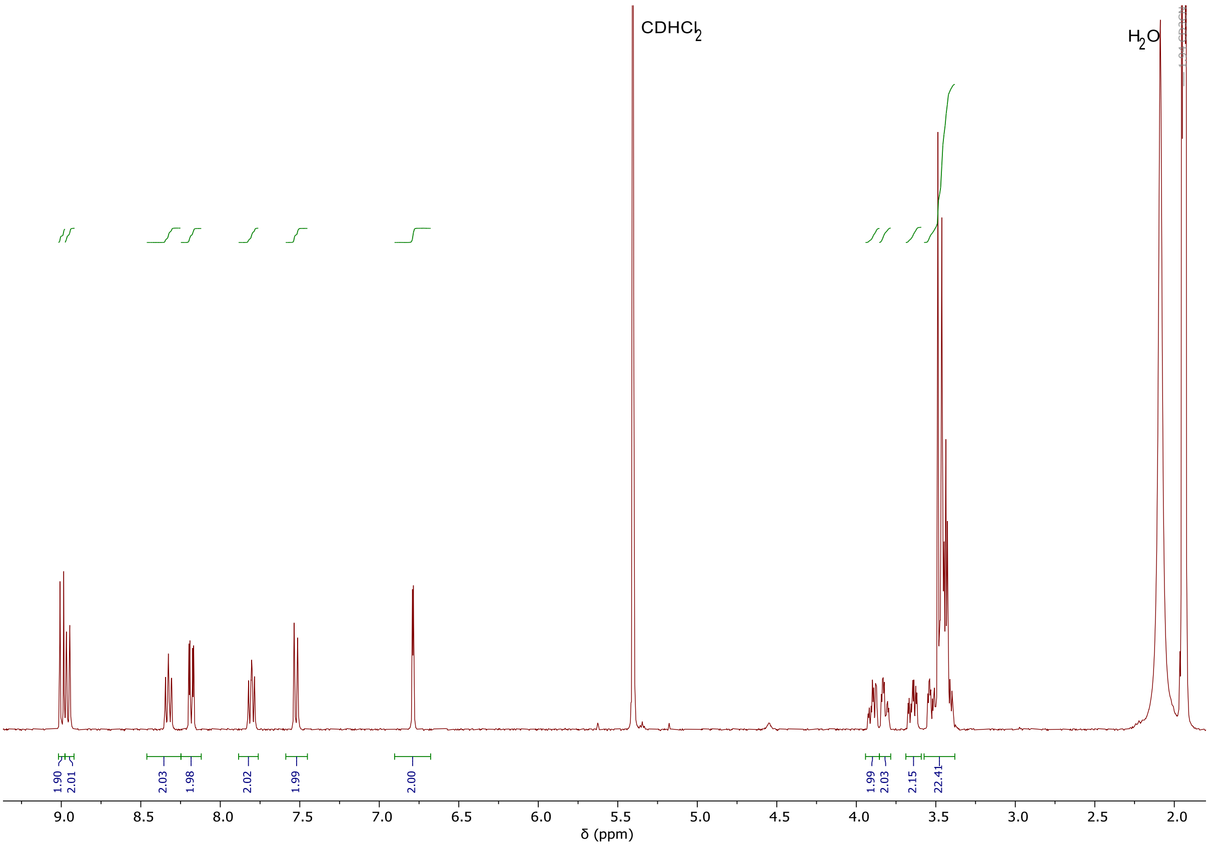

Figure S10. ^{1}H NMR (400 MHz, CD_3CN/CD_2Cl_2 7:3) of **BTX-24c8^{2+}(ClO_4^-)$_2$**.

DBA$^+$PF$_6^-$

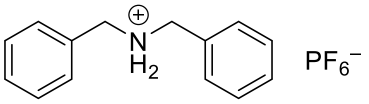

This salt was prepared by an adapted literature procedure:[13]

Dibenzylamine (1 g, 5.07 mmol, 1 equiv.) was suspended in 20 mL water under strong stirring. To this mixture 0.985 mL aqueous HPF_6 (55 wt%, 6.134 mmol, 1.21 equiv.) was added dropwise. This immediately led to formation of a white precipitate which was filtered and washed with copious amounts of water, affording 1.16 g (67%) of DBA$^+$PF$_6^-$. The ^{1}H NMR spectrum of this compound is in good agreement with literature.[14]

3. UV-Vis Studies

As shown in Figure 2A, the absorbance changes observed during interconversion between the *anti*- and *syn*-folded states proceed *via* the same, well-defined isosbestic points (in both the light-driven forward, as well as the thermally-driven backward isomerization), confirming mono-molecular processes without intermediates.

Figure S11 representatively shows the change in absorbance at 365 nm of the crown ether host (here **BTX-18c6**) upon light-induced switching to the *syn*-folded state and subsequent thermal relaxation upon titration of increasing cation concentrations (here NH_4^+). From this "kinetic" titration the relaxation $t_{1/2}$ at different cation concentrations was determined by fitting to a simple exponential decay function. The corresponding data for the titrations of **BTX-18c6** with the other tested cations as well as for **BTX-21c7** and **BTX-24c8** are qualitatively similar. The plots of $t_{1/2}$ as a function of cation concentration for all titrations of **BTX-18c6**, **BTX-21c7** and **BTX-24c8** are shown in Figure 2D-F.

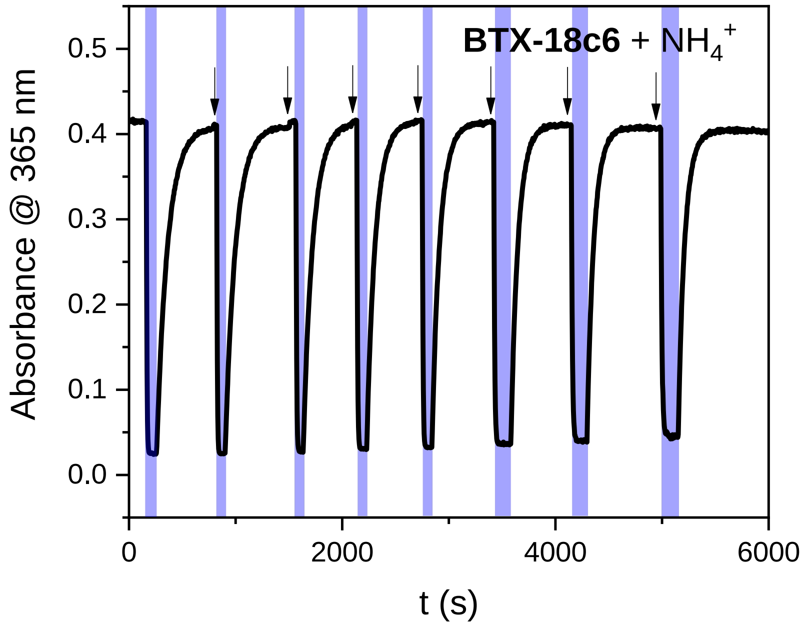

Figure S11. Changes in absorbance (at 365 nm) of 50 µM **BTX-18c6** in CH_3CN/CH_2Cl_2 7:3 upon light-driven and thermal switching between *anti*- and *syn*-folded states in the presence of increasing concentrations of NH_4^+. The blue shaded areas correspond to irradiation with 365 nm light, inducing quantitative switching to the *syn*-folded state. Upon turning off the light, thermal relaxation to the *anti*-folded state occurs, the speed of which is dependent on cation concentration. The black arrows denote where aliquots of a NH_4PF_6 stock solution (in the same solvent, and also containing 50 µM of the host) were added, following which the switching cycle was repeated, enabling quantification of $t_{1/2}$ at different cation concentrations.

These isotherms were fitted to a 1:1 host-guest stoichiometric binding model,[1] from which the $t_{1/2}$ of the fully bound host-guest complex was determined (by extrapolation to $[C^+] = \infty$), see Table S1). This fitting also afforded binding constants, however it should be noted that these are somewhat meaningless as they do not represent binding to either the *anti*- or *syn*-

folded states alone, but correspond to an apparent binding constant that lies in between that of both states. As such, these apparent binding constants are not discussed further.

For **BTX-15c5** no full titrations of this type were carried out, as its relaxation is prohibitively slow. Instead, only one data point at high cation concentration (50 mM) was measured (Table S1).

Table S1. Thermal half-life times $t_{1/2}$ (s) of the *syn*-folded states of BTX-crowns in CH_3CN/CH_2Cl_2 7:3 in the presence and absence of cations.

	**BTX-15c5**[a]	**BTX-18c6**[b]	**BTX-21c7**[b]	**BTX-24c8**[b]
**Free**	180	72	71	74
**+ K^+**	>3030	32	56	62
**+ Na^+**	>710	27	54	76
**+ NH_4^+**	>1230	39	52	61
**+ DBA^+**	/	/	36	143

a – in the presence of 50 mM cation. b – obtained from data fitting (extrapolated to $[C^+] = \infty$).

The strong correlation between K_{syn}/K_{anti} binding preferences and the observed changes in thermal back isomerization upon addition of cations can be rationalized by the Bell–Evans–Polanyi principle, which states that in some sets of closely related systems there is a linear relationship between the activation energy E_a and the overall enthalpy of the reaction.[15] More specifically, a more exothermic reaction will lead to a lowering of E_a. Here, the *syn* → *anti* relaxation is indeed more exothermic if K_{anti} is larger than K_{syn}, thereby leading to a decrease of E_a and thus lifetime ($t_{1/2}$) of the *syn*-folded state (Figure S12). In contrast, E_a (and $t_{1/2}$) increase when $K_{syn} > K_{anti}$. As a result, the change in relaxation kinetics upon cation addition (Figure 2C-F and Table S1) is directly reflective of the relative difference in cation binding to the folded states and can thus be utilized as a convenient assessment thereof. As for virtually all combinations of cations and **BTX-crowns** a significant change in relaxation kinetics is observed, we can infer that binding strength is significantly altered upon light-induced conformational switching in all cases. This leads to either release or take-up of the cation upon irradiation to the *syn*-folded state.

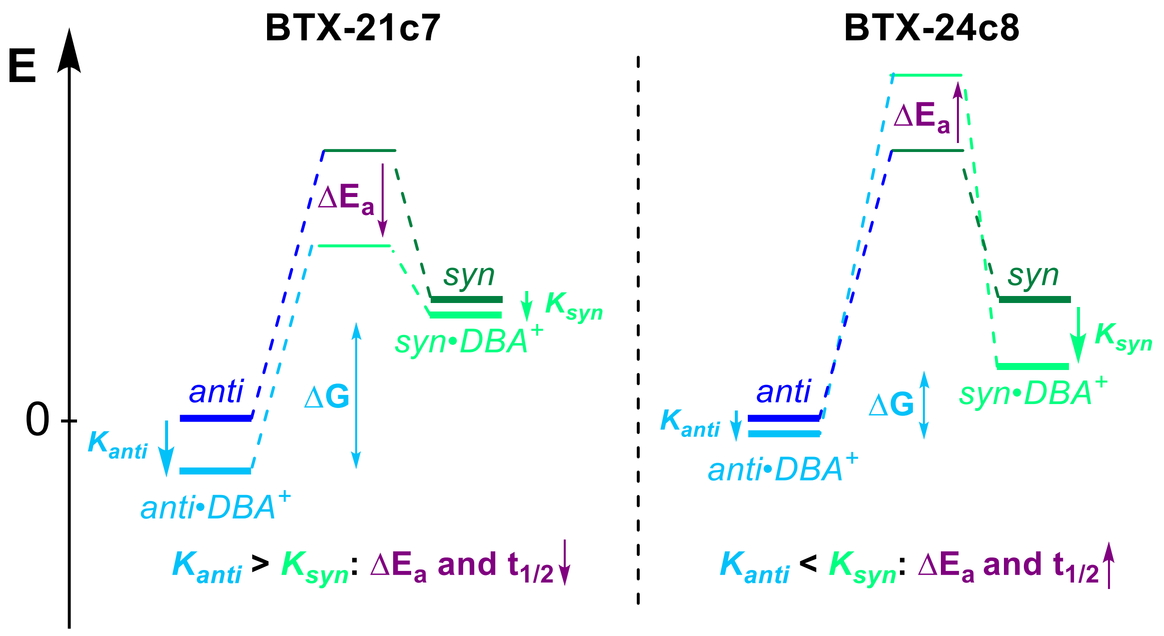

Figure S12. Schematic energy landscape for the light-driven switching of **BTX-21c7** and **BTX-24c8** in the presence and absence of a guest such as DBA^+. According to the Bell–Evans–Polanyi principle the activation energy decreases if cation binding is stronger in the *anti* than the *syn*-folded state, thereby speeding up the thermal relaxation to the *anti*-folded state (and *vice versa*).

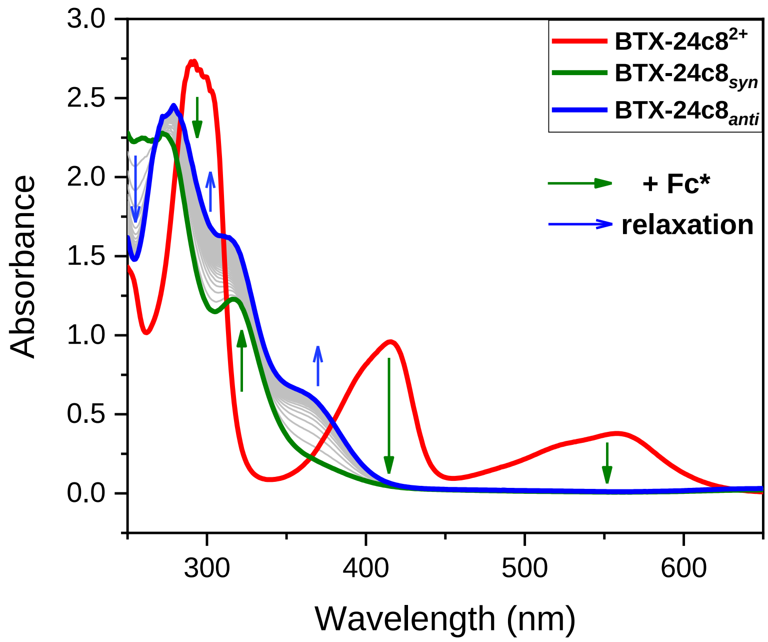

Figure S13. UV-vis spectral changes of 50 µM **BTX-24c8^{2+}(ClO_4^-)$_2$** in CH_3CN/CH_2Cl_2 7:3 upon addition of 3 equiv. of decamethyl ferrocene (Fc*) which induces immediate and quantitative reduction to **BTX-24c8$_{syn}$** (green arrows) which then relaxes to **BTX-24c8$_{anti}$** (blue arrows). The corresponding time traces are shown in Figure 5C.

4. NMR Studies

4.1 DBA$^+$ Pseudorotaxanation Studies

To investigate the ability of the different crown ethers to form pseudorotaxanes with DBA$^+$ ^{1}H NMR studies were conducted in CD_2Cl_2. As shown in Figure S14, addition of 1 equiv. of DBA$^+$ to a 2 mM solution of **BTX-18c6**$_{anti}$ induced no notable changes in the proton chemical shifts of the receptor, indicating no interaction between the host and DBA$^+$. In contrast, for both **BTX-21c7**$_{anti}$ and **BTX-24c8**$_{anti}$ notable shifts of the BTX core protons, as well as strong splitting of the crown ether protons, were observed (Figures S15-16), indicative of formation of pseudorotaxanes. These findings are in good agreement with the literature, where (pseudo)rotaxane formation of DBA$^+$ with crown ethers of various size was observed.[16]

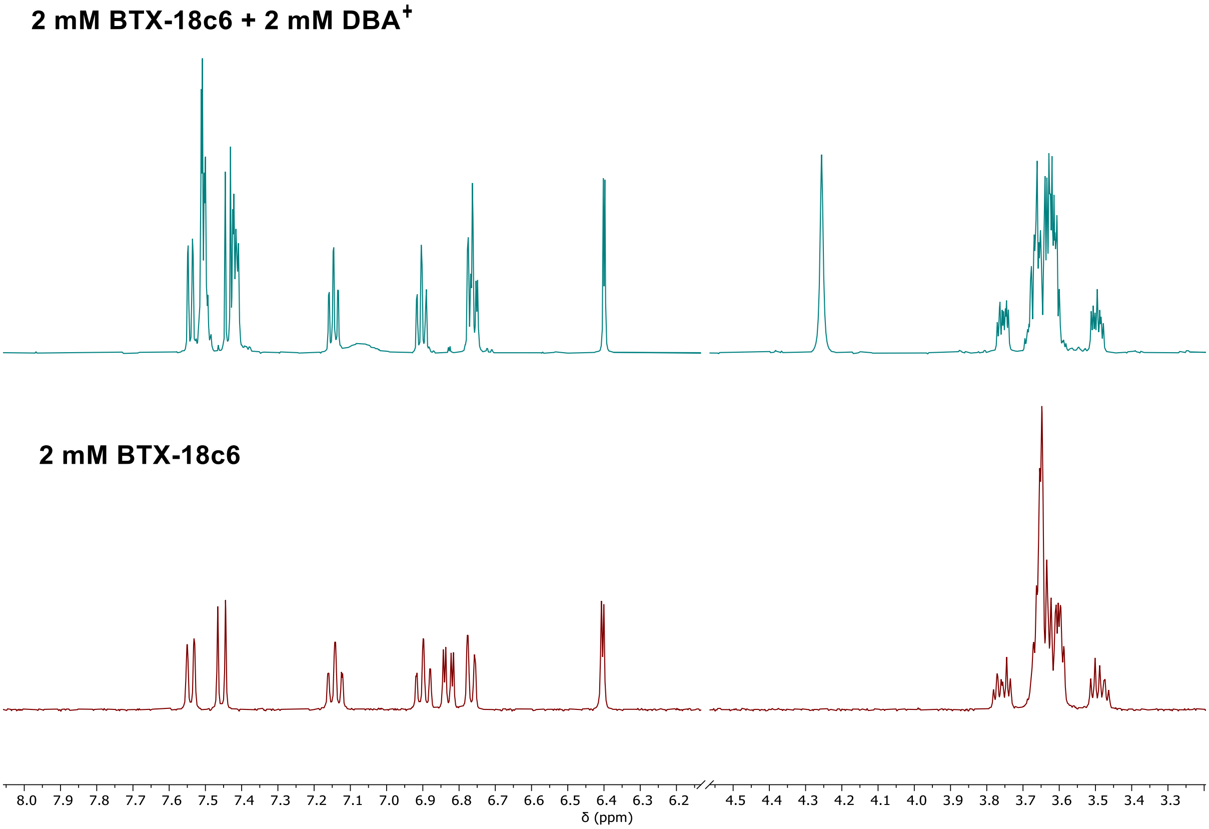

Figure S14. Stacked ^{1}H NMR spectra of 2 mM **BTX-18c6**$_{anti}$ in CD_2Cl_2 in the presence (top) and absence (bottom) of 1 equiv. DBA$^+$.

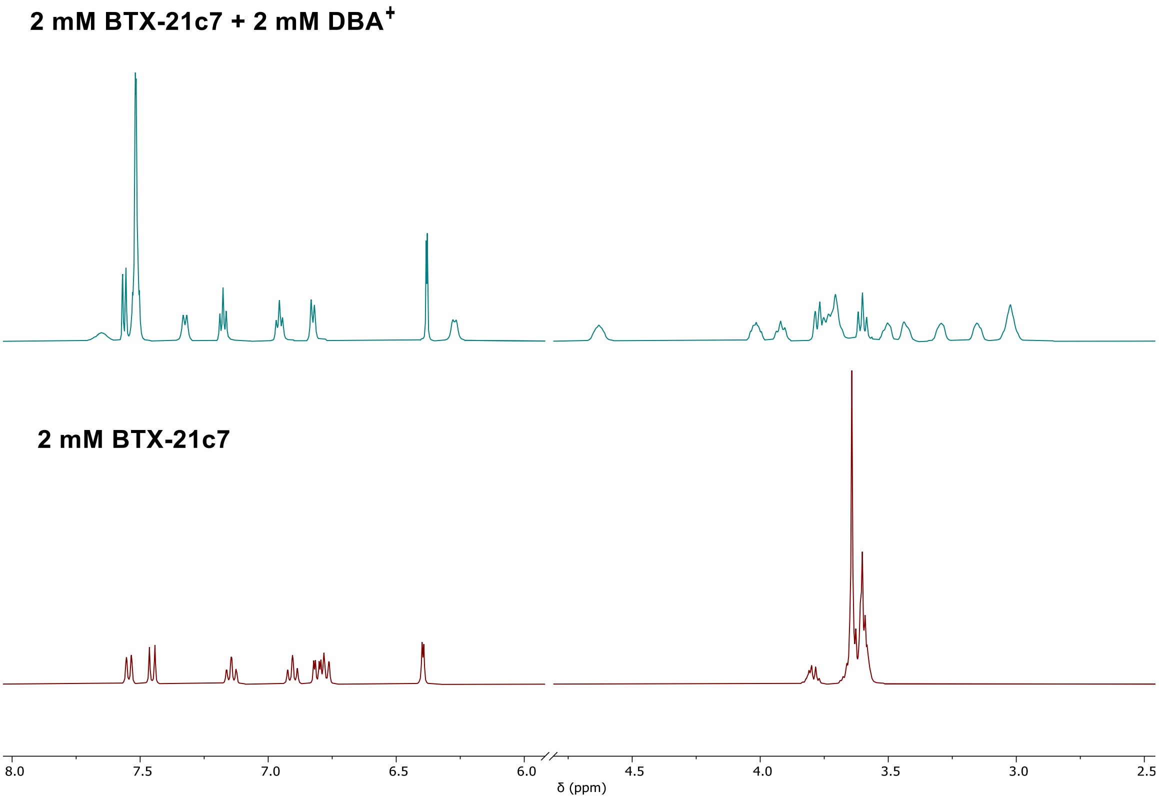

Figure S15. Stacked ^{1}H NMR spectra of 2 mM **BTX-21c7*anti*** in CD_2Cl_2 in the presence (top) and absence (bottom) of 1 equiv. DBA^+.

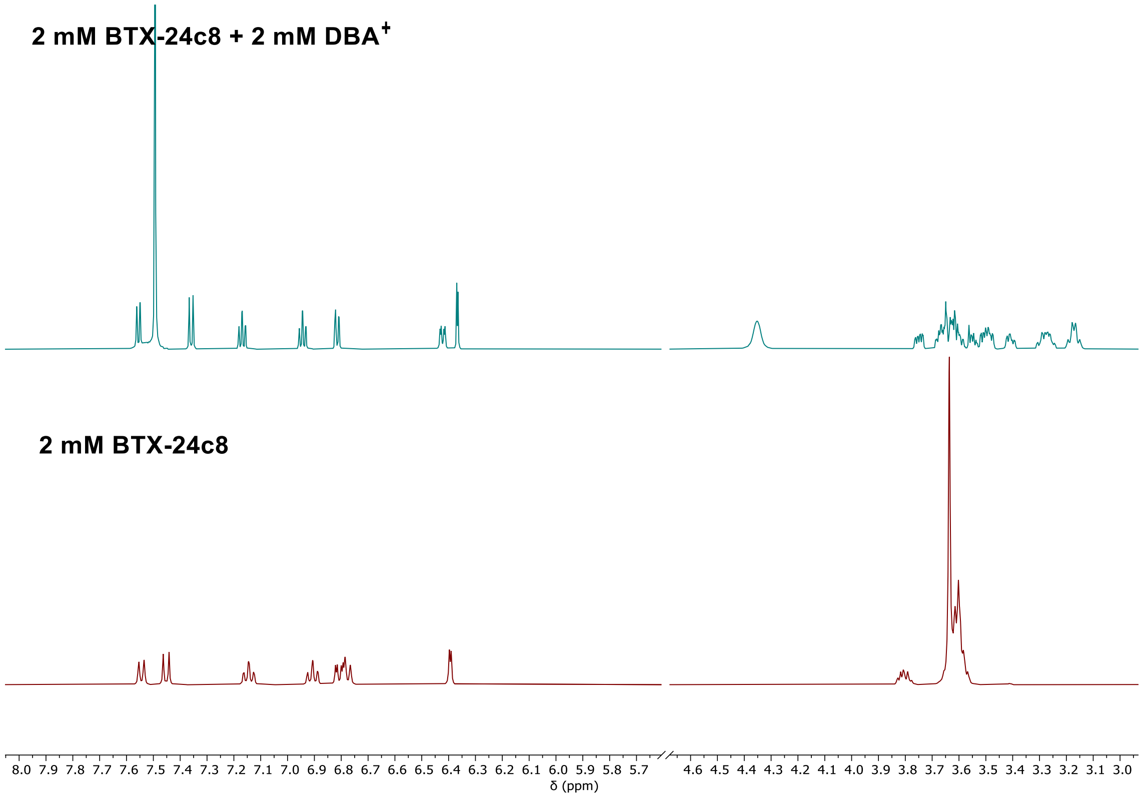

Figure S16. Stacked ^{1}H NMR spectra of 2 mM **BTX-24c8*anti*** in CD_2Cl_2 in the presence (top) and absence (bottom) of 1 equiv. DBA^+.

4.2 ^{1}H NMR Titrations

Upon *in situ* irradiation of the **BTX-crown ethers** noticeable down-field shifts of all proton environments were observed, whereby the overall number of distinct proton resonances remained constant, in good agreement with the retention of a symmetric structure and switching to the *syn*-folded state, see Figure 2B.

Figures S17-20 representatively show the changes of the ^{1}H NMR spectra of the BTX-crown ethers in both the *anti*- and *syn*-folded states upon addition of increasing cation concentrations (shown here for **BTX-21c7** upon titration with K^+ or DBA^+). The spectral changes for all the other ^{1}H NMR titrations are qualitatively analogous.

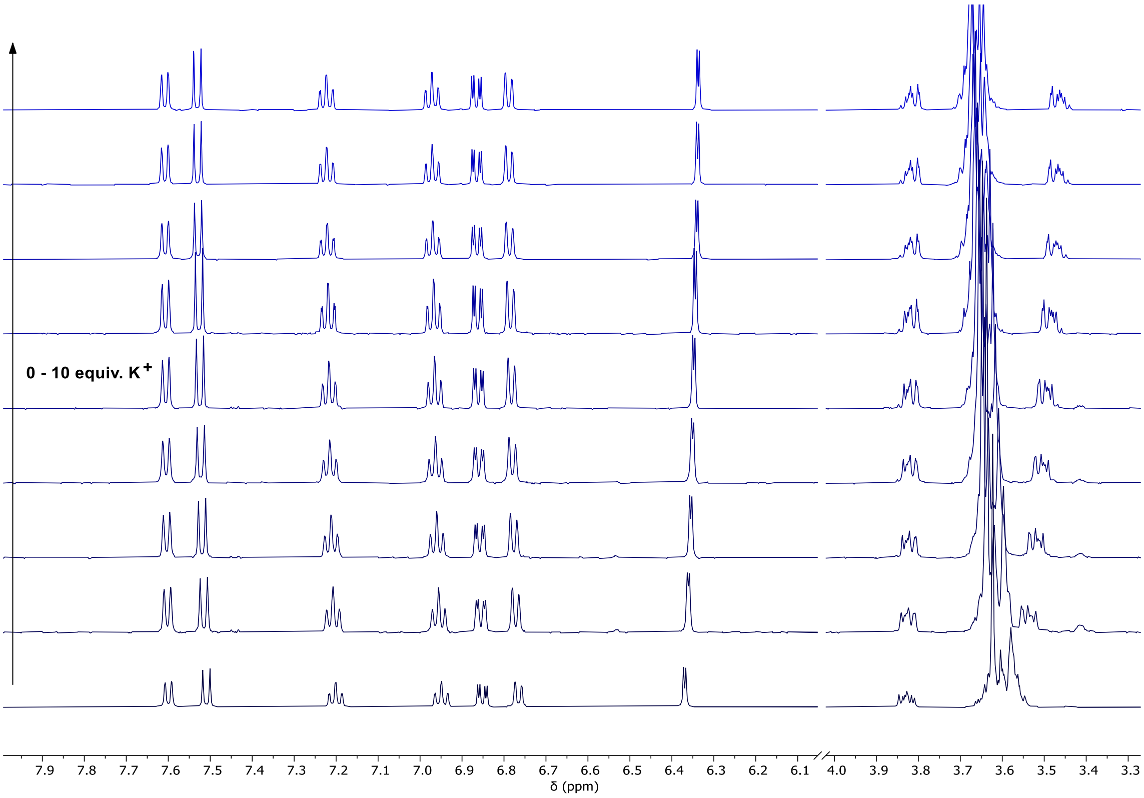

Figure S17. Stacked ^{1}H NMR spectra of 1 mM **BTX-21c7***anti* in CD_3CN/CD_2Cl_2 7:3 in the *anti*-folded state upon addition of increasing K^+ between 0 equiv. (bottom) and 10 equiv. (top).

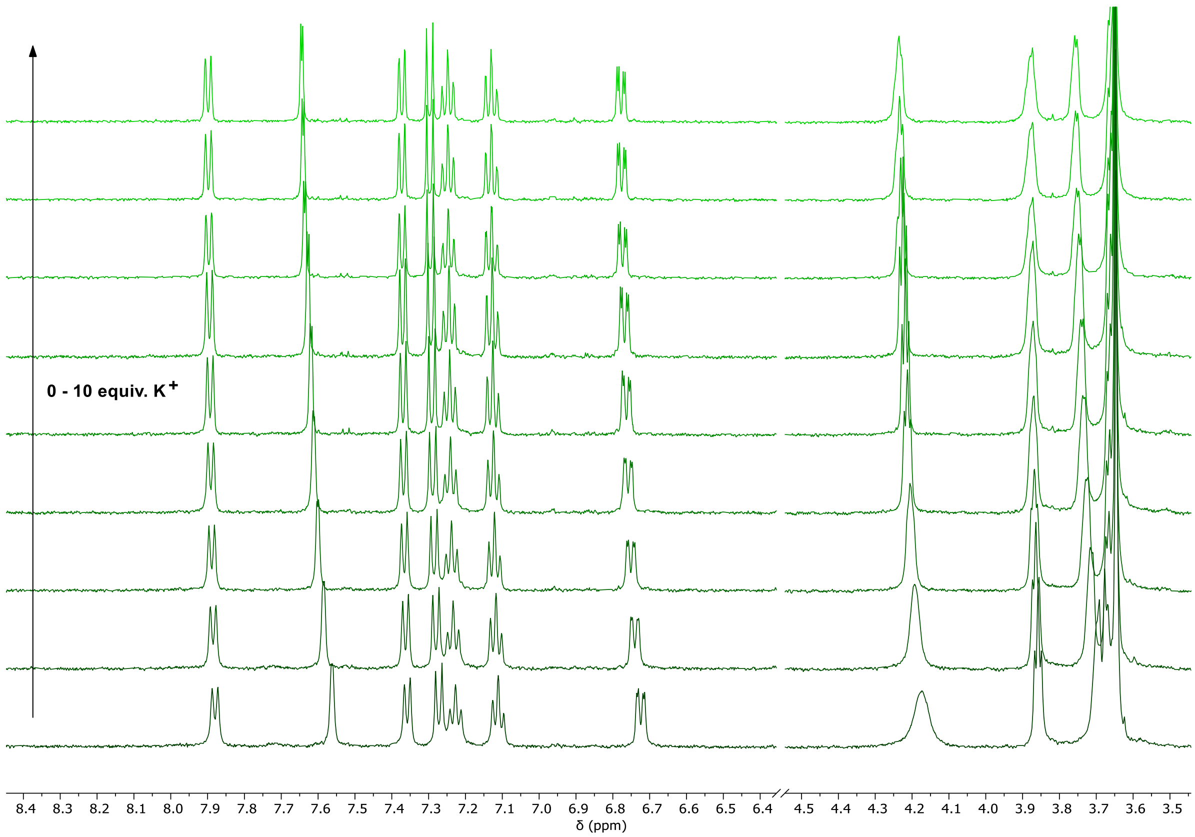

Figure S18. Stacked ^{1}H NMR spectra of 1 mM **BTX-21c7*syn*** in CD_3CN/CD_2Cl_2 7:3 in the *syn*-folded state upon addition of increasing K^+ between 0 equiv. (bottom) and 10 equiv. (top).

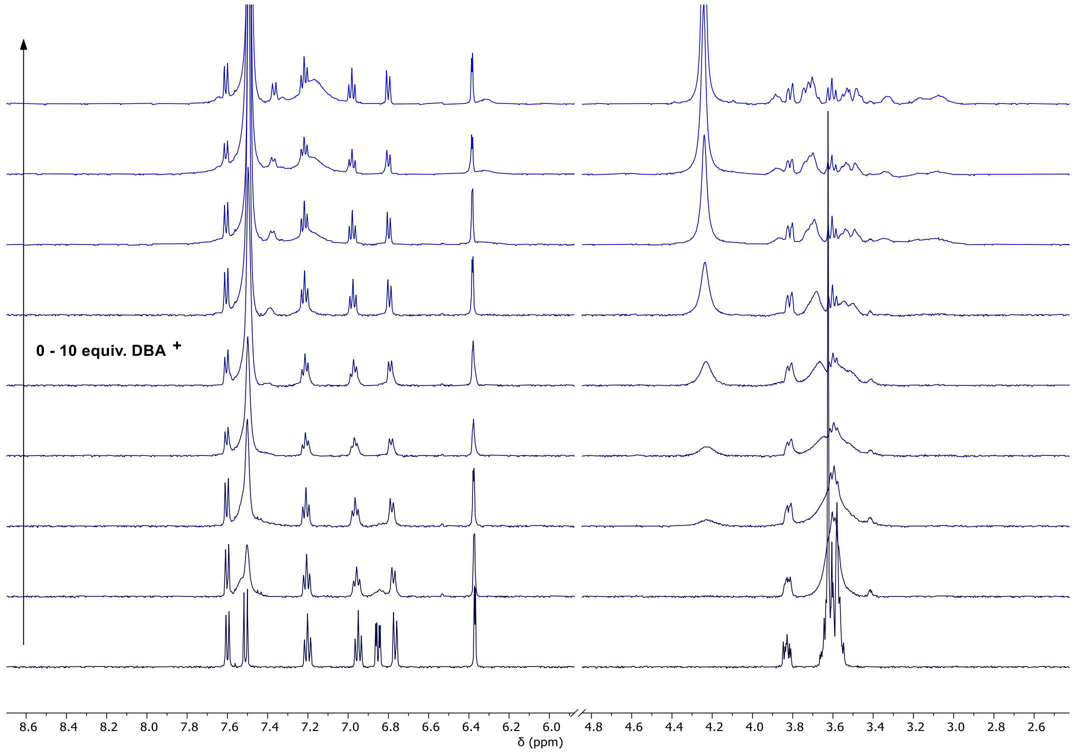

Figure S19. Stacked ^{1}H NMR spectra of 1 mM **BTX-21c7*anti*** in CD_3CN/CD_2Cl_2 7:3 in the *anti*-folded state upon addition of increasing DBA^+ between 0 equiv. (bottom) and 10 equiv. (top).

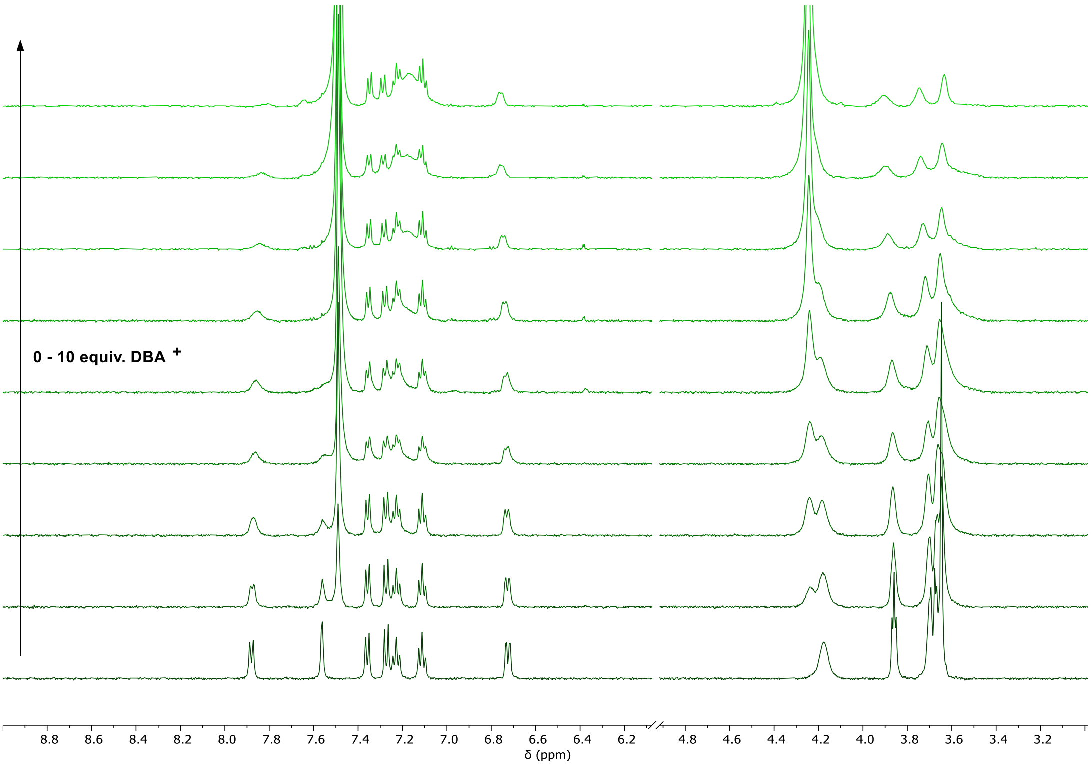

Figure S20. Stacked ^{1}H NMR spectra of 1 mM **BTX-21c7*syn*** in CD_3CN/CD_2Cl_2 7:3 in the *anti*-folded state upon addition of increasing DBA^+ between 0 equiv. (bottom) and 10 equiv. (top).

From these ^{1}H NMR titrations the 1:1 host-guest stoichiometric cation binding constants were determined by fitting the shift of at least three different protons with a global fitting procedure.[1] All these isotherms and the respective fits are shown in the following (Figures S21-32).

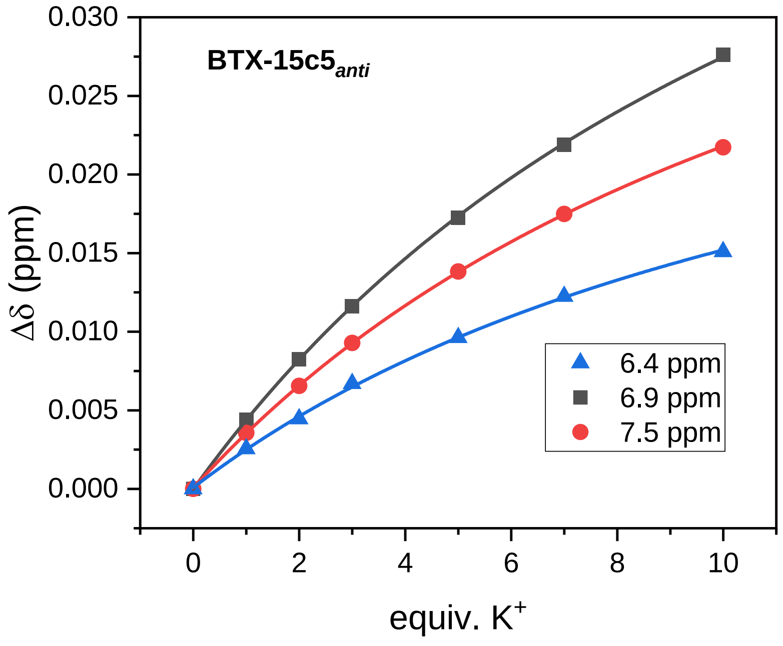

Figure S21. Changes in chemical shift of different protons as a function of K^+ concentration of **BTX-15c5*anti*** (1 mM in CD_3CN/CD_2Cl_2 7:3). The solid lines represent fits to a 1:1 host-guest stoichiometric binding model.

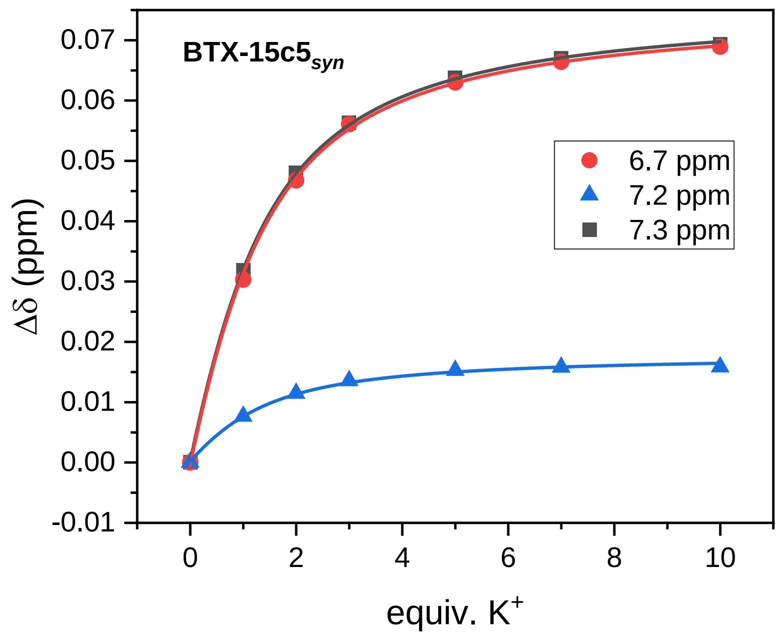

Figure S22. Changes in chemical shift of different protons as a function of K^+ concentration of **BTX-15c5*syn*** (1 mM in CD_3CN/CD_2Cl_2 7:3). The solid lines represent fits to a 1:1 host-guest stoichiometric binding model.

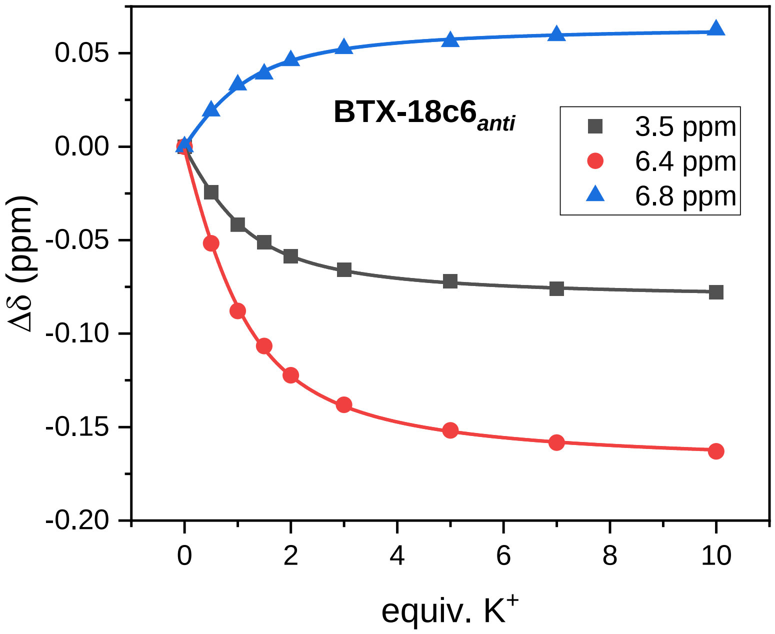

Figure S23. Changes in chemical shift of different protons as a function of K^+ concentration of **BTX-18c6*anti*** (1 mM in CD_3CN/CD_2Cl_2 7:3). The solid lines represent fits to a 1:1 host-guest stoichiometric binding model.

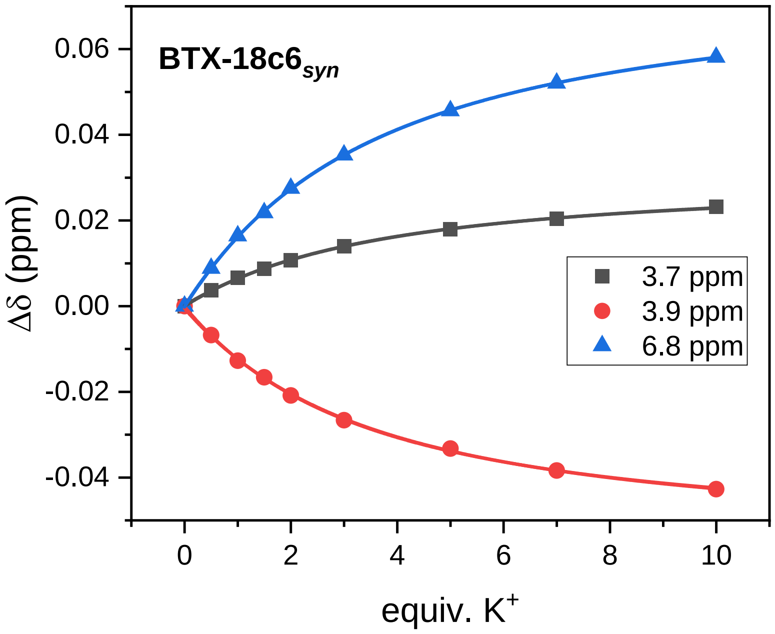

Figure S24. Changes in chemical shift of different protons as a function of K^+ concentration of **BTX-18c6*syn*** (1 mM in CD_3CN/CD_2Cl_2 7:3). The solid lines represent fits to a 1:1 host-guest stoichiometric binding model.

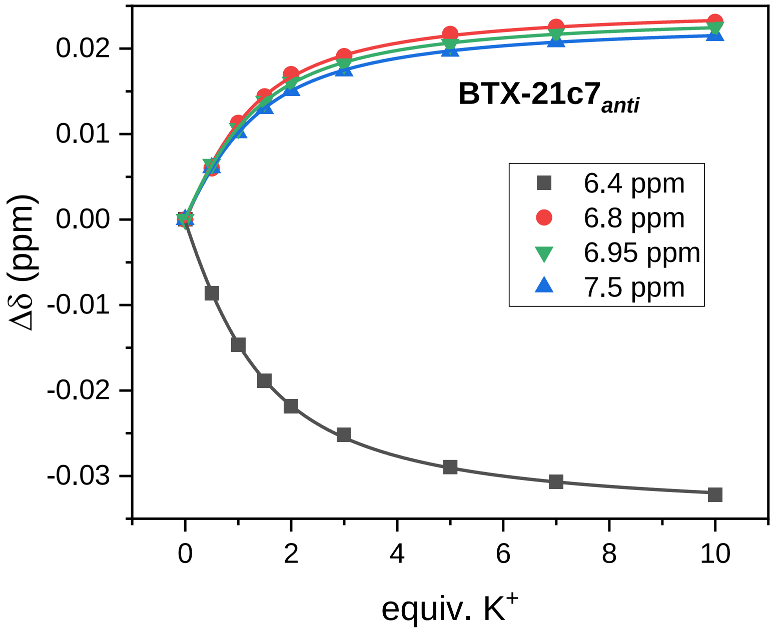

Figure S25. Changes in chemical shift of different protons as a function of K^+ concentration of **BTX-21c7*anti*** (1 mM in CD_3CN/CD_2Cl_2 7:3). The solid lines represent fits to a 1:1 host-guest stoichiometric binding model.

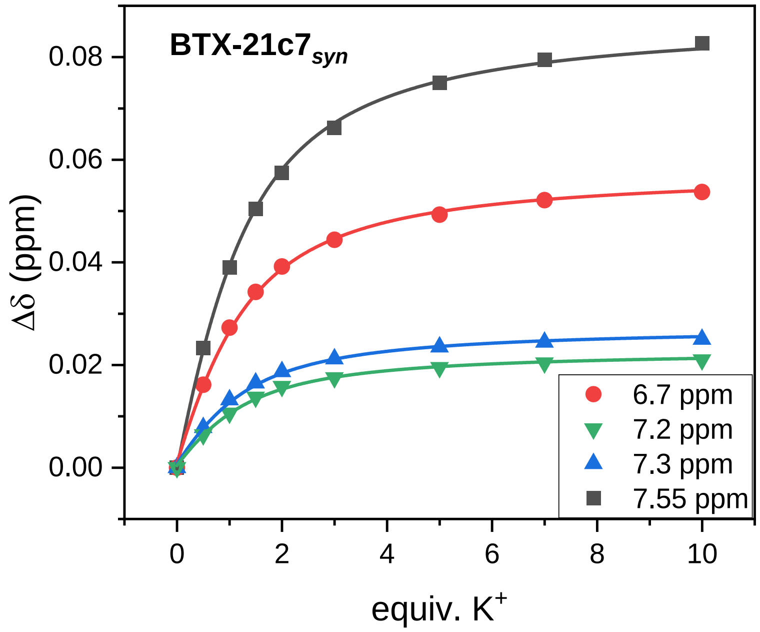

Figure S26. Changes in chemical shift of different protons as a function of K^+ concentration of **BTX-21c7*syn*** (1 mM in CD_3CN/CD_2Cl_2 7:3). The solid lines represent fits to a 1:1 host-guest stoichiometric binding model.

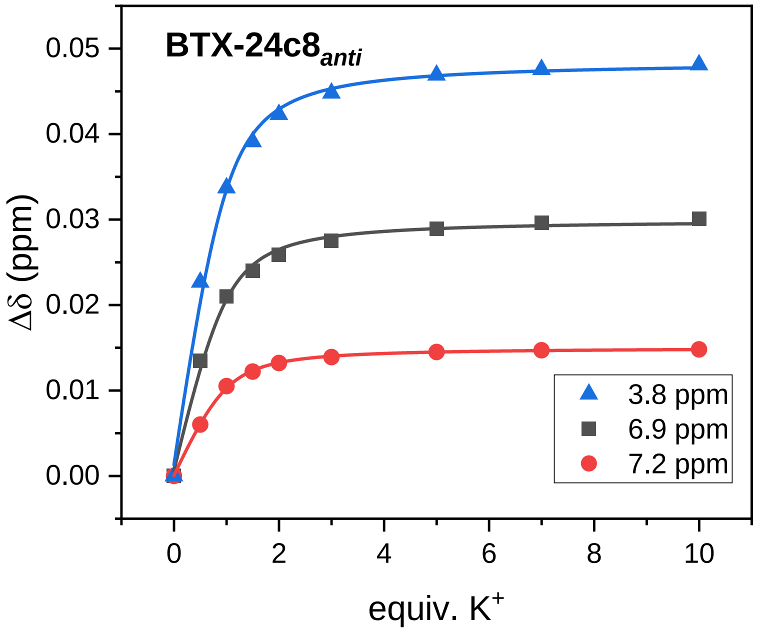

Figure S27. Changes in chemical shift of different protons as a function of K^+ concentration of **BTX-24c8*anti*** (1 mM in CD_3CN/CD_2Cl_2 7:3). The solid lines represent fits to a 1:1 host-guest stoichiometric binding model.

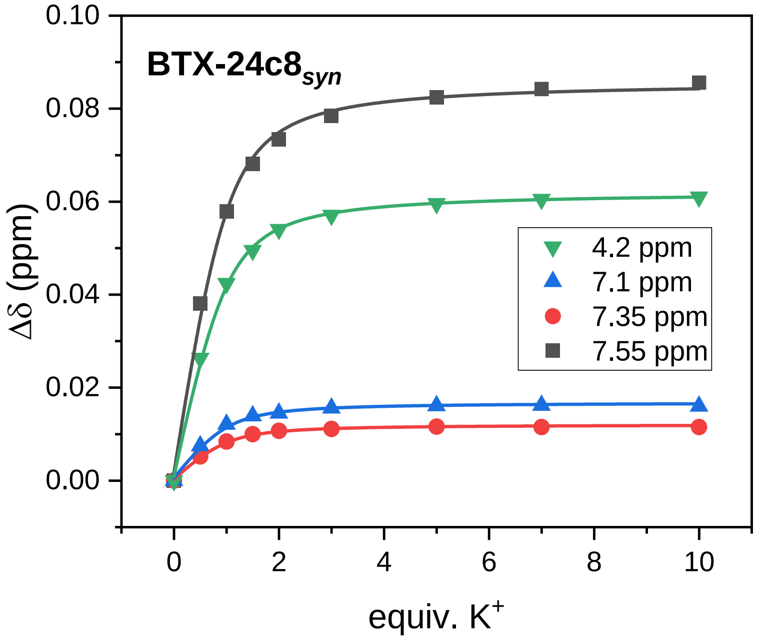

Figure S28. Changes in chemical shift of different protons as a function of K^+ concentration of **BTX-24c8*syn*** (1 mM in CD_3CN/CD_2Cl_2 7:3). The solid lines represent fits to a 1:1 host-guest stoichiometric binding model.

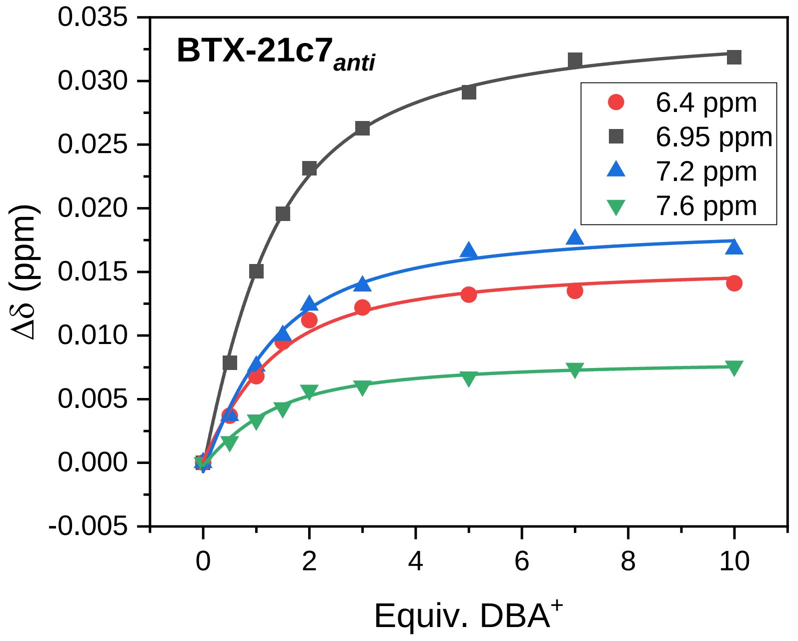

Figure S29. Changes in chemical shift of different protons as a function of DBA⁺ concentration of **BTX-21c7*anti*** (1 mM in CD_3CN/CD_2Cl_2 7:3). The solid lines represent fits to a 1:1 host-guest stoichiometric binding model.

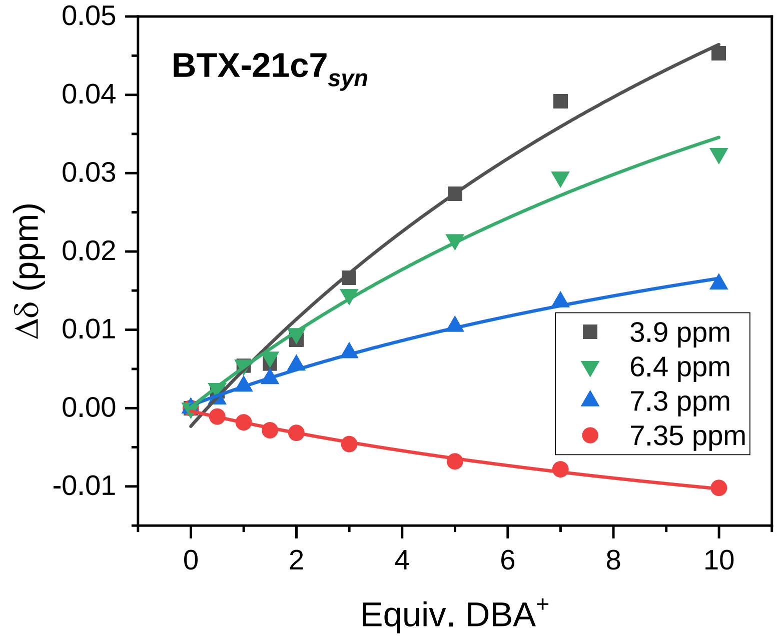

Figure S30. Changes in chemical shift of different protons as a function of DBA⁺ concentration of **BTX-21c7*syn*** (1 mM in CD_3CN/CD_2Cl_2 7:3). The solid lines represent fits to a 1:1 host-guest stoichiometric binding model.

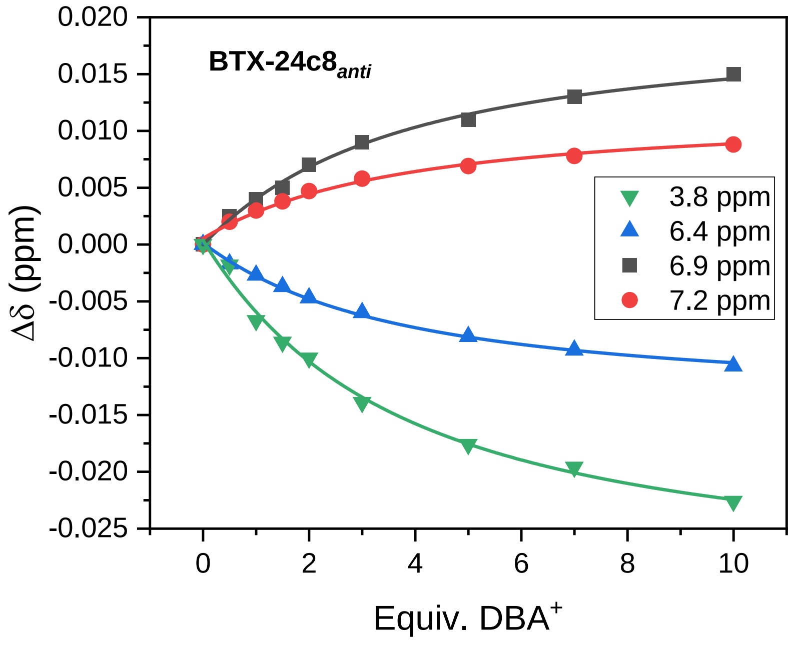

Figure S31. Changes in chemical shift of different protons as a function of DBA⁺ concentration of **BTX-24c8*anti*** (1 mM in CD_3CN/CD_2Cl_2 7:3). The solid lines represent fits to a 1:1 host-guest stoichiometric binding model.

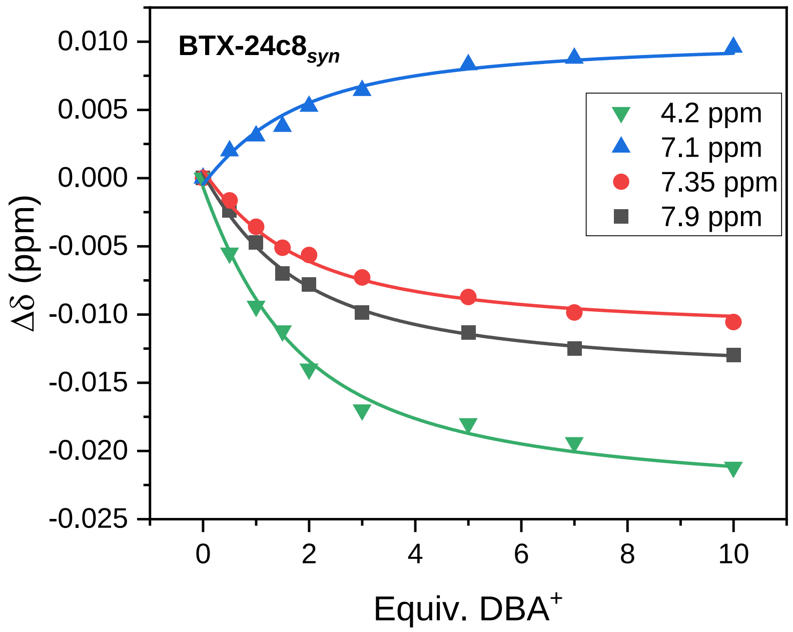

Figure S32. Changes in chemical shift of different protons as a function of DBA⁺ concentration of **BTX-24c8*syn*** (1 mM in CD_3CN/CD_2Cl_2 7:3). The solid lines represent fits to a 1:1 host-guest stoichiometric binding model.

Figure S33 shows a representative stack of ^{1}H NMR spectra of **BTX-24c8^{2+}(ClO_4^-)$_2$** upon titration with cations in CD_3CN/CD_2Cl_2 7:3 (here shown for titration with K^+, the corresponding spectral changes for titration with DBA^+ are qualitatively analogous). Fitting of the binding isotherms (Figures S34-35) was carried out as described above, revealing no binding for either cation ($K < 1\ M^{-1}$).

In both cases the ^{1}H NMR spectra of **BTX-24c8^{2+}(ClO_4^-)$_2$** in the presence of 10 equiv. of either DBA^+ or K^+, were completely unchanged after 10 days, highlighting the high chemical stability of the dicationic redox state under ambient conditions.

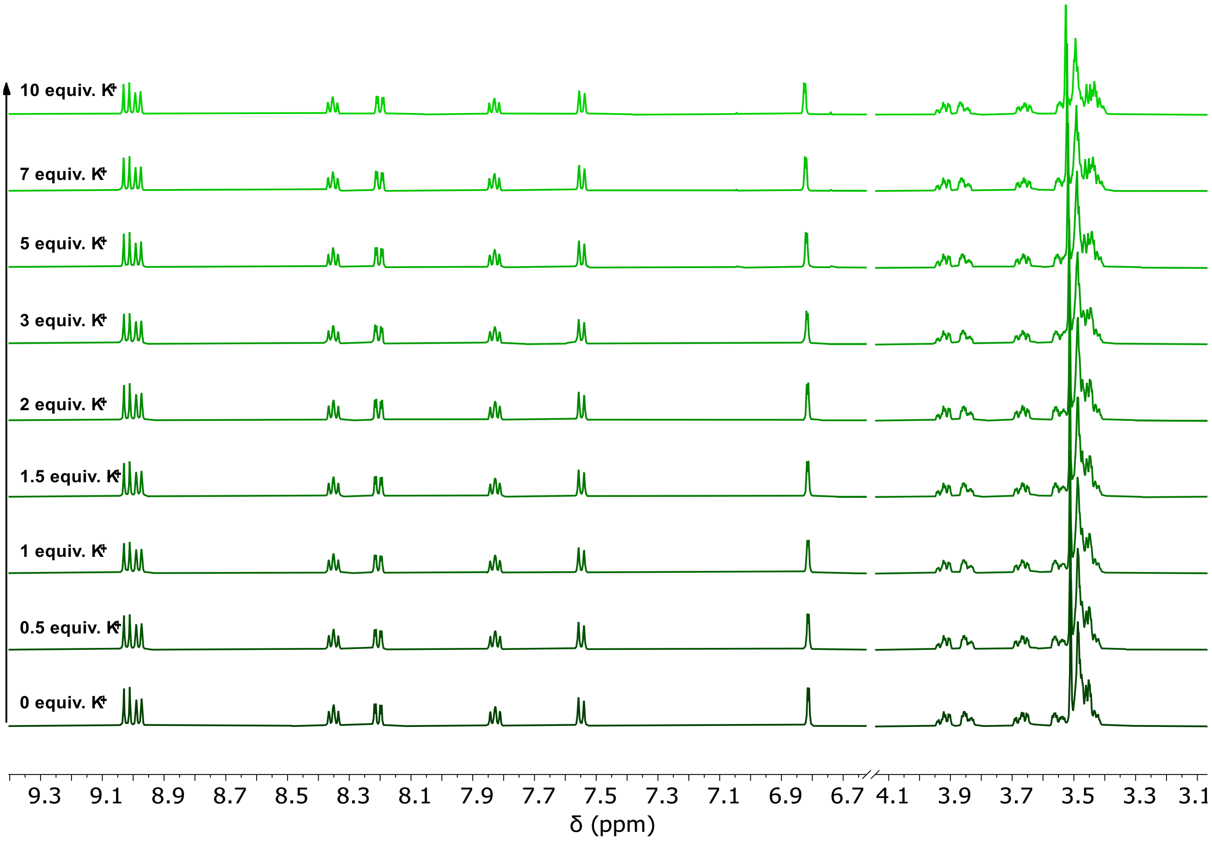

Figure S33. ^{1}H NMR spectra of **BTX-24c8^{2+}(ClO_4^-)$_2$** upon titration with KPF_6 in CD_3CN/CD_2Cl_2 7:3.

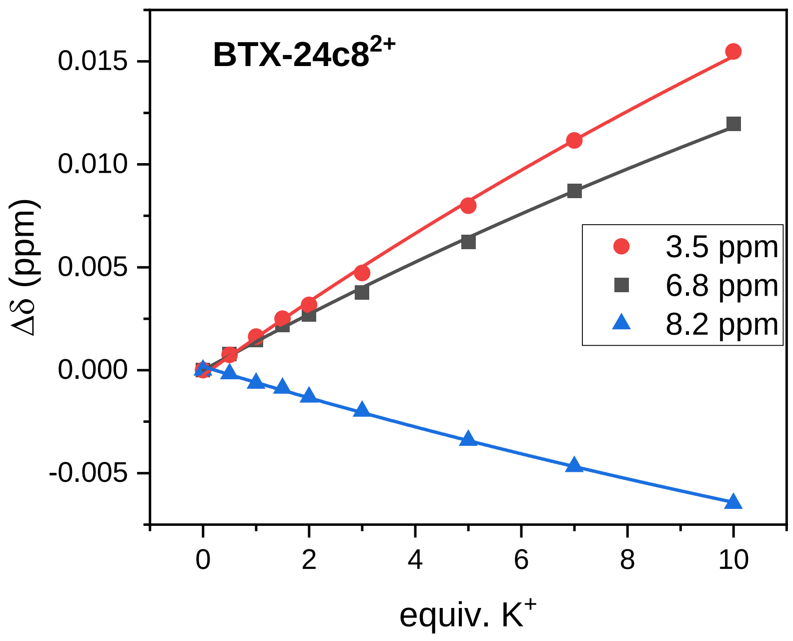

Figure S34. ^{1}H NMR chemical shifts of select protons of **BTX-24c8^{2+}(ClO_4^-)$_2$** upon titration with KPF_6 in CD_3CN/CD_2Cl_2 7:3. The solid lines represent fits to a 1:1 stoichiometric host-guest binding model.

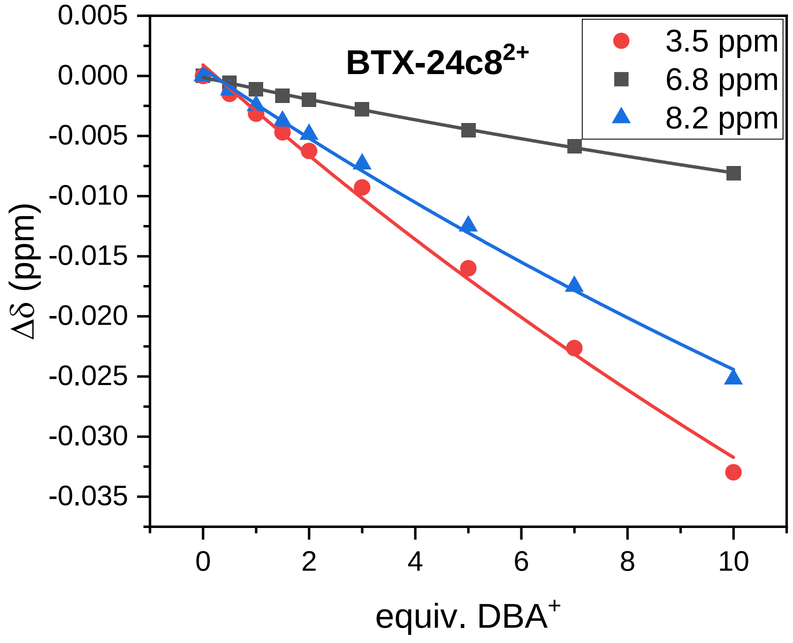

Figure S35. ^{1}H NMR chemical shifts of select protons of **BTX-24c8^{2+}(ClO_4^-)$_2$** upon titration with $DBAPF_6$ in CD_3CN/CD_2Cl_2 7:3. The solid lines represent fits to a 1:1 stoichiometric host-guest binding model.

4.3 ^{1}H NMR Shuttling Experiments

The degree of complexation of the DBA$^+$ guest with either the **BTX-21c7** or **BTX-24c8** hosts in a mixture of the three components (here 1 mM each) cannot be directly measured, as there are no unique spectroscopic signals to directly differentiate **BTX-21c7** from **BTX-24c8**. For example, their ^{1}H NMR spectra (as free hosts) are virtually identical and almost perfectly overlap. Nevertheless, each of these hosts individually displays slightly different proton chemical shift changes upon titration with DBA$^+$ in both folded states (e.g. see Figures S19-20 for the chemical shifts upon titration of **BTX-21c7** with DBA$^+$ and the corresponding binding isotherms in Figures S29-32). As a result, the binding preference of DBA$^+$ in the presence of both hosts in the same mixture can be elucidated by comparison of the experimental spectra of the equimolar mixture of the three components with calculated spectra of such a mixture at different host-occupancies. The latter were obtained by simple addition of the individual experimental spectra of **BTX-21c7●**DBA$^+$ and **BTX-24c8●**DBA$^+$ (from their separate titration experiments) at different DBA$^+$ occupancies/ratios (carried out in MestreNova). In this manner a range of different ^{1}H NMR spectra of the mixture can be simulated which correspond to different host-occupancies ranging from, for example, exclusive DBA$^+$ binding by one of the hosts to a scenario that corresponds to the calculated occupancy in the ternary mixture. For example, the calculated spectrum 5 at the top of Figure S36 corresponds to exclusive DBA$^+$ binding to **BTX-21c7*anti*** (with 44% of the guest being bound by this host) and no binding to **BTX-24c8*anti***. This was obtained by addition of the individual spectra of **BTX-21c7*anti*** in the presence of 1 equiv. of DBA$^+$ and that of free **BTX-24c8*anti***. This corresponds to a scenario that underestimates binding to the latter host; the predicted binding preference being 40% and 13% for **BTX-21c7*anti*** and **BTX-24c8*anti***, respectively. Similarly, the convolution of **BTX-21c7*anti*** in the presence of 0.5 equiv. of DBA$^+$ and **BTX-24c8*anti*** in the presence of 0.5 equiv. of DBA$^+$ generates spectrum 4, which simulates a scenario in which DBA$^+$ binding by **BTX-24c8*anti*** is of very similar magnitude (11%) as the expected value (12%), while in this case occupancy of **BTX-21c7*anti*** is significantly underestimated (26%). In contrast, the convolution of **BTX-21c7*anti*** in the presence of 1 equiv. of DBA$^+$ and **BTX-24c8*anti*** in the presence of 1 equiv. of DBA$^+$ generates spectrum 3, whereby **BTX-21c7*anti*** occupancy (44%) is again close to the expected value (40%), while **BTX-24c8*anti*** binding is now significantly overestimated (20%). Finally, spectrum 2, obtained by the convolution of **BTX-21c7*anti*** in the presence of 1 equiv. of DBA$^+$ and **BTX-24c8*anti*** in the presence of 0.5 equiv. of DBA$^+$, simulates the scenario in which the occupancies are very close to the predicted values.

A detailed comparison of the experimental spectra of the ternary mixture (spectrum 1, bottom), both in terms of peak shape and position, with these different DBA$^+$ occupancy scenarios clearly shows that the experimental data matches best with the simulated scenario that most closely corresponds to the expected binding preferences (*i.e.* spectrum 2). This qualitative comparison was carried out for a number of different chemical shift ranges (Figures S36-38), whereby in all cases the best match was spectrum 2 (marked with two green ticks). This comparison of different proton environments also allowed to rule out certain binding

scenarios which are reasonably good fits in some cases. For example, in the 3.79 – 3.86 ppm chemical shift range (Figure S36) spectrum 4 also matches reasonably well to the experimental spectrum (marked with one green tick), however in the 7.18 – 7.23 ppm chemical shift range (Figure S37), this spectrum gives the worst agreement of all scenarios (two red crosses), thereby strongly indicating that binding scenario 4 is not realistic. Note here again that the expected binding scenario, *i.e.* scenario 2, matches very well in all cases.

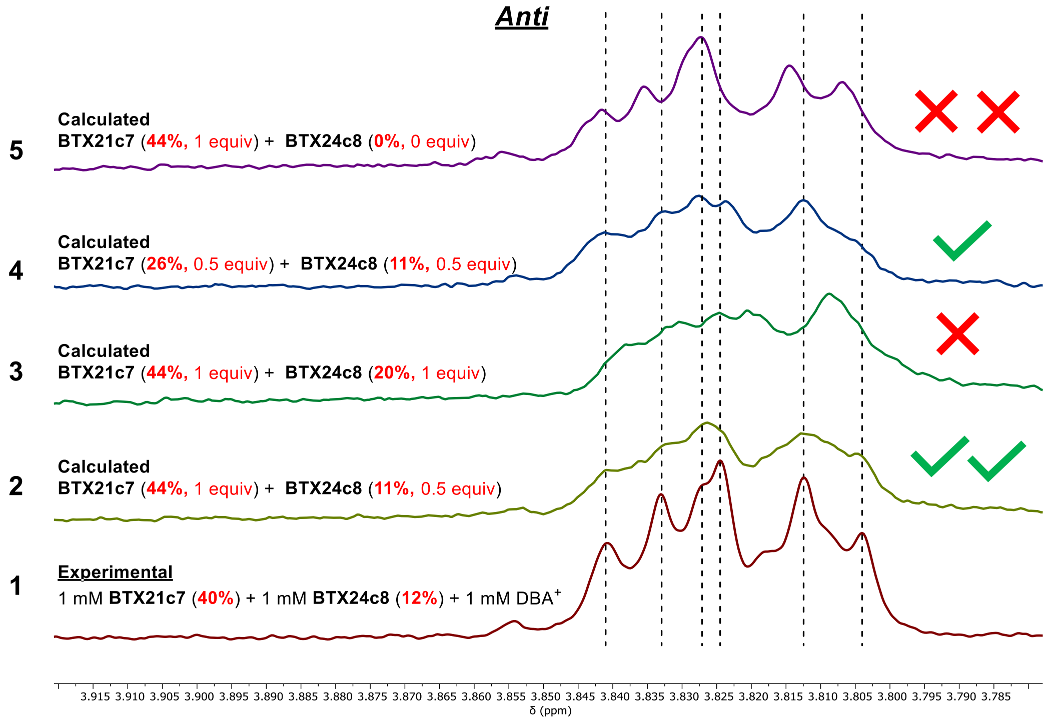

Figure S36. ^{1}H NMR comparison of simulated binding scenarios (spectra 2-5) with different DBA^{+} occupancies (red percentage), obtained by addition of the individual **BTX-21c7$_{anti}$** and **BTX-24c8$_{anti}$** spectra in the presence of different DBA^{+} equivalents, with that of the experimental ternary mixture (spectrum 1) in the chemical shift range of ~3.8 ppm. The dotted lines are meant to aid the comparison of selected peak positions of the experimental spectrum.

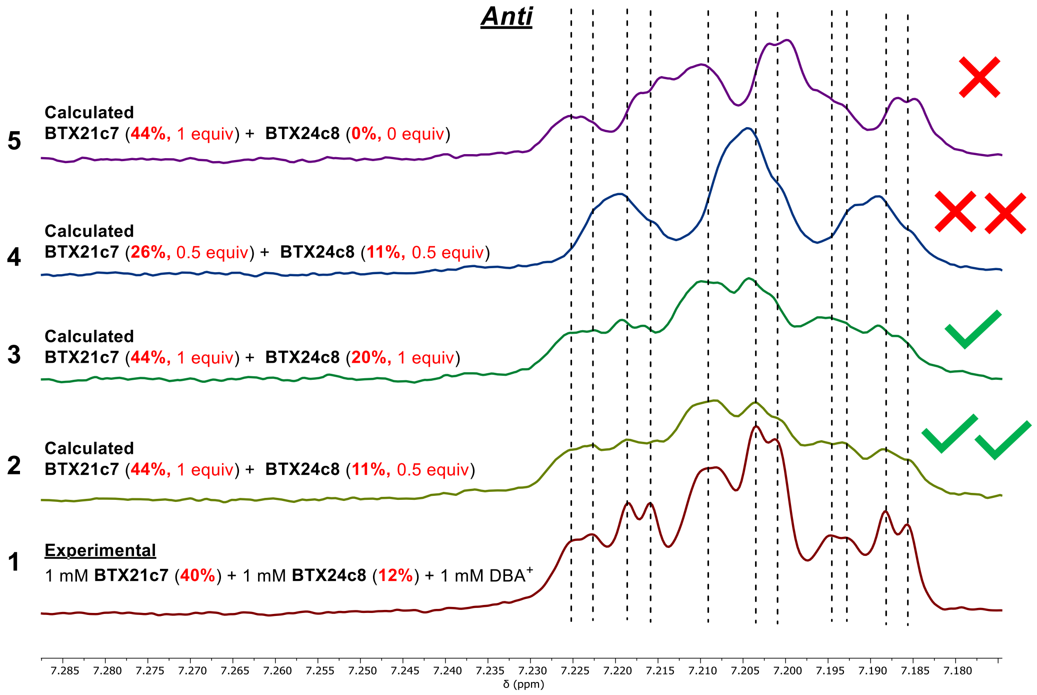

Figure S37. ^{1}H NMR comparison of simulated binding scenarios (spectra 2-5) with different DBA^+ occupancies (red percentage), obtained by addition of the individual **BTX-21c7*anti*** and **BTX-24c8*anti*** spectra in the presence of different DBA^+ equivalents, with that of the experimental ternary mixture (spectrum 1) in the chemical shift range of ~7.2 ppm. The dotted lines are meant to aid the comparison of selected peak positions of the experimental spectrum.

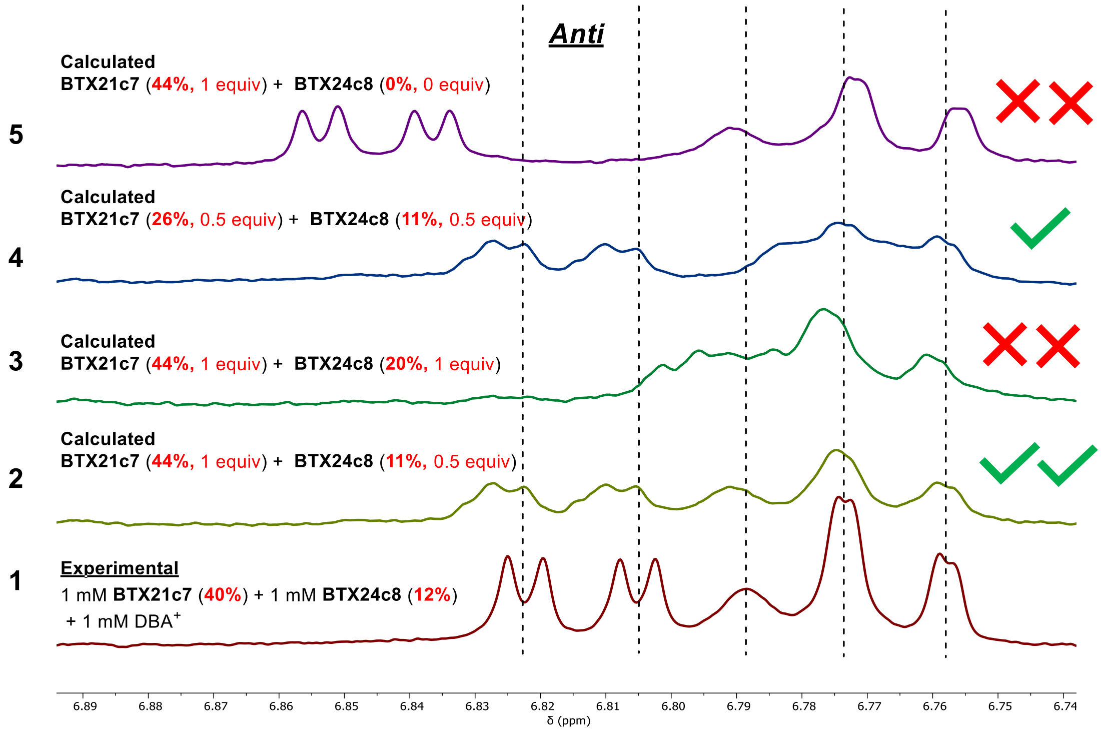

Figure S38. ^{1}H NMR comparison of simulated binding scenarios (spectra 2-5) with different DBA^+ occupancies (red percentage), obtained by addition of the individual **BTX-21c7*anti*** and **BTX-24c8*anti*** spectra in the presence of different DBA^+ equivalents, with that of the experimental ternary mixture (spectrum 1) in the chemical shift range of ~6.8 ppm. The dotted lines are meant to aid the comparison of selected peak positions of the experimental spectrum.

The same comparison was also carried out for the binding of DBA⁺ to the *syn*-folded state of the receptors (Figures S39-41), which again confirmed that the expected binding preference (spectra 2) most closely resemble the experimental results.

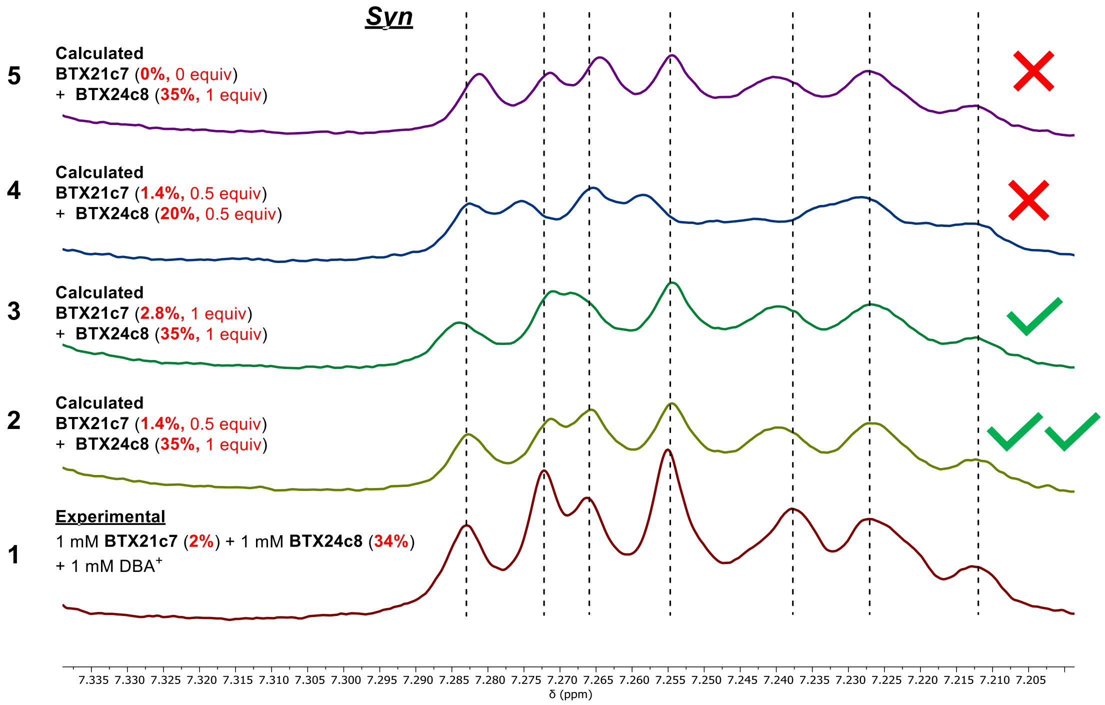

Figure S39. ^{1}H NMR comparison of simulated binding scenarios (spectra 2-5) with different DBA⁺ occupancies (red percentage), obtained by addition of the individual **BTX-21c7*syn*** and **BTX-24c8*syn*** spectra in the presence of different DBA⁺ equivalents, with that of the experimental ternary mixture (spectrum 1) in the chemical shift range of ~7.25 ppm. The dotted lines are meant to aid the comparison of selected peak positions of the experimental spectrum.

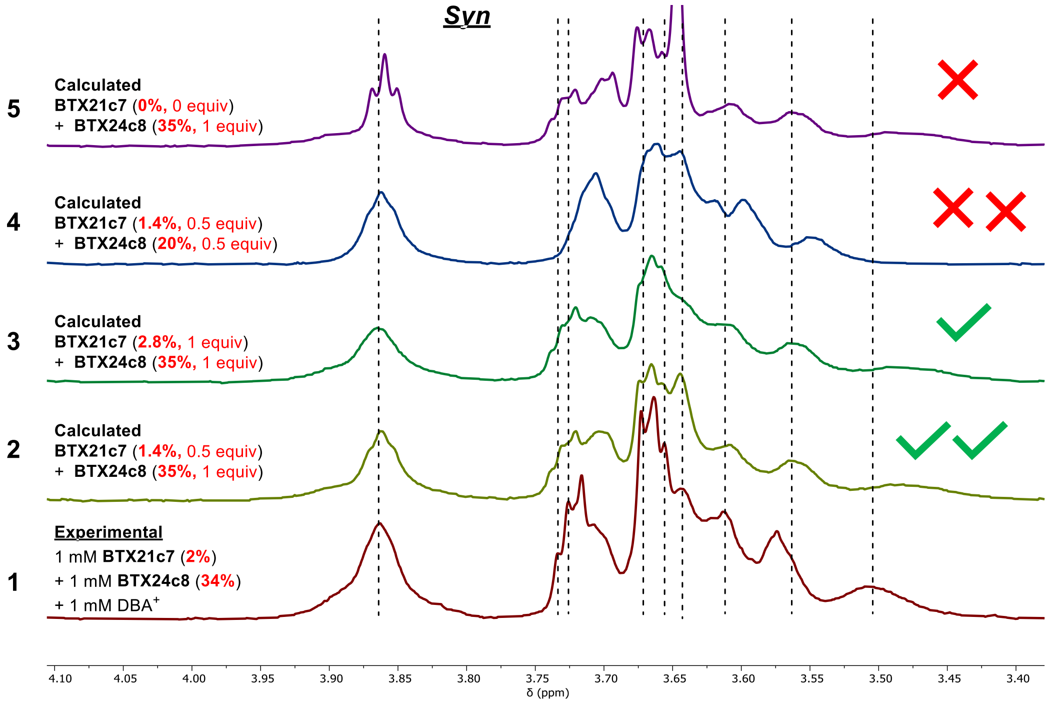

Figure S40. ^{1}H NMR comparison of simulated binding scenarios (spectra 2-5) with different DBA^{+} occupancies (red percentage), obtained by addition of the individual **BTX-21c7**$_{syn}$ and **BTX-24c8**$_{syn}$ spectra in the presence of different DBA^{+} equivalents, with that of the experimental ternary mixture (spectrum 1) in the chemical shift range of ~3.7 ppm. The dotted lines are meant to aid the comparison of selected peak positions of the experimental spectrum.

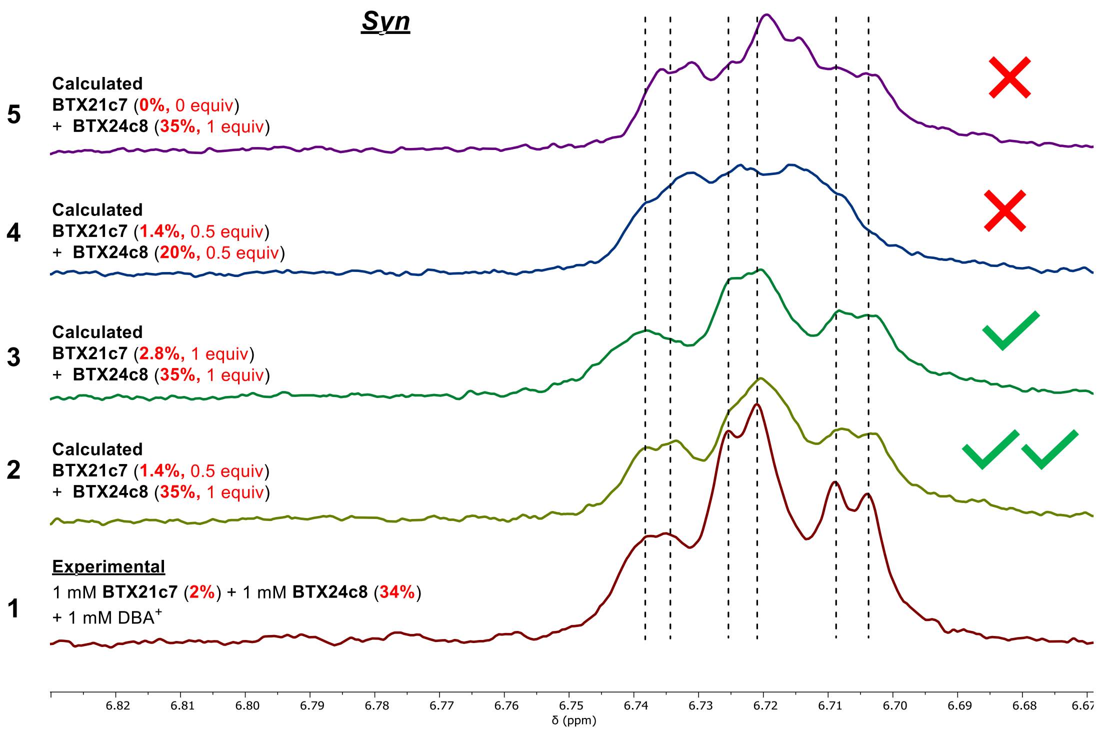

Figure S41. ^{1}H NMR comparison of simulated binding scenarios (spectra 2-5) with different DBA^{+} occupancies (red percentage), obtained by addition of the individual **BTX-21c7**$_{syn}$ and **BTX-24c8**$_{syn}$ spectra in the presence of different DBA^{+} equivalents, with that of the experimental ternary mixture (spectrum 1) in the chemical shift range of ~6.72 ppm. The dotted lines are meant to aid the comparison of selected peak positions of the experimental spectrum.

Taken together, these comparisons confirm that the binding preference of DBA^+ in the mixture follows the expected trends; there is no apparent influence of the two hosts on each other and the DBA^+ occupancy can be accurately predicted based on the individual binding constants. More generally, this also confirms that volatile (meta-stable) light-driven guest shuttling between the two hosts is indeed possible; in the absence of light (*i.e.* for the receptors in the *anti*-folded state) DBA^+ binds preferentially to **BTX-21c7** while irradiation to the *syn*-folded state induces a switch to preferential **BTX-24c8** binding, in both cases with large preference for one host.

Of further note is that such a shuttling would also be expected for K^+ between **BTX-15c5** and **BTX-18c6** (Table 1, and Table S3 *vide infra*). Similarly, for **BTX-24c8** irradiation also changes the cation selectivity, *e.g.* between K^+ and DBA^+, highlighting the versatility of these switching systems (Table 1, Table S1, Figure 2F).

4.4 Improving Shuttling Performance

Having experimentally established that the DBA^+ guest shuttles between **BTX-21c7** and **BTX-24c8** hosts upon irradiation, as predicted based on their individual binding affinities, a range of scenarios in which the shuttling efficiency is further improved can be simulated.[2] Specifically, while the relative shuttling performance in the case of 1 mM DBA, 1 mM **BTX-21c7** and 1 mM **BTX-24c8** is already highly significant, the overall fraction of bound (and thus shuttling) DBA^+ guest can be improved. In this context, the following crucial parameters can be elaborated upon in more detail;

Occupancy:

This parameter defines how much of the DBA^+ guest is bound by the respective crown ether hosts as well as the free, unbound amount of guest. This can be expressed as the mole fraction χ (in %) and will always be compared with respect to the overall amount of DBA^+ guest, and not with respect to the hosts, as these can be present in much larger amounts (*vide infra*). In an ideal scenario the fraction of unbound guest χ_{G} is always 0, while in the *anti*-folded state all of the guest binds only to one crown ether, e.g. **BTX-21c7*anti*** ($\chi^{anti}_{21c7\bullet\mathrm{G}} = 100\%$) with no binding to the larger crown **BTX-24c8*anti*** ($\chi^{anti}_{24c8\bullet\mathrm{G}} = 0\%$). Upon irradiation, this ratio ideally swaps to $\chi^{syn}_{21c7\bullet\mathrm{G}} = 0\%$ and $\chi^{syn}_{24c8\bullet\mathrm{G}} = 100\%$.

As detailed in the main text, under the experimentally validated conditions (1 mM of each component), the occupancies are as follows: $\chi^{anti}_{21c7\bullet\mathrm{G}} = 40\%$, $\chi^{anti}_{24c8\bullet\mathrm{G}} = 12\%$, $\chi^{syn}_{21c7\bullet\mathrm{G}} = 2\%$, $\chi^{syn}_{24c8\bullet\mathrm{G}} = 35\%$. This means that in the non-irradiated state, almost half of the DBA^+ guest is not bound ($\chi_{G_{Free}} = 48\%$). Under continuous irradiation, this value is even slightly larger ($\chi_{G_{Free}} = 63\%$). Similarly, not all of the available crown ether hosts are engaged in complexation: $\chi_{H_{bound}} = 26\%$ (no light) and $\chi_{H_{bound}} = 18\%$ (under light); note that the

maximum fraction of bound host under these conditions is 50% as the sum of crown ether hosts is twice that of the guest.

Shuttling efficiency:

Another important parameter is the shuttling efficiency, that is the relative difference in binding/occupancy between the crown ethers of different size. In a competitive host-guest equilibrium, this is typically expressed as the specificity α, which in the absence of light corresponds to the ratio of occupied **BTX-21c7*anti*** to occupied **BTX-24c8*anti***: $\alpha_{anti} = \frac{\chi^{anti}_{21c7\bullet G}}{\chi^{anti}_{24c8\bullet G}}$. Similarly, under irradiation: $\alpha_{syn} = \frac{\chi^{syn}_{21c7\bullet G}}{\chi^{syn}_{24c8\bullet G}}$. As the relative binding preference switches towards the larger **BTX-24c8*syn***, the inverse specificity α^{-1}_{syn} is more easily interpretable in our case. For example, under our standard experimental conditions, binding to the smaller crown ether is preferred by a factor of $\alpha_{anti} = 3.3$, while irradiation leads to an even larger relative binding difference in favour of the larger crown ether of $\alpha^{-1}_{syn} = 18.2$, corresponding to an overall relative change, *i.e.* "swing", in binding preference and thus shuttling of $s = \alpha_{anti} * \alpha^{-1}_{syn} = 60.0$. This drastic difference in relative preference nicely illustrates the additive effect of the switching, whereby irradiation simultaneously lowers preference for one crown (**BTX-21c7**) *and* increases preference for the larger crown (**BTX-24c8**).

Note that the maximum specificity α corresponds to the ratio of the binding constants between the hosts in the respective conformational states, i.e. $\alpha_{anti_{max}} = \frac{K_{21c7_{anti}}}{K_{24c8_{anti}}} = \frac{1390\ M^{-1}}{300\ M^{-1}} = 4.6$ and $\alpha^{-1}_{syn_{max}} = \frac{820\ M^{-1}}{30\ M^{-1}} = 27.3$, corresponding to $s_{max} = 125.6$. This maximum shuttling efficiency is dependent on the concentration of all components and is generally higher when the concentration of all components is lower.

As shown in Table S2, both the occupancies as well as the overall shuttling efficiency can be rationally and judiciously tuned across a very large range of values by simple adjustment of the concentrations/ratios of the two hosts and the guest. For example, by keeping the ratio between all components at 1:1:1, but increasing their concentration to 5, 10 or 100 mM each, the complexation equilibrium is expectedly shifted to an overall higher degree of complexation, i.e. the fraction of unbound guest drops drastically (see entries 2-4 and Figure S42A-B). For example, at 100 mM each, only 1% and 5 % of **DBA$^+$** is unbound in the absence and presence of light, respectively, i.e. virtually all of the guest is bound (and shuttled) at all times. However, this overall increase in occupancy comes at the cost of a loss of specificity α, such that the overall shuttling efficiency *s* drops to 13, which is still very large.

Table S2. Simulated occupancies χ, specificities α and shuttling efficiencies s for different ratios/concentrations of **BTX-21c7**, **BTX-24c8** (H_1 and H_2) and **DBA⁺** (G) in the absence and presence of light.

		No light					Irradiated					
	**[H₁]/[H₂]/[G] (mM)**	$\chi^{anti}_{21c7\bullet G}$	$\chi^{anti}_{24c8\bullet G}$	$\chi_{G_{Free}}$	$\chi_{H_{Bound}}$	α_{anti}	$\chi^{syn}_{21c7\bullet G}$	$\chi^{syn}_{24c8\bullet G}$	$\chi_{G_{Free}}$	$\chi_{H_{Bound}}$	α^{-1}_{syn}	s
**1**	1/1/1	**40%**	**12%**	48%	26%	3.3	**2%**	**35%**	63%	18%	18.2	**60.0**
**2**	5/5/5	**58%**	**22%**	20%	40%	2.6	**5%**	**60%**	35%	32%	11.4	**29.6**
**3**	10/10/10	**63%**	**26%**	11%	44%	2.4	**7%**	**68%**	25%	37%	9.3	**22.3**
**4**	100/100/100	**68%**	**31%**	**1%**	49%	2.2	**14%**	**81%**	**5%**	47%	5.9	**13.0**
**5**	5/5/1	**72%**	**17%**	11%	9%	4.3	**3%**	**75%**	22%	8%	21.6	**92.9**
**6**	10/10/1	**77%**	**17%**	6%	5%	4.5	**4%**	**85%**	11%	5%	24.3	**109.4**
**7**	100/100/1	**82%**	**17%**	**1%**	0.5%	4.6	**4%**	**95%**	**1%**	0.5%	26.4	**121.4**

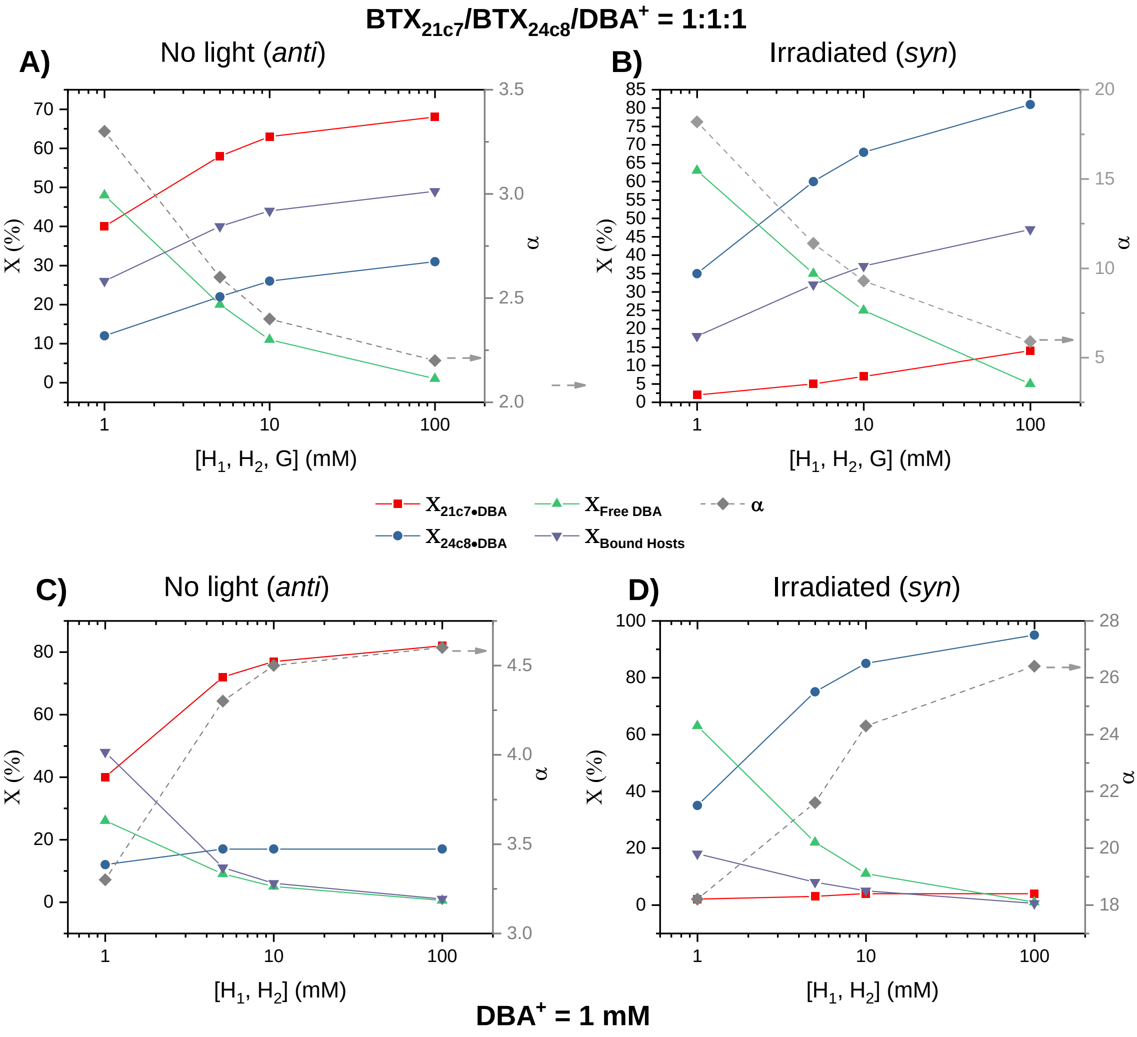

Figure S42. Simulated occupancies χ and specificities α for different ratios/concentrations of **BTX-21c7**, **BTX-24c8** (H_1 and H_2) and **DBA⁺** (G) in the absence (A and C) and presence of light (B and D). The A and B panels show the case where all components have an equal ratio, while the C and D panels correspond to the case where [**DBA⁺**] is constant at 1 mM.

Alternatively, the concentration of the **DBA⁺** guest can be kept constant (1 mM), while the concentration of the two hosts is increased (5, 10 or 100 mM each, see entries 5-7 and Figure SXXXC-D). This has a very similar effect as described above; the overall simulated guest occupancy increases drastically. For example, at 100, 100 and 1 mM **BTX-21c7**, **BTX-24c8** and **DBA⁺** (entry 7), the fraction of unbound guest is only ~1%, both in the presence and absence of light. Importantly, in this case the overall specificity and hence shuttling efficiency increases and is with *s* = 121.4 close to the theoretical maximum ($s_{max} = 125.6$). This is to say that by raising only the concentration of the two hosts, **DBA⁺** shuttling is improved both in terms of overall bound guest (~99%) *and* specificity/shuttling efficiency. (However, in this case the overall fraction of unbound, and hence "unused", host is of course larger.)

Lastly, the occupancy of the system can be improved by increasing the absolute magnitude of the binding constants, e.g. by changing the solvent systems or the specific design of the host or guest. As shown in Table 1 of the main text, K^+ also shows significantly different affinities to **BTX-15c5** and **BTX-18c6** under irradiation, which would enable a similar shuttling system. In this case binding to the larger **BTX-18c6** is preferred in the *anti*-folded state, while in the irradiated, *syn*-folded state K^+ would shuttle to **BTX-15c5**. Collated in Table S3 are the simulated occupancies and shuttling parameters for the case of 1 mM of each component. As a result of the slightly larger binding constants in the irradiated state in comparison to the DBA^+ system, the overall proportion of bound K^+ is ~50% both in the presence and absence of light, while the overall shuttling efficiency *s* is with a value of 57.4 virtually identical.

Table S3. Simulated occupancies χ, specificities α and shuttling efficiencies s for 1 mM of **BTX-15c5**, **BTX-18c6** (H_1 and H_2) and **K^+** (G) in the absence and presence of light.

	No light					Irradiated					
**[H₁]/[H₂]/[G] (mM)**	$\chi^{anti}_{15c5\bullet G}$	$\chi^{anti}_{18c6\bullet G}$	$\chi_{G_{Free}}$	$\chi_{H_{Bound}}$	α^{-1}_{anti}	$\chi^{syn}_{15c5\bullet G}$	$\chi^{syn}_{18c6\bullet G}$	$\chi_{G_{Free}}$	$\chi_{H_{Bound}}$	α_{syn}	s
**1/1/1**	**2%**	**49%**	49%	26%	20.5	**37%**	**13%**	50%	25%	2.8	**57.4**

5. Electrochemical Studies

5.1 Voltammetric Characterisation

As briefly discussed in the main text, the redox (switching) properties of all **BTX-crown ethers** are not affected by introducing the crown ether strap, as revealed by voltammetric studies. Specifically, the CVs of all receptors are qualitatively (Figure 5A) and quantitatively (Table S4) analogous to the parent **BTX-OMe**. Specifically, all redox processes, *i.e.* oxidation (from the neutral, *anti*-folded to the dicationic, orthogonal state) as well as reduction (from the dicationic, orthogonal state to the neutral, *syn*-folded state) proceed *via* virtually simultaneous two-electron transfer, thus circumventing the build-up of radical cation intermediates and endowing a high macroscopic reversibility to the redox-switching process (see Section 5.2 for further details). The generation of the *syn*-folded state upon reduction of the dication is also typically visible in the second scan of the CV, giving rise to a (smaller) new redox wave at more cathodic potentials (see peak marked with an asterisk in Figure 5A).

Table S4. Oxidation peak potentials and hysteresis of **BTX-OMe** and **BTX-crown ethers** in DCM, 100 mM $TBAPF_6$ at ν = 100 mV/s.

	**BTX-OMe**	**BTX-15c5**	**BTX-18c6**	**BTX-21c7**	**BTX-24c8**
E_{Ox}[a]	+0.80 V	+0.80 V	+0.81 V	+0.81 V	+0.81 V
ΔE_{Peak}	0.85 V	0.86 V	0.87 V	0.87 V	0.87 V

a – vs Fc/Fc^+

All receptors display excellent linear dependence of the peak currents on the square-root of the scan-rate, confirming that all redox-processes are diffusion-controlled (Figures S43-50).

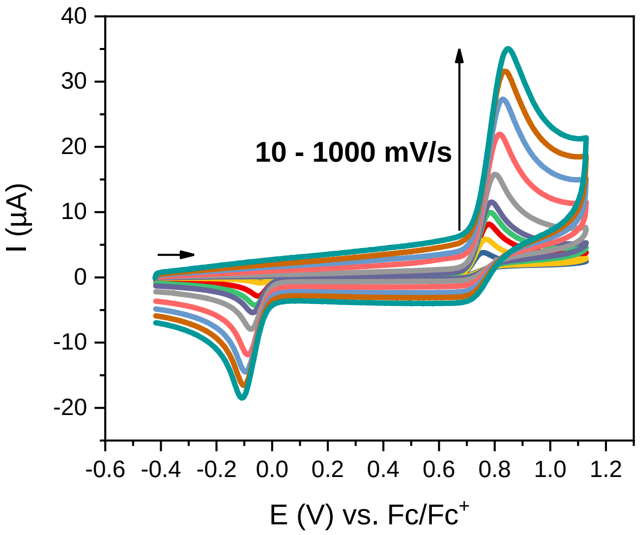

Figure S43. CVs of 0.5 mM **BTX-15c5** in DCM, 100 mM $TBAPF_6$ at different scan rates.

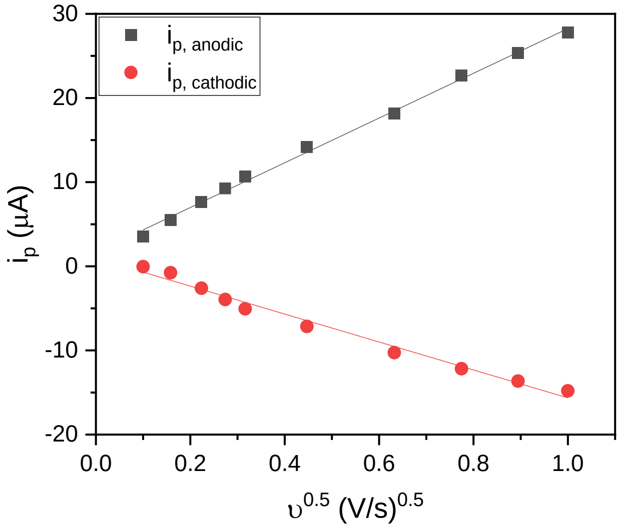

Figure S44. Anodic and cathodic peak currents of 0.5 mM **BTX-15c5** in DCM, 100 mM $TBAPF_6$ as a function of the square root of scan rate including linear fits.

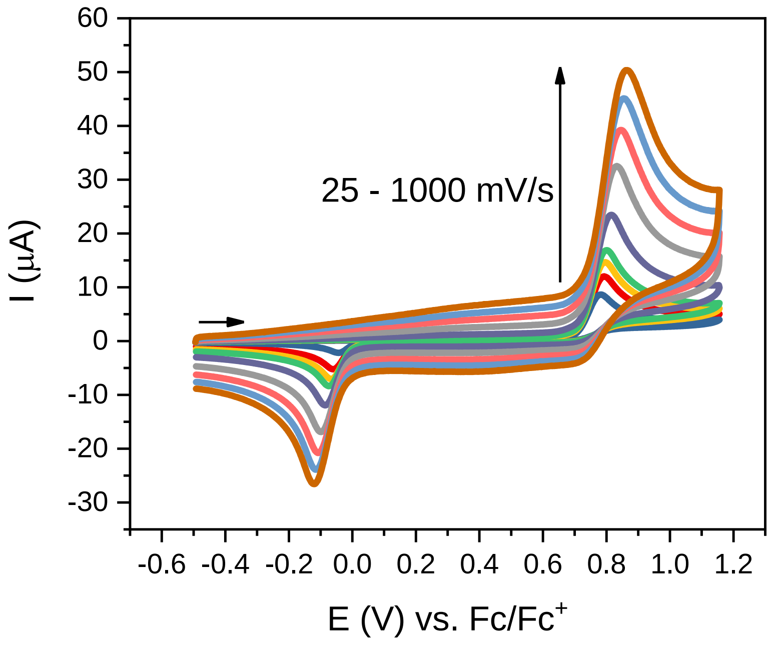

Figure S45. CVs of 0.5 mM **BTX-18c6** in DCM, 100 mM $TBAPF_6$ at different scan rates.

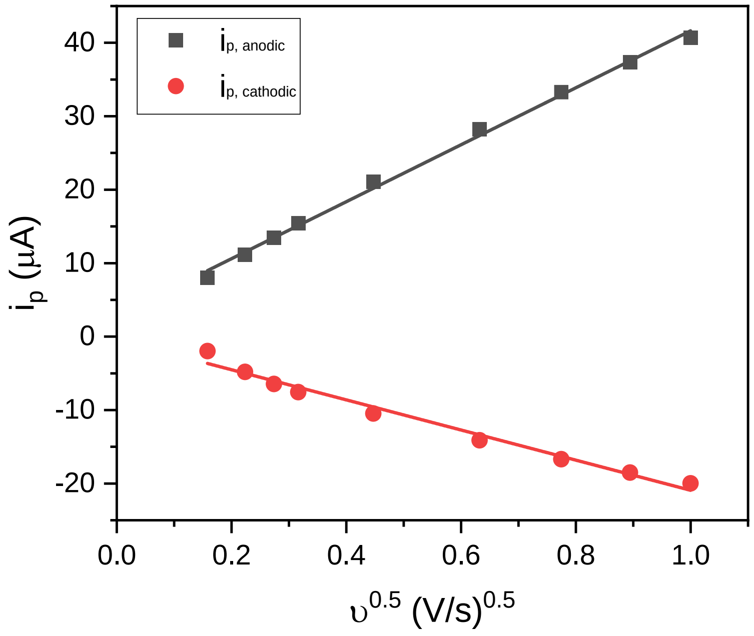

Figure S46. Anodic and cathodic peak currents of 0.5 mM **BTX-18c6** in DCM, 100 mM $TBAPF_6$ as a function of the square root of scan rate including linear fits.

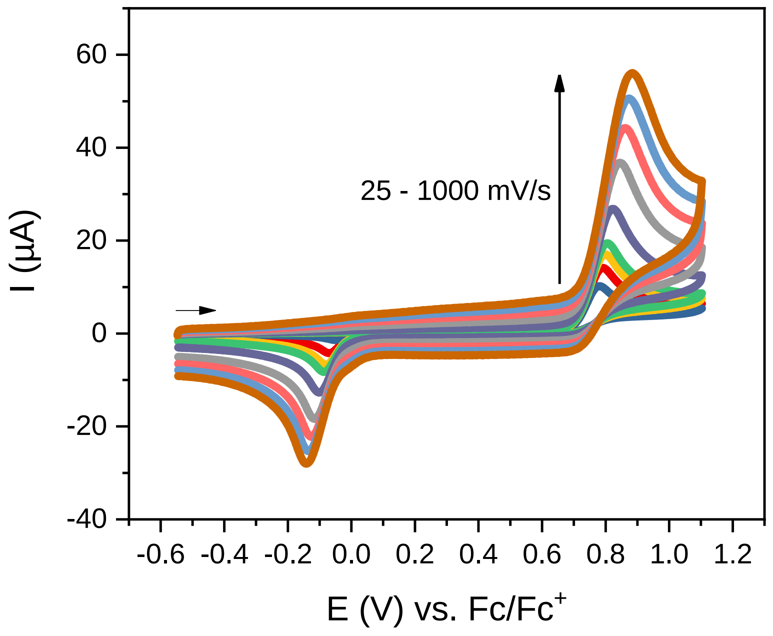

Figure S47. CVs of 0.5 mM **BTX-21c7** in DCM, 100 mM TBAPF$_6$ at different scan rates.

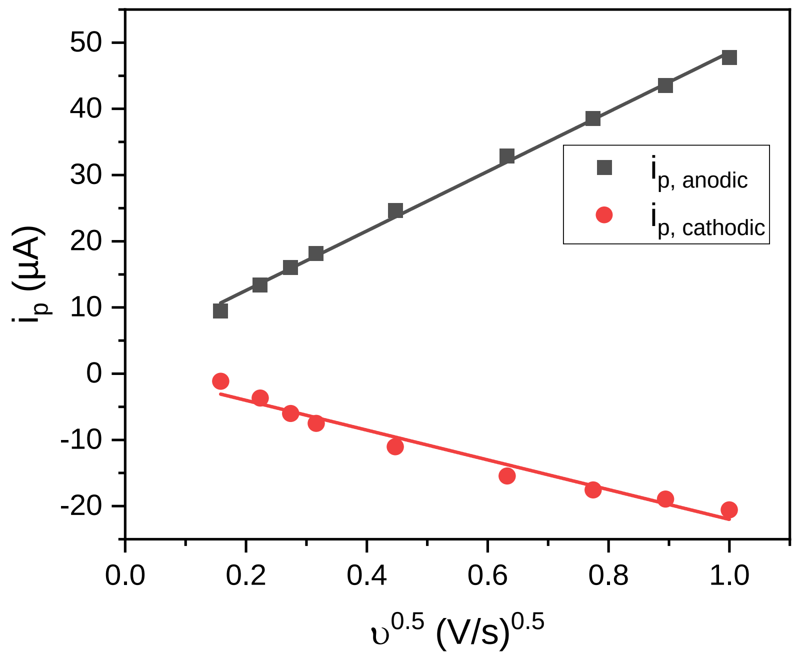

Figure S48. Anodic and cathodic peak currents of 0.5 mM **BTX-21c7** in DCM, 100 mM TBAPF$_6$ as a function of the square root of scan rate including linear fits.

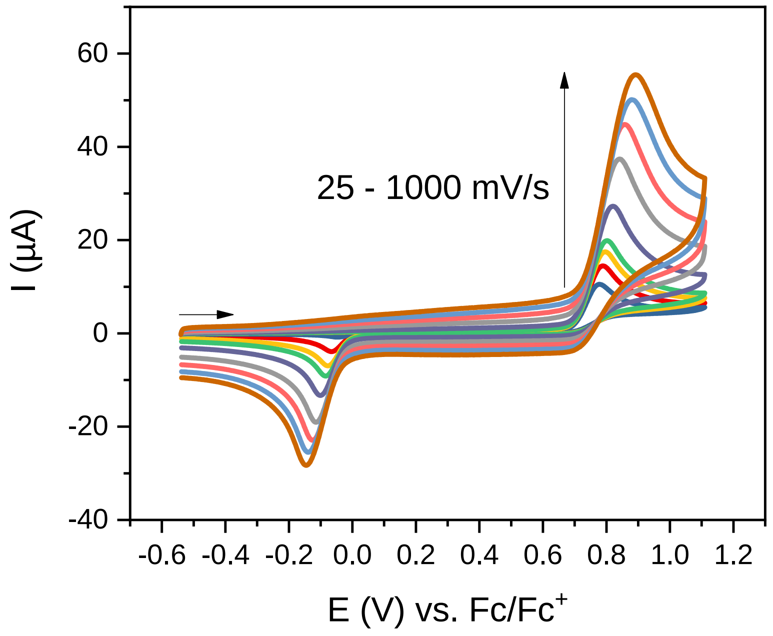

Figure S49. CVs of 0.5 mM **BTX-24c8** in DCM, 100 mM $TBAPF_6$ at different scan rates.

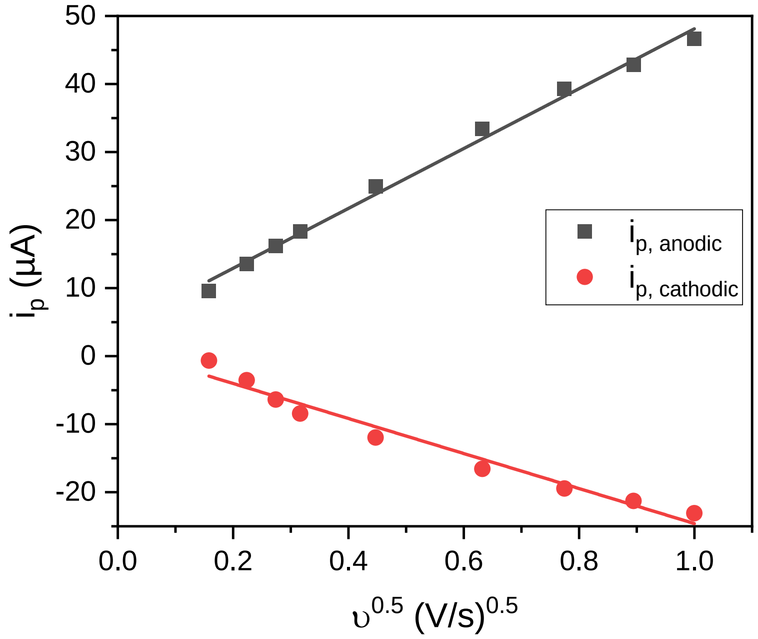

Figure S50. Anodic and cathodic peak currents of 0.5 mM **BTX-24c8** in DCM, 100 mM $TBAPF_6$ as a function of the square root of scan rate including linear fits.

5.2 Voltammetric Cation Binding Studies

To investigate the influence of cation binding on the redox (switching) properties of the receptors, systematic electrochemical titration studies were carried out for all four **BTX-crowns** with Na^+, K^+, NH_4^+ and DBA^+ (all as PF_6^- salts) in CH_3CN/CH_2Cl_2 7:3 (v/v), 100 mM $TBAPF_6$. All experiments were carried out at a constant ionic strength of 100 mM, to ensure that (counter) ion pairing effects are constant and do not contribute to shifts in redox potentials.

As shown in Figure 5B and Figures S51, cation addition induced well-defined, anodic voltammetric shifts of the oxidation wave of the **BTX-crown ethers** in almost all cases. The maximum shift of this cation-induced response is collated in Table S5 and reveals notable differences between the different receptors. Specifically, **BTX-15c5** displayed the smallest response towards Na^+, K^+ and NH_4^+, while **BTX-18c6** ***anti*** displayed much larger voltammetric shifts with notable response preference towards the latter two cations (ΔE_{max} up to 38 mV).

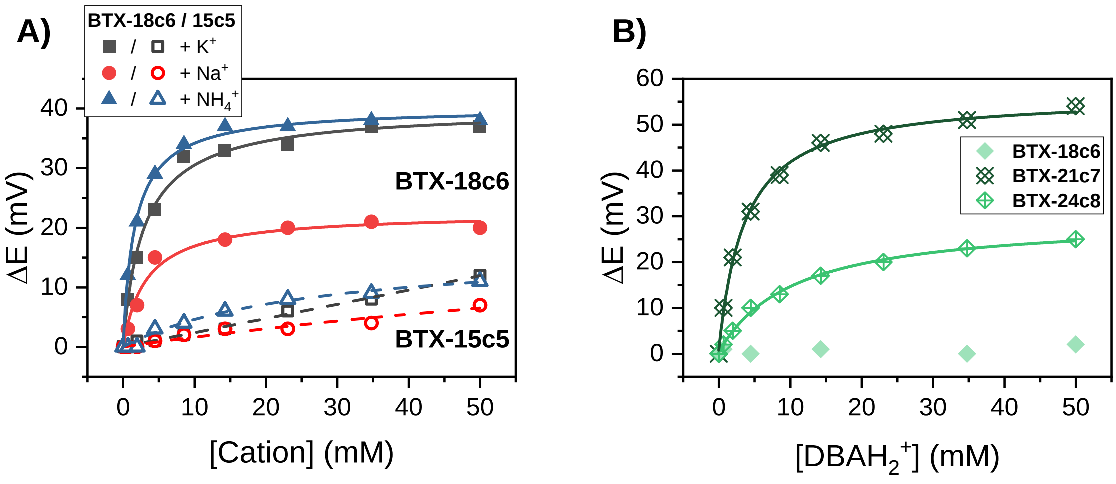

Figure S51. A) and B) Anodic voltammetric shifts of BTX-crowns in CH_3CN/CH_2Cl_2 7:3, 100 mM $TBAPF_6$ upon addition of increasing concentrations of various cations, see also Figure S52. The solid lines represent fits according to a 1:1 host-guest stoichiometric Nernst binding model (eqn. S1).

Table S5. Maximum anodic shifts ΔE_{max} (mV) of oxidation peak of **BTX-crown ethers** (all in the *anti*-folded state) in CH_3CN/CH_2Cl_2 7:3, 100 mM $TBAPF_6$.

	**BTX-15c5**	**BTX-18c6**	**BTX-21c7**	**BTX-24c8**
**Na^+**	7	21	11	17
**K^+**	12	37	15	18
**NH_4^+**	11	38	20	27
**DBA^+**	n.d.	<2	54	25

Interestingly, a further extension of the crown ether strap in **BTX-21c7*anti*** did not lead to a further increase in ΔE_{max} towards these inorganic cations, but instead a significant decrease with the largest perturbation observed for NH_4^+ (ΔE_{max} = 20 mV). The even larger **BTX-24c8*anti***

displays somewhat enhanced responses towards these cations in comparison to **BTX-21c7*anti*,** such that there is a notable odd/even effect with enhanced responses for **BTX-24c8*anti*** and **BTX-18c6*anti*,** the latter of which, however, remains the best voltammetric sensor of this family for the inorganic cations. Interestingly, while **BTX-15c5*anti*** and **BTX-18c6*anti*** display strictly monotonic response isotherms, indicative of 1:1 stoichiometric host-guest complexation, both of the larger **BTX-21c7*anti*** and **BTX-24c8*anti*** show some deviations from this behavior, with slightly decreasing responses at high concentrations of Na^+ and NH_4^+, potentially reflective of formation of higher order host-guest complexes in the presence of excess of these small cations (see Figure S52).

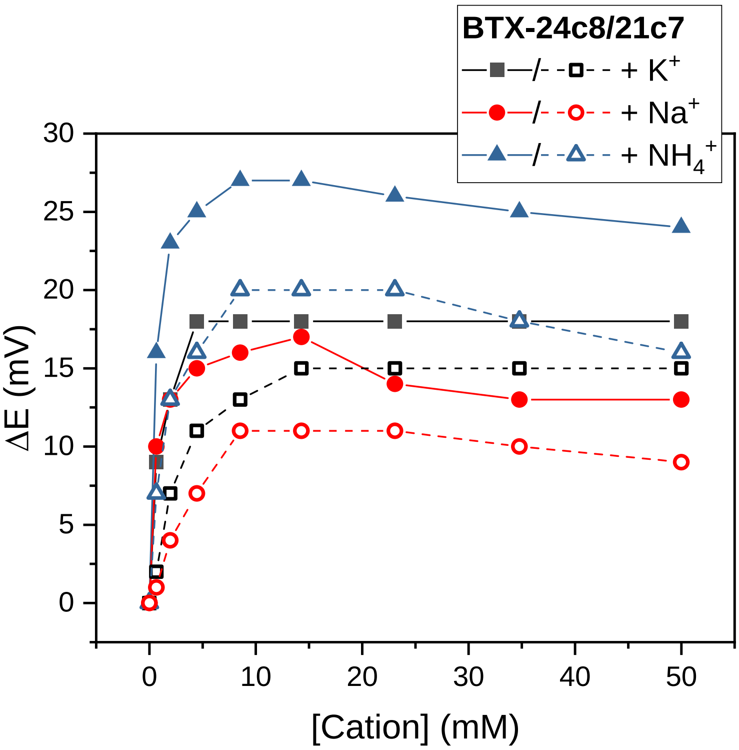

Figure S52. Anodic voltammetric shifts of **BTX-21c7** (empty symbols) and **BTX-24c8** (filled symbols) in CH_3CN/DCM 7:3, 100 mM $TBAPF_6$ upon addition of increasing concentrations of various cations.

We further investigated the voltammetric response of the receptors towards the larger DBA^+, which, as discussed, forms pseudorotaxanes with **BTX-21c7** and **BTX-24c8** only. This is also reflected in their potential shift magnitude, which is larger for the former host with ΔE_{max} of up to 54 mV, the largest response of all tested cation/host combinations (Figure S51B). For **BTX-24c8*anti*** ΔE_{max} is notably smaller (25 mV), but still significant. In contrast, **BTX-18c6*anti*** displays no redox response to DBA^+ in good agreement with lack of binding as attested by ^1H NMR studies.

Importantly, in all cases the CVs did, apart from the continuous anodic shifts of the oxidation peak, not otherwise significantly change during the titration experiments, indicating that the redox-driven conformational switching of the BTX core is, apart from the potential shift,

unaffected by cation binding. This is to say that even in the presence of an initially bound cation, the switch can undergo significant geometric changes from the neutral *anti*-folded to the dicationic orthogonal state. The fact that the reduction peak is largely insensitive to cation presence and does not display any anodic voltammetric shifts is also indicative that cation binding to the dicationic, orthogonal switch state is negligible (as independently confirmed by ^{1}H NMR studies, see Section S4.2).

However, it is important to note that neither the absolute response magnitude nor the slope of the voltammetric binding isotherms are directly reflective of the cation binding constant to either *anti*-folded or dicationic oxidation state, as under fast exchange conditions, the magnitude of the voltammetric shift is theoretically dependent on the ratio of binding constants to both oxidation states, as discussed in the following:[17]

The voltammetric binding isotherms shown in Figure S51 were fitted according to the following 1:1 host-guest stoichiometric Nernst binding model (eqn. S1),[17-19] where R, T and F have their usual meaning, n is the number of transferred electrons and K_{Red} and K_{Ox} are the (cation) binding constants to the oxidized and reduced receptor state, respectively.

$$\Delta E = -\frac{RT}{nF}\ln\left(\frac{K_{Ox}}{K_{Red}}\right) \quad \text{eqn. S1}$$

As shown in Figure S51, this afforded good fits for **BTX-15c5** and **BTX-18c6** (with n = 1, *vide infra*), with $K_{Red} > K_{Ox}$ in all cases, indicative of weaker binding to the oxidized receptor state, as expected based on, among others, electrostatic arguments. K_{Red} represents the cation binding constant to the neutral state (*i.e.* **BTX-crown ether$_{anti}$**), however the interpretation of the meaning of K_{Ox} (and which receptor state it can be ascribed to) is more difficult. The oxidative wave corresponds, macroscopically, to the two-electron oxidation of BTX_{anti} to BTX^{2+}, however this is not strictly a concerted two-electron transfer. Specifically, oxidation of BTX most likely proceeds via an E_1CE_2 mechanism; one-electron oxidation of BTX_{anti} (at E_1) generates the mono radical cation of BTX in the same *anti*-folded state ($BTX_{anti}^{+\bullet}$). This species then rapidly geometrically rearranges to a twisted conformer ($BTX_{twisted}^{+\bullet}$, "C" step), whose oxidation to BTX^{2+} (E_2) is more facile than the initial oxidation, *i.e.* $E_2 < E_1$ (potential inversion). As a result, applying an electrode potential that is sufficient to enable the first oxidation ($E_{applied} \geq E_1$) also immediately forces the second oxidation (E_2). However, the peak potential is solely determined by E_1. As such, we propose that the shift of the oxidation potential ΔE observed upon addition of cations is purely reflective of the change in E_1 upon cation binding, that is the $BTX_{anti}/BTX_{anti}^{+\bullet}$ redox couple. Hence, K_{Ox} most likely reflects the cation binding constant to $BTX_{anti}^{+\bullet}$ and hence only reports on electrostatic contributions to the decrease in cation binding strength upon (one-electron) oxidation (*i.e.* it does not report on additional changes in cation binding strength brought about by conformational rearrangements (or the second oxidation)). This has implications in the use of these systems as voltammetric sensors,

where a larger ΔE (*i.e.* a larger ratio in binding constants upon oxidation, see eqn. S1) is generally desired.[17, 20]

As a result of these complicated binding and conformational equilibria we herein refrain from a further quantitative analysis and interpretation of these “voltammetric” binding constants obtained by eqn. S1.

6. Computational Studies

The calculation procedure described in SI Section 1 (page S3-4) allowed the determination of Boltzmann-averaged Gibbs free energies for every studied structure (*syn*- and *anti*-folded isomers of **BTX-21c7** and **BTX-24c8**, with and without the DBA^+ guest), see Table S6.

Table S6. Calculated Boltzmann-averaged Gibbs free energies differences (ΔG) between *syn*- and *anti*-folded isomers of free and DBA^+ complexes BTX-crown. Values are given in kJ/mol at 25 °C.

	BTX-21c7	BTX-21c7•DBA$^+$	BTX-24c8	BTX-24c8•DBA$^+$
***Anti*-folded**	0	0	0	0
***Syn*-folded**	33.9	46.4	34.0	29.9

These calculated values are in-line with the behaviour observed experimentally, with the *syn*-folded state being significantly higher in energy than its *anti*-folded counterpart for both the free host as well as the DBA^+ complexes (ΔG = 30 to 46 kJ/mol). As expected, for the free hosts, the energy difference between the *anti* and *syn*-folded states is virtually identical for **BTX-21c7** and **BTX-24c8**. In contrast, DBA^+ stabilises the *syn*-folded state of **BTX-24c8**, but destabilises **BTX-21c7*syn***.

Further information about the relative binding constants of both folded states of **BTX-21c7** and **BTX-24c8** with the DBA^+ cation can also be extracted from these calculations. Specifically, the binding free energy can be calculated from the (experimental) binding constant as follows:

$$\Delta G_{bind} = -RT\ln(K_{bind})$$

This binding free energy can be decomposed as follows:

$$\Delta G_{bind} = G_{complex} - G_{receptor} - G_{guest}$$

In order to compare trends in binding ability of DBA^+ by the various isomers of the **BTX-crown ethers**, the difference in binding free energy can be evaluated:

$$\Delta\Delta G_{bind} = \Delta \mathrm{G}_{\mathrm{bind2}} - \Delta \mathrm{G}_{\mathrm{bind1}}$$

$$\Leftrightarrow \Delta\Delta G_{bind} = G_{complex2} - G_{receptor2} - G_{guest2} - (G_{complex1} - G_{receptor1} - G_{guest1})$$

Which can be simplified in the case of an identical guest (here DBA^+):

$$\Delta\Delta G_{bind} = G_{complex2} - G_{receptor2} - G_{complex1} + G_{receptor1}$$

The binding free energies differences of the various **BTX-crown ether** isomers $\Delta\Delta G_{bind}$ can thus be evaluated from the experimental binding constants, but also from the calculated Gibbs free energies of the free and complexed receptors, both of which are collated in Table S7.

Table S7. Calculated Boltzmann-averaged binding free energies differences ($\Delta\Delta G_{bind}$) of the DBA^+ complexes of both folded isomers of the **BTX-crown ethers**. The values in parenthesis were obtained from the binding constants determined experimentally. Values are given in kJ/mol at 25 °C.

	**BTX-21c7•DBA⁺**	**BTX-24c8•DBA⁺**
***Anti*-folded**	0 (0)	5.2 (3.8)
***Syn*-folded**	12.6 (9.5)	1.1 (1.3)

The calculated binding free energy differences not only match very well with the experimental ones, but further confirm that DBA^+ binding to **BTX-21c7** is strongly suppressed upon irradiation, while the opposite trend (albeit with slightly smaller switching magnitude) is observed for **BTX-24c8**. Even though these DFT calculations can clearly reproduce the experimental binding constants very well, we did not manage to extract chemically interpretable structural parameters from the calculated lowest energy conformers of the host-guest complexes that could explain the relative binding trends. Specifically, we thoroughly compared numerous structural parameters, including multiple distances between host and guest, H-bonds length, angles and dihedrals, but could not identify any reliable trends. In fact, the structure of the most stable conformers of the complexes that we calculated at all isomeric states are extremely similar if we omit the difference in length of the macrocyclic chain.

7. References

1. Thordarson, P., Determining association constants from titration experiments in supramolecular chemistry. *Chem. Soc. Rev.* **2011,** *40*, 1305-1323.
2. Pääkkönen, J.; Jänis, J.; Rouvinen, J., Calculation and Visualization of Binding Equilibria in Protein Studies. *ACS Omega* **2022,** *7*, 10789-10795.
3. Grimme, S., Exploration of chemical compound, conformer, and reaction space with meta-dynamics simulations based on tight-binding quantum chemical calculations. *J. Chem. Theory Comput.* **2019,** *15*, 2847-2862.
4. Pracht, P.; Bohle, F.; Grimme, S., Automated exploration of the low-energy chemical space with fast quantum chemical methods. *Phys. Chem. Chem. Phys.* **2020,** *22*, 7169-7192.
5. Pracht, P.; Grimme, S.; Bannwarth, C.; Bohle, F.; Ehlert, S.; Feldmann, G.; Gorges, J.; Müller, M.; Neudecker, T.; Plett, C., CREST—A program for the exploration of low-energy molecular chemical space. *J. Chem. Phys.* **2024,** *160*.
6. Bannwarth, C.; Ehlert, S.; Grimme, S., GFN2-xTB—An accurate and broadly parametrized self-consistent tight-binding quantum chemical method with multipole electrostatics and density-dependent dispersion contributions. *J. Chem. Theory Comput.* **2019,** *15*, 1652-1671.
7. Grimme, S.; Bohle, F.; Hansen, A.; Pracht, P.; Spicher, S.; Stahn, M., Efficient quantum chemical calculation of structure ensembles and free energies for nonrigid molecules. *J. Phys. Chem. A* **2021,** *125*, 4039-4054.
8. Neese, F.; Wennmohs, F.; Becker, U.; Riplinger, C., The ORCA quantum chemistry program package. *J. Chem. Phys.* **2020,** *152*.
9. Grimme, S.; Hansen, A.; Ehlert, S.; Mewes, J.-M., r2SCAN-3c: A “Swiss army knife” composite electronic-structure method. *J. Chem. Phys.* **2021,** *154*.

10. Marenich, A. V.; Cramer, C. J.; Truhlar, D. G., Universal solvation model based on solute electron density and on a continuum model of the solvent defined by the bulk dielectric constant and atomic surface tensions. *J. Phys. Chem. B* **2009,** *113*, 6378-6396.
11. Corbet, B. P.; Wonink, M. B. S.; Feringa, B. L., Fast synthesis and redox switching of di- and tetra-substituted bisthioxanthylidene overcrowded alkenes. *Chem. Commun.* **2021,** *57*, 7665-7668.
12. Bonger, K. M.; van den Berg, R. J.; Heitman, L. H.; IJzerman, A. P.; Oosterom, J.; Timmers, C. M.; Overkleeft, H. S.; van der Marel, G. A., Synthesis and evaluation of homo-bivalent GnRHR ligands. *Biorg. Med. Chem.* **2007,** *15*, 4841-4856.
13. Liu, Y.; Zhang, Q.; Crespi, S.; Chen, S.; Zhang, X. K.; Xu, T. Y.; Ma, C. S.; Zhou, S. W.; Shi, Z. T.; Tian, H., Motorized Macrocycle: A Photo-responsive Host with Switchable and Stereoselective Guest Recognition. *Angew. Chem.* **2021,** *133*, 16265-16274.
14. Ashton, P. R.; Chrystal, E. J.; Glink, P. T.; Menzer, S.; Schiavo, C.; Spencer, N.; Stoddart, J. F.; Tasker, P. A.; White, A. J.; Williams, D. J., Pseudorotaxanes formed between secondary dialkylammonium salts and crown ethers. *Chem. Eur. J,* **1996,** *2*, 709-728.
15. Bell–Evans–Polanyi principle. In *Bell–Evans–Polanyi principle*, 3.0.1 ed.; International Union of Pure and Applied Chemistry (IUPAC): 2019.
16. Dasgupta, S.; Wu, J., Formation of [2]rotaxanes by encircling [20], [21] and [22]crown ethers onto the dibenzylammonium dumbbell. *Chem. Sci.* **2012,** *3*, 425-432.
17. Hein, R.; Beer, P. D.; Davis, J. J., Electrochemical Anion Sensing: Supramolecular Approaches. *Chem. Rev.* **2020,** *120*, 1888-1935.
18. Hein, R.; Docker, A.; Davis, J. J.; Beer, P. D., Redox-Switchable Chalcogen Bonding for Anion Recognition and Sensing. *J. Am. Chem. Soc.* **2022,** *144*, 8827-8836.
19. Oliveira, R.; Groni, S.; Fave, C.; Branca, M.; Mavre, F.; Lorcy, D.; Fourmigue, M.; Schollhorn, B., Electrochemical activation of a tetrathiafulvalene halogen bond donor in solution. *Phys. Chem. Chem. Phys.* **2016,** *18*, 15867-15873.
20. Hein, R.; Li, X.; Beer, P. D.; Davis, J. J., Enhanced voltammetric anion sensing at halogen and hydrogen bonding ferrocenyl SAMs. *Chem. Sci.* **2021,** *12*, 2433-2440.
